# Oligosaccharide Block Copolymers with Branched Architectures and Channel Energy Level Optimizations for High-Performance Floating Gate Phototransistor Memory

**DOI:** 10.1021/acsami.5c13139

**Published:** 2025-09-15

**Authors:** Ping-Jui Yu, Wei-Cheng Chen, Ya-Shuan Wu, Bi-Hsuan Lin, Yan-Cheng Lin, Redouane Borsali, Wen-Chang Chen

**Affiliations:** † Department of Chemical Engineering, 33561 National Taiwan University, Taipei 10617, Taiwan; ‡ University of Grenoble Alpes, CERMAV-CNRS, 38000 Grenoble, France; § National Synchrotron Radiation Research Center, Hsinchu 300092, Taiwan; ∥ Department of Chemical Engineering, 34912 National Cheng Kung University, Tainan 70101, Taiwan; ⊥ Advanced Research Center for Green Materials Science and Technology, National Taiwan University, Taipei 10617, Taiwan

**Keywords:** photomemory, carbohydrate polymers, nonvolatile memory, rylenediimides, poly(dimethylsiloxane)

## Abstract

The nonvolatile phototransistor memory features a fast transmission speed, low latency, and nondestructive orthogonal operation for multibit data storage. Utilizing perovskite quantum dots (QDs) with polymers has been regarded as a facile and efficient approach to fabricating phototransistor memory devices due to their high light responsivity and nonvolatility. In addition, introducing block copolymers improves the QD dispersion and ambient stability. However, the reported study has not investigated the branching architectures of block copolymers (BCPs) influencing memory behavior and the tunability between the channel and the floating gate memory layer. Herein, this study utilizes different numbers of branching arms of carbohydrate-based BCPs, comprising poly­(dimethylsiloxane) (PDMS, as A block) and maltotriose (MT, as B block), to promote electrical performance for phototransistor memory. Different rylenediimide-based N-type semiconductors are combined with BCP/QD floating gate dielectrics according to their energy levels. Due to the most substantial QD accommodation conferred by BCPs, the triarms BCP with QD (**AB3QD**) exhibited the smoothest surface among all BCP/QD nanocomposites, with the best electrical performance for phototransistor memory. Furthermore, the naphthalene diimide (**NDI)**-based device exhibits the most suitable energy levels for adapting the floating gate layer, resulting in good charge transfer efficiency, photoresponse, and memory stability. The reason can be attributed to the comparable lowest unoccupied molecular orbital (LUMO) energy level for transferring negative charges and the low-lying highest occupied molecular orbital (HOMO) energy level for blocking positive charges, compared to the energy levels of the QD. As an aspect of the device performance, the phototransistor memory renders a high memory ratio of *I*_ON/OFF_ = 3.09 × 10^5^, which outperforms those of rylenediimides such as perylene diimide (**PDI**, *I*_ON/OFF_ = 4.48 × 10^4^) and pyromellitic diimide (**PMDI**, negligible *I*_ON/OFF_), as well as the diarms (*I*_ON/OFF_ = 1.33 × 10^5^) or linear (*I*_ON/OFF_ = 5.06 × 10^4^) BCP counterparts. Additionally, the device exhibits good stability (*I*_ON/OFF_ > 10^6^ over 10,000 s) and decent switchability (*I*_ON/OFF_ > 10^5^ over 10 cycles). In conclusion, the results indicate that the different branching BCP architectures and energy level alignments between the channel and floating gate layers play a vital role in phototransistor memory.

## Graphical abstract

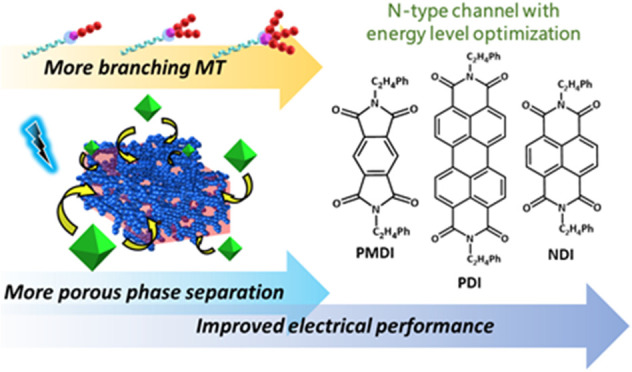

## Introduction

Recent advances show that memory has become a crucial component in the semiconductor industry,^1,2^ driven by the growing demand for high-performance data processing and storage. The rapid development of the Internet of Things (IoT) and artificial intelligence (AI) demands high-capacity digital storage, low energy consumption, and rapid computational speed.^3,4^ However, the “memory wall” bottleneck in digital technology remains an issue, as it is characterized by high computing latency, limited bandwidth, and insufficient digital storage.^5–8^ Organic field-effect transistor (OFET) memory, a traditional type of nonvolatile memory, has attracted significant research interest due to its low manufacturing cost, low-temperature processing, nondestructive readout, lightweight, and good compatibility with complementary integrated circuits.^9–13^ Various functionalities of OFET memory take advantage of electrets, which can be categorized into floating gate,^13,14^ ferroelectric,^15,16^ and polymer electret-based OFET memory.^17,18^ To address the challenge of memory capacity, nonvolatile phototransistor memory offers a promising solution due to its large memory window, high memory ratio, and unique information storage and removal with orthogonally electrical/optical-driven operations.^19–21^ The memory states can be maintained without voltages, significantly reducing energy consumption compared to volatile memory applications. However, complex heterojunctions and memory layer designs necessitate improved interfacial properties and charge transfer mechanisms to achieve high performance.

The developing strategies in phototransistor memory can be categorized into (i) semiconductor heterojunctions, (ii) polymer electrets, and (iii) floating gate dielectrics. By blending organic semiconductors with light-absorbing materials or multilayer heterojunctions (sandwich architecture), the heterojunction structure can provide good charge transport while incorporating the photoresponse capability.^22–24^ For example, Tsai et al. prepared a single-layer heterostructure composed of hybrid perovskite quantum dots (PVSK QDs) and conjugated polymer composite films, leading to not only simplifying the device architecture but also amplifying the area of the heterojunction.^22^ Alternatively, multilayer heterojunctions are designed by incorporating a charge-trapping layer proximal to the channel layer, allowing for a more flexible selection of materials.^25^ Kuang et al. proposed a self-assembly constraint on PVSK QDs, promoting the photogenerated carrier injection efficiency from light-sensitive molecules due to the well-defined interfacial surface with the channel layer.^24^ Regarding the polymer electret design, the device requires suitable mechanical properties and a simple interface with the channel layer.^25–27^ For example, Prakoso et al. developed a series of photoactive polymers with different conjugated acenes as a chargeable and light-absorbing electret.^28^ Furthermore, some studies utilized block copolymers (BCPs) as electrets in optoelectronic memory, benefiting from their self-assembly behavior, which is governed by the fraction volume, degree of polymerization, and incompatibility between different blocks. For example, Mulia et al. demonstrated microwave solvent-assisted annealing treatment of high-χ carbohydrate BCPs, specifically polystyrene, clicked with maltotriose, maltoheptaose, and cyclodextrin, endowing a promising approach for the sustainable and environmentally friendly optoelectronic memory.^29^ The low-*N* high*-*χ BCP is a good strategy to gain the microphase separated structure without compromising the *d*-spacing. Because the ordered diblock copolymers generally required the value of χ*N* to be over the critical value of 10.5, simply lowering *N* would result in a disordered state. Furthermore, the *d*-spacing of the self-assembled nanostructure is described as *d* ∼ χ^1/6^ *N* ^2/3^, where χ is the Flory–Huggins parameter and *N* is the degree of polymerization, implying that a lower *N* can decrease the *d*-spacing. Therefore, in this study, the BCP, based on the enhanced hydrophilicity of oligosaccharide and the stronger hydrophobicity of PDMS compared to polyisoprene and polystyrene, can easily self-assemble into an ordered nanostructure.

Also, the oligosaccharide biomaterials contributed the chargeable property to memory electronics due to their abundant hydroxy groups. In the past few decades, Raeis-Hosseini et al. utilized chitosan-based nanocomposite films on nonvolatile memristors, where the inorganic moiety was electrically stimulated following the excitation of charges trapped by the rich hydroxyl groups.^30,31^ Chen et al. adopted maltose-based materials as the memory layer for optoelectronics, where light illumination or electrical impulses excited the photo- or electrical-responsive semiconducting materials, and the trapped charges were also trapped in the maltose-based materials.^32–34^ These green technologies demonstrated that oligosaccharides can pave the way toward sustainable development without compromising performance.

To further improve the phototransistor memory performance, floating gate dielectrics are introduced. By combining light-absorbing nanomaterials in a polymer matrix, photoresponsive floating gate dielectrics can be developed. For instance, Zhang et al. recently developed a floating gate dielectric comprising PbS QD/poly­(methyl methacrylate) (PMMA) hybrids.^35^ The synaptic phototransistor can produce photoresponses and light-modulated plasticity across the ultraviolet-to-near-infrared light spectrum. Furthermore, self-assembled BCP can regulate PVSK growth, which confers a low energy consumption and ultrafast operation in synaptic phototransistors. The BCP morphology is essential in determining the dispersion and size of PVSKs.^36–38^ Despite these advancements, the various BCP architectures influencing phototransistor memory have never been investigated. Concerning the semiconductor channel, previous studies have revealed that the design of defective^39,40^ or phase-change properties^41–43^ can promote the phototransistor memory performance. However, the energy level effects of the semiconductor channel have never been systematically investigated. Optimization between the semiconductor channel and the floating gate layers requires further corroboration.

In this study, carbohydrate BCPs were synthesized, consisting of poly­(dimethylsiloxane) (PDMS, as the A block) and maltotriose (MT, as the B block), forming **AB**-, **AB2**-, and **AB3**-type BCPs, as shown in Scheme 1. PVSK QDs were selected as photoresponsive electrets due to their solution processability, tunable light absorption, monodispersed size, and high stability. By blending QDs with different BCPs, the influence of BCP architecture on the distribution and the size of QDs was investigated to improve the photoresponse and memory performance. For the channel layer, the well-stacking and high electron mobility rylenediimide-based semiconductor molecules, 2,7-diphenethylbenzo­[lmn]­[3,8]­phenanthroline-1,3,6,8­(2*H*,7*H*)-tetraone (**NDI**), 2,9-diphenethylanthra­[2,1,9-def:6,5,10-d′e′f′]­diisoquinoline-1,3,8,10­(2*H*,9*H*)-tetraone (**PDI**), and 2,6-diphenethylpyrrolo­[3,4-*f*]­isoindole-1,3,5,7­(2*H*,6*H*)-tetraone (**PMDI**), were regarded as good choices for the channel layers to fine-tune the energy level alignment with the floating gate dielectrics. The chemical structures of the molecules and device architecture are displayed in Figure 1a. After the BCPs studied were synthesized through an azide–alkyne cycloaddition click reaction, their thermal properties were characterized by thermogravimetric analysis (TGA) and differential scanning calorimetry (DSC). The morphologies were characterized by atomic force microscopy (AFM), transmission electron microscopy (TEM), and grazing incidence X-ray diffraction. For the optical properties, a series of optical characteristics were investigated using ultraviolet–visible absorption spectroscopy (UV–vis), steady-state photoluminescence, and time-resolved photoluminescence (TRPL). This study demonstrated that the increased branching of hydrophilic MT arms in BCP affects the heterojunction morphology and improves the photoresponsivity of the floating gate dielectrics. Additionally, this study revealed the compatibility of energy levels between the channel layer and the floating gate memory layer.

**Scheme 1:**
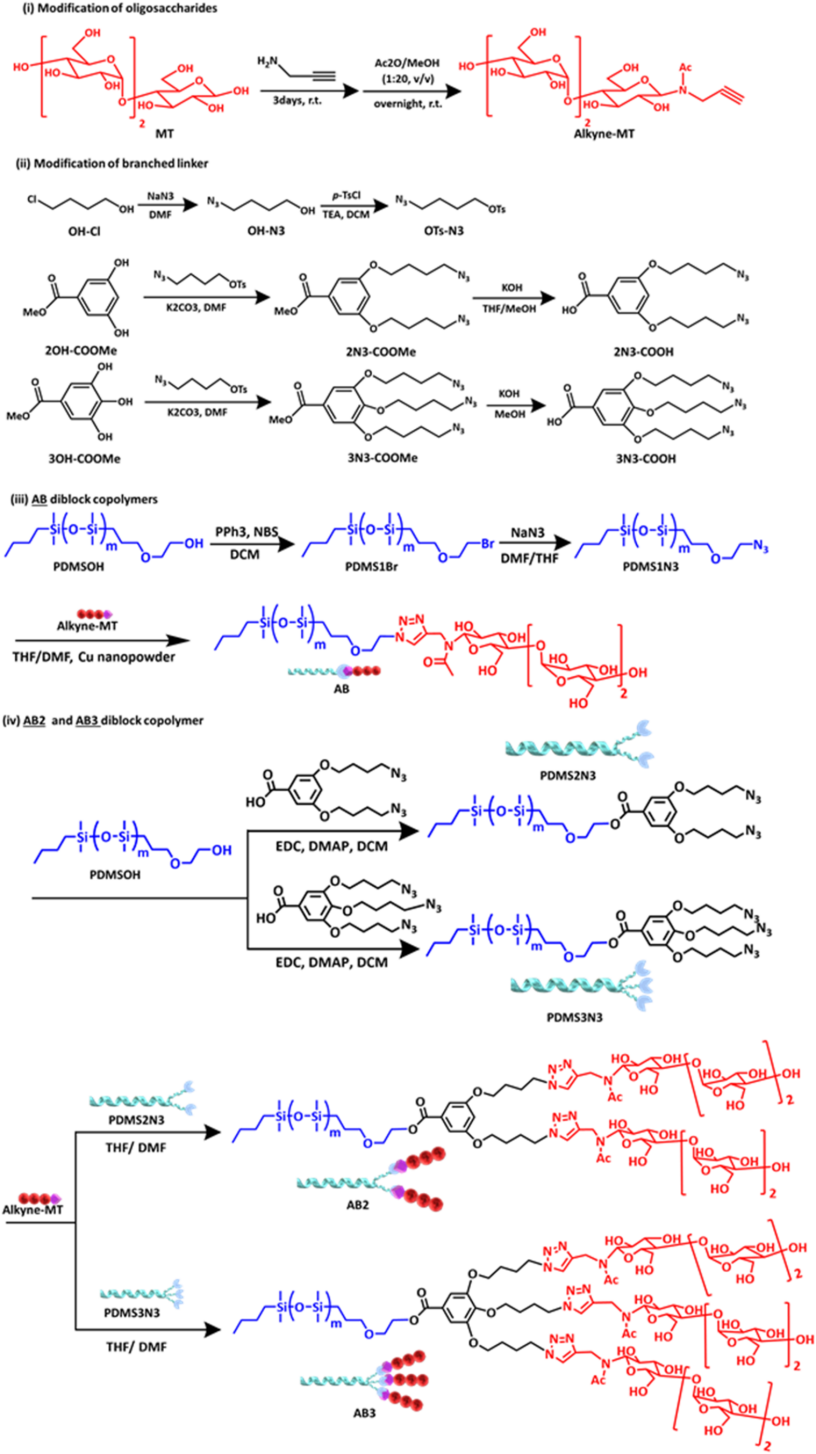
Synthetic Routes of (i) Modification of Oligosaccharides: **Alkyne-MT**, (ii) Modification of the Branched Azide Linkers, (iii) **AB** Linear-Type BCP, and (iv) **AB2** and **AB3** Branched-Type BCPs

**Figure 1:**
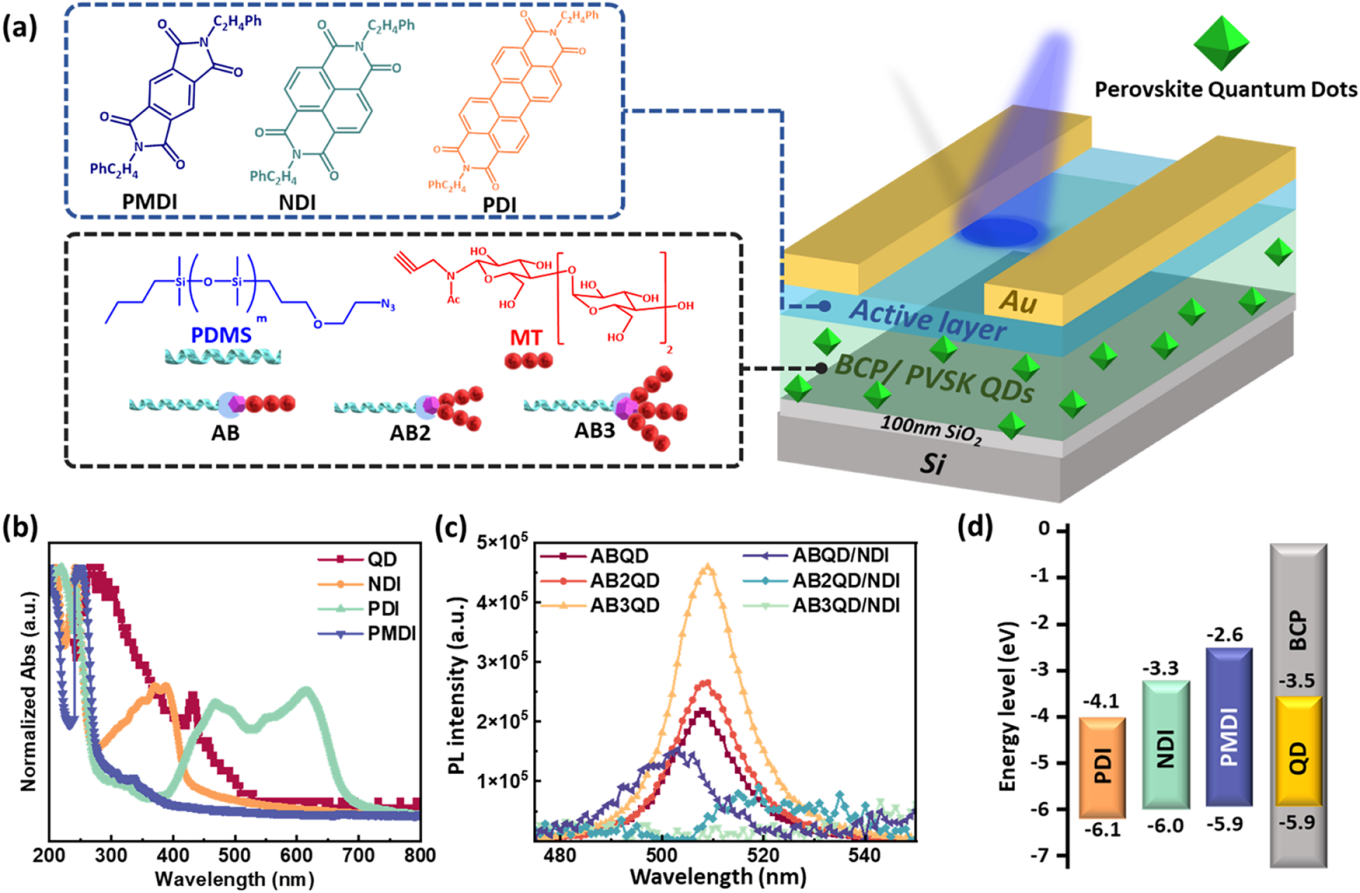
(a) Chemical structures, (b) UV–vis absorption, (c) PL emission spectra, and (d) frontier energy level alignment of the constituent materials in the phototransistor memory, including (a, b, d) the N-type channel and QD and (c, d) floating gate layers comprising the BCPs and QD. Note that the PL emissions of the BCP/QD films were displayed with their **NDI**-stacked counterparts to characterize their PL quenching effect. The combinations of BCP**/**QD are denoted as **ABQD**, **AB2QD**, and **AB3QD**, with BCPs of **AB**, **AB2**, and **AB3**, respectively.

## Experimental Section

### Materials

Perovskite quantum dots (QDs, oleic acid and oleyamine-coated, 10 mg mL^–1^ in toluene), propargylamine, acetic anhydride (Ac_2_O), chlorobutanol, *p*-toluenesulfonyl chloride, triethylamine (TEA), potassium carbonate (K_2_CO_3_), *N*-bromosuccinimde (NBS), triphenylphosphine (PPh_3_), *N*-(3-(dimethylamino)­propyl)-*N*′-ethylcarbodiimide (EDC), 4-(dimethylamino)­pyridine (DMAP), sodium azide (NaN_3_), copper nanopowder, and all other reagents were purchased from Sigma-Aldrich and used as received without further purification. Methyl gallate (**3OH–COOMe**) and methyl 3,5-dihydrobenzoate (**2OH–COOMe**) were purchased from TCI, Inc. For poly­(dimethylsiloxane) (PDMS) with a number-averaged molecular weight (*M*_n_) of 1000 g mol^–1^, ω-hydroxy-terminated PDMS (**PDMS1OH**) was purchased from Gelest, Inc. Maltotriose (**MT**) was purchased from Nagase & Co., Ltd. 2,7-Diphenethylbenzo­[lmn]­[3,8]­phenanthroline-1,3,6,8­(2*H*,7*H*)-tetraone (**NDI**), 2,9-diphenethylanthra­[2,1,9-def:6,5,10-d’e’f’]­diisoquinoline-1,3,8,10­(2*H*,9*H*)-tetraone (**PDI**), and 2,6-diphenethylpyrrolo­[3,4-*f*]­isoindole-1,3,5,7­(2*H*,6*H*)-tetraone (**PMDI**) were acquired from Luminescence Technology Corp. The general synthetic approach of the branched block copolymers (BCPs) is presented in Scheme 1, and the synthetic details are described in the Supporting Information.

### Chemical Structure Characterization

^1^H and ^13^C spectra of the monomers and polymers were recorded on a Bruker Avance 400 MHz spectrometer. The size exclusion chromatograph (SEC) was recorded in an Enshine SUPER CO-150 with polystyrene gel columns (Stryagel HR2 and Stryagel 4), and the weight-/number-average molecular weights (*M*_w_/*M*_n_) and corresponding polydispersity (*Đ*_M_) of BCPs were studied with DMF as the eluent at 1.0 mL min^–1^. Thermogravimetric analysis (TGA, TA Instruments TGA 55) and differential scanning calorimetry (DSC, TA Instruments DSC 25) were employed to investigate the thermal properties of the BCPs. A CHI 6273E electrochemical analyzer with a three-electrode method was used to perform cyclic voltammetry (CV), wherein indium tin oxide (ITO) substrates coated with studied BCPs, a platinum wire, and Ag/AgNO_3_ were used as the working electrode, auxiliary electrode, and reference electrode, respectively. The electrolyte was made of 0.1 M tetrabutylammonium perchlorate (TBAP) in anhydrous acetonitrile.

### Thin Film and Device Fabrications

All BCPs were dissolved in 10 mg mL^–1^ toluene, and 10 mg mL^–1^ QDs in toluene were added together overnight. The solution of BCPs/QD was prepared at the ratios of 9/1, 8/2, and 7/3. The photomemory was fabricated by using the bottom-gate/top-contact (BG/TC) configuration. A silicon wafer substrate with a 100 nm thermally grown SiO_2_ dielectric layer was treated with plasma for 15 min after the cleaning process using isopropanol, methanol, and acetone. The BCP/QD solution was filtered via 0.22 μm PTFE filters and spin-coated onto the plasma-pretreated substrate at a spin rate of 1000 rpm for 60 s, followed by thermal annealing at 80 °C for 2 h under vacuum. Next, the channel layer was thermally deposited with a thickness of 50 nm at a growth rate of 0.02 nm s^–1^ under 10^–7^ Torr. Finally, the gold channel electrodes were deposited 70 nm thick through the shadow mask, with channel lengths (*L*) and widths (*W*) defined as 50 and 1000 μm, respectively.

### Morphology, Optical, and Device Characterization

The surface morphology of thin films was investigated by atomic force microscopy (AFM, Bruker Innova) in tapping mode, and the corresponding images were processed with Nanoscope software. The thicknesses of the thin films were determined by an Optical Thickness Meter (OPTM) from Otsuka Electronics Inc. The grazing incidence wide-angle X-ray scattering (GIWAXS) and grazing incidence small-angle X-ray scattering (GISAXS) analyses were obtained from the beamline 23A1 in the National Synchrotron Radiation Research Center (NSRRC), Taiwan. An ultraviolet–visible (UV–vis) absorption spectrum (Hitachi U-4100 spectrophotometer) provided the optical absorbance spectra of the hybrid films. The thin film’s photoluminescence (PL) emission spectra were recorded using a HORIBA fluorolog-3 spectrometer. The time-resolved photoluminescence (TRPL) spectra were obtained using a Hamamatsu Universal Streak Camera C10910 and an M10913 slow single-sweep unit in NSRRC, Taiwan. The phototransistor memory analysis was characterized using a Keithley 4200-SCS semiconductor parameter analyzer and a Keithley 2634B instrument (Keithley Instrument Inc., Cleveland, OH, USA) in a nitrogen-filled glovebox under dark conditions.

## Results and Discussion

### Synthesis and Chemical Structure Characterization of the Block Copolymers Studied

Scheme 1 illustrates the synthetic routes of **AB**-, **AB2**-, and **AB3**-type BCPs, comprising PDMS (A block) and MT (B block), resulting in the volume fractions (*f*_A_) of PDMS being 0.77, 0.63, and 0.53, respectively. First, oligosaccharide, MT, was modified via reductive amination with propargylamine and *N*-acetylation with acetic acid overnight to obtain **alkyne-MT**. For linear-type **AB**, after bromination and azidation were carried out on **PDMSOH** to give **PDMS1N3**, **AB** was formed through the click reaction of **PDMS1N3** and **alkyne-MT**. For the branched-type BCPs, **AB2** and **AB3** were started by modifying the branched linker, which was implemented by first converting **OH-Cl** to **OH-N3**, followed by tosylation to yield **OTs-N3** (Figures S1 and S2 in the Supporting Information). Then, **2N3-COOMe** and **3N3-COOMe** were obtained via Williamson ether synthesis from **2OH–COOMe** and **3OH–COOMe**, respectively. The chemical structure characterization of the difunctionalized and trifunctionalized azide precursors is presented in Figures S3–S6 (Supporting Information). Subsequently, the precursor was saponified to afford **2N3-COOH** and **3N3-COOH**. The di- and trifunctionalized azide linkers after saponification are characterized in Figures S7–S10 (Supporting Information). **PDMSOH** was directly functionalized to azide, as shown in Figures S11–S13 (Supporting Information). **PDMSOH** was also esterified with these branched linkers to obtain **PDMS2N3** and **PDMS3N3**, as shown in Figures S14–S17 (Supporting Information). Then, they were reacted with **alkyne-MT** at the appearance of copper nanopowders to contribute to **AB** (Figures S18 and S19 in the Supporting Information), **AB2** (Figures S20 and S21 in the Supporting Information), and **AB3** (Figures S22 and S23 in the Supporting Information) through a click reaction. The predicted structures and compositions were consistent with the predicted NMR results and elemental analysis. The synthesized BCPs were further characterized by Fourier transform infrared spectroscopy (FT-IR) to confirm the removal of the precursors. In the FT-IR spectra of **AB**, **AB2**, and **AB3** with their homopolymers (Figures S24–S26 in the Supporting Information), **alkyne-MT** possesses a broadband at 3200–3550 cm^–1^ of O–H bonds, which also appeared in the BCPs studied. Additionally, the azide-terminated PDMS (**PDMS1N3**, **PDMS2N3**, and **PDMS3N3**) showed the characteristic peaks at 2110 cm^–1^ of azide stretching bonds, which disappeared after click reaction to ensure the removal of azide-terminated PDMS. In size exclusion chromatography (SEC), as shown in Figure S27 (Supporting Information), the polymer dispersity index (*Đ*_M_) of BCPs studied was in the range of 1.04–1.12, as summarized in Table S1 (Supporting Information). The SEC trace showed that the remaining precursors, alkyne-MT and azide-terminated PDMS, were both removed after a series of precipitations and dialysis. Although the initial *Đ*_M_ value of 1.28 for **PDMS–OH** commercial precursor indicates the presence of minor impurities, the series of reactions and purification steps reduces the dispersity to a narrow range of the BCPs’ *Đ*_M_. The residual impurity level in the resulting BCPs is negligible and does not influence the AFM results or the overall device performance.

### Thermal, Optical, and Electrochemical Properties of the Block Copolymers Studied

TGA (Figure S28, Supporting Information) and DSC (Figure S29, Supporting Information) were implemented to characterize the thermal properties of BCPs studied, providing the thermal degradation temperature at 5% weight loss (*T*_d_^5%^) and the glass transition temperature (*T*_g_), as summarized in Table S1 (Supporting Information). Based on TGA curves, the *T*_d_^5%^ values of all carbohydrate-based BCPs studied are close to 275 °C, implying their high thermal stability. The BCPs studied with more MT content degraded more easily under the heating process, consistent with previous research.^23^ In the DSC profiles, all BCPs studied presented prominent *T*_g_ values ranging from 100 to 140 °C, primarily influenced by the MT blocks. The higher *T*_g_ of the BCPs studied was attributed to the increased rigidity and abundance of hydroxy groups in MT, which are beneficial for tuning the self-assembly.

The BCP was blended with QDs at weight ratios of 9/1 to 7/3, spin-coated, and thermally annealed at 80 °C to form a nanocomposite film. The combinations of **AB**, **AB2**, and **AB3** with a QD are denoted as **ABQD**, **AB2QD**, and **AB3QD**, respectively. UV–vis absorption spectra (Figure 1b) and photoluminescence spectra (PL, Figures 1c and S30, Supporting Information) were conducted to discern the optical properties of the BCP/QD nanocomposites and the N-type semiconductors. According to the absorption profiles, the absorption peaks of QD, **PMDI**, **NDI**, and **PDI** were observed at wavelengths ranging from 200 to 450, 240 to 375, 275 to 435, and 380 to 700 nm, respectively. Next, the PL emission measurement was conducted with an excitation wavelength of 365 nm, matching their optical absorptions. The PL emissions are in the range of 470–555 nm. Among hybrid BCP/QDs composite films, **AB3QD** exhibited the highest emission peak, while **ABQD** presented the lowest emission peak, indicating that the more branching MT arms suggested a more suitable accommodation between BCP and QDs. Then, the frontier molecular orbital levels were extracted from cyclic voltammetry (CV) and the UV–vis absorption spectrum, as illustrated in Figure 1d. The UV–vis absorption onsets were calculated for the band gap (*E*_g_), where the λ_onset_ values of **NDI, PDI, PMDI**, and **QDs** were determined at 459, 620, 376, and 520 nm, respectively, corresponding to the *E*_g_ values of 2.7, 2.0, 3.3, and 2.4 eV, respectively. Next, CV profiles are depicted in Figure S31 (Supporting Information), and the HOMO/LUMO values of **NDI**, **PDI**, **PMDI**, and **QD** were calculated as −6.0/–3.3, −6.1/–4.1, −5.9/–2.6, and −5.9/–3.5 eV, respectively. To further confirm the energy level alignment presented in Figure 1d, the HOMO/LUMO values were compared with previous research that used ultraviolet photoelectron spectroscopy (UPS) and low-energy inverse photoemission spectroscopy (LEIPS) measurements for the rylenediimide derivatives and QDs. The literature values show good agreement with our measurements, with discrepancies of approximately 0.3 eV. Specifically, **PDI** exhibited an electron affinity (EA) of −6.6 eV, as determined by LEIPS, and an ionization energy (IE) of −4.0 eV, as extracted by UPS. These values are close to the HOMO/LUMO values of −6.1/–4.1 eV.^44,45^ Similarly, the literature indicates an **NDI**-based structure with the same central core but different side moieties, revealing HOMO/LUMO values of −6.1/–3.3 eV using UPS and UV measurements, which closely match the values herein reported as −6.0/–3.3 eV.^46^ In the case of **PMDI**, photoelectron spectroscopy reported HOMO/LUMO values of −6.0/–2.4 eV, consistent with those of −5.9/–2.6 eV.^47^ Also, reported QDs showed HOMO/LUMO values of −5.9/–3.5 eV, which are identical to those extracted herein of −5.9/–3.5 eV.^48^ The above results confirmed that the energy levels extracted from our electrochemical method are well aligned with the directly measured photoelectron spectroscopy data, strengthening the reliability of the energy level alignment herein. The energy levels can later be used to explain the compatibility of energy levels in charge transport and memory mechanisms.

### Characterization of Morphology and OFET Performance of the BCP Dielectrics

The morphology of the pure BCPs studied was characterized by AFM, as shown in Figures 2a–c and S32–S36 (Supporting Information), at 80 °C for 6–24 h. Figure S32 demonstrates the reliability and reproducibility of the AFM results under comparable thermal annealing conditions over five times. The **AB**, **AB2**, and **AB3** films were thermally annealed at 80 °C after 6 h, corresponding to their surface roughness (*R*_q_) of 5.23 ± 2.22, 2.00 ± 1.40, and 1.46 ± 0.33 nm, respectively. All of the low surface roughness implies high mobility in the following electrical performance. To optimize the surficial morphology, the AFM results of pure BCPs studied with extended annealing times of 12 and 24 h were obtained, as shown in Figures S33–36 (Supporting Information). In the small-scanning area, as shown in Figures S33 and S34, the AFM images of **AB** and **AB3** demonstrated the typical BCP morphology of horizontal lamellae and random spheres with the transient phase state of **AB2**. In the 10 × 10 μm^2^ scanning area, as shown in Figures S35 and S36 (Supporting Information), compared to **AB** showing the horizontal lamellar morphology, both **AB2** and **AB3** showed porous structures after thermal annealing. The aggregation of BCPs studied originated from the self-aggregation of MT, especially for **AB**, which forms a deep, circular plate on the surface. In contrast, as the more branched MT, a more porous surface was obtained. To determine the surficial components, contact angle measurements were performed three times using diiodomethane and ethylene glycol droplets. The resulting images are shown in Figure S37 (Supporting Information), and the corresponding surface energies are extracted in Table S2 (Supporting Information). According to the Owens–Wendt model, the surface energy of **AB** (40.7 mJ/m^2^) is much closer to that of oligosaccharides (50–60 mJ/m^2^), in contrast to the significantly lower value of PDMS (21 mJ/m^2^).^32,49^ The results imply that less MT occupied the surface for **AB2** and **AB3**, corresponding to the higher mobilities in the electrical performance of the OFETs. It is due to the rich hydroxy group on it that the MT-wetted surface traps the carriers, acting as an impedance for charge transport. These morphological differences are expected to influence the charge transport from OFET devices, as discussed below.

**Figure 2:**
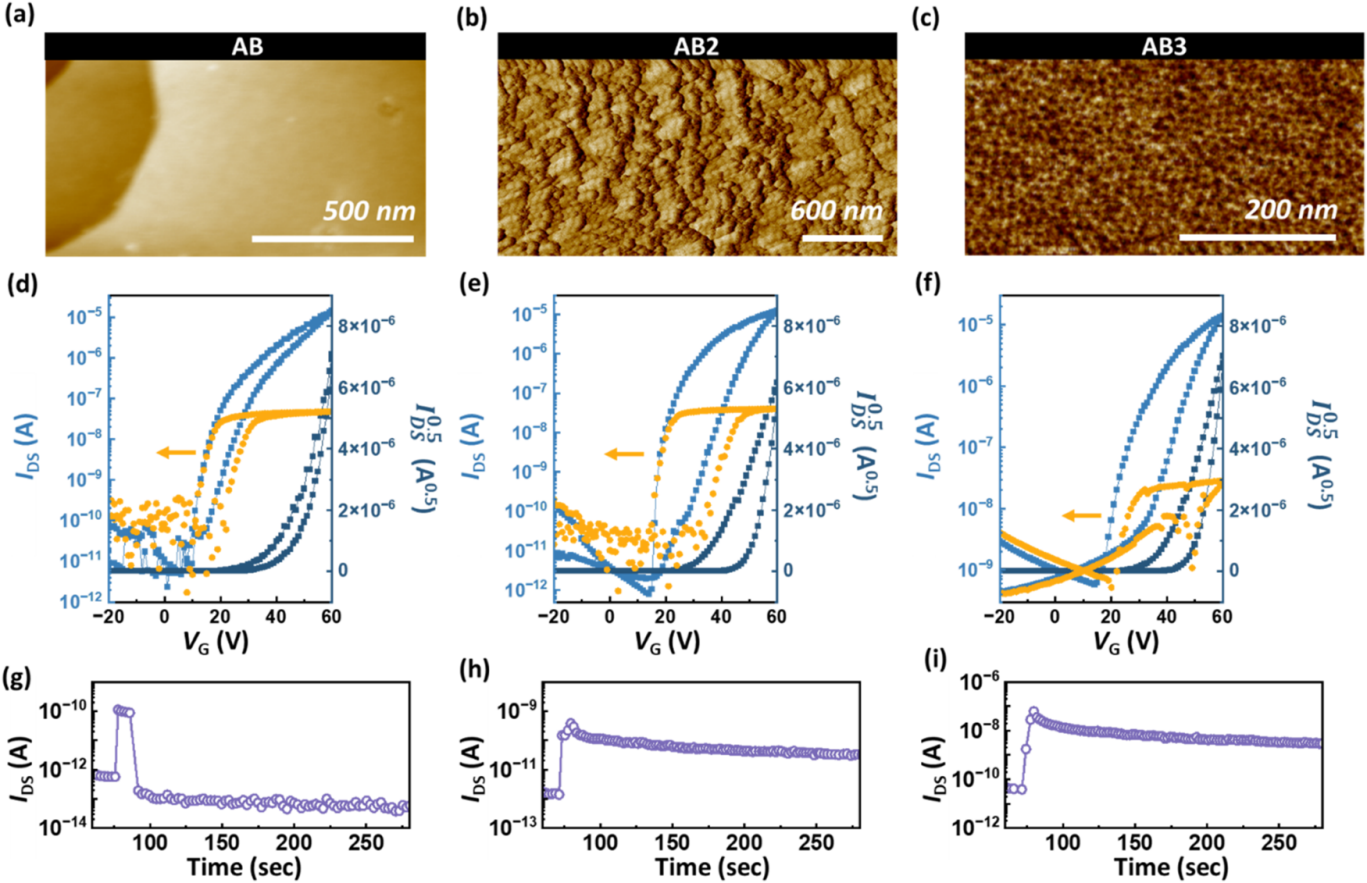
(a–c) AFM topographies, (d–f) dual-sweeping transfer curves, and (g–i) transient photocurrent curves of the phototransistor memory based on pure BCP electrets of (a, d, g) **AB**, (b, e, h) **AB2**, and (c, f, i) **AB3**. Note that in the transfer curves (d–f), the light-blue lines represent the drain current, the dark-blue lines represent the square-root drain current, and the yellow points indicate the gate current. The device was fabricated with an **NDI** channel, and the measurement was conducted at *V*_DS_ = 60 V, and 365 nm light illumination was applied for 30 s without gate bias in (g–i) to test the device’s photoresponse.

In addition to contact angle measurements and AFM morphological investigation, grazing small-angle X-ray scattering (GISAXS) analysis was performed to further investigate the nanostructure. Figure S38 (Supporting Information) depicts the 2D GISAXS patterns, and Figure S39 (Supporting Information) records the extracted 1D line-cutting plots. To quantize the structure parameters, the domain spacing (*d*) was calculated by Bragg’s equation: *d* = 2π/*q*, where *q* is the first scattering vector. In-plane directional analysis exhibited more significant characteristic peaks than out-of-plane directional analysis, which means that the pure BCPs exhibited stronger phase separation in in-plane directional analysis. Thus, from the in-plane directional analysis, the *d* values in the in-plane direction of **AB**, **AB2**, and **AB3** were 7.6, 8.4, and 8.3 nm, respectively. All of the BCPs feature sub-10 nm nanostructures, corresponding to the ordered nanopatterns formed through self-assembly phenomena after thermal annealing treatment. As can be seen, the smaller *q* values were obtained at a longer annealing time, indicating that the subnano-organization was easily controlled by the heating process. In the case of linear **AB**, the peaks located at *q*/q* = 1:√2:2 correspond to the sphere with a lamellar structure. Due to its low surface energy, PDMS preferentially migrated to the air–BCP interface, while the hydrophilic MT block wetted the ozone-treated SiO_2_ substrate. For both **AB2** and **AB3**, the *q*/q* = 1:√2:√3:2 values were observed, which corresponded to the random sphere. In the combination of the AFM morphology, the **AB** nanostructure can be realized as the horizontal lamellae self-assembled with the partial sphere. As the branched MT increased, the **AB2** morphology gradually transformed from the horizontal lamellar structure of **AB** to the spherical morphology of **AB3**. Combining the AFM roughness values, these domain sizes provide the quantitative results for correlating morphology with the electrical properties of OFETs.

The OFET device characteristics were investigated by spin-coating the BCPs on the SiO_2_/Si substrate, and NDI was thermally deposited onto the BCP dielectrics with a thickness of 50 nm serving as the channel. Figure 2d–f shows the OFET transfer curves, where the gate voltage (*V*_GS_) was dual-swept in the range from −20 to 60 V under a fixed voltage of 60 V, ensuring the typical performance for N-type OFET. Figure S40 depicts the transfer curves of pure BCPs in phototransistor memory devices during memory operation. The metal–insulator–metal (MIM) capacitor structure was measured at 1 kHz over nine different areas, and the resulting dielectric constant, thickness, and capacitance are summarized in Tables S3 and S4 (Supporting Information). The corrected mobilities are summarized in Table S5 (Supporting Information). Among the BCPs-based OFETs, **AB** exhibited the best performance in the BCP dielectrics with the highest electron mobility of 7.11 × 10^–2^ cm^2^ V^–1^ s^–1^ and a negligible hysteresis phenomenon, attributed to the suitable morphology with less charge trapping originating from the more PDMS-wetted surface. By correlating the AFM roughness and GISAXS domain sizes with OFET performance, we found that the **AB**-based OFET exhibited relatively higher roughness, showing the highest electron mobility due to its PDMS-wetted surface and reduced hydroxyl trapping. In contrast, although **AB2** and **AB3** thin films demonstrated a smoother surface and formed porous MT-rich morphologies with larger domain spacings, inducing more charge-trapping sites, this deteriorated carrier transport. These results confirm that the surficial morphology and nanostructure can influence the charge transport in these systems. Then, the transient curves were performed, as shown in Figure 2g–i, to confirm the photoresponse of OFET from the channel and its interplay with the BCP dielectric, providing the memory-test results summarized in Table S5 (Supporting Information). After using blue light of 365 nm to illuminate the device for 50 s, **AB** drastically decreased upon light removal. Although **AB2-** and **AB3**-based phototransistor memory achieved a marginal current ratio of *I*_ON/OFF_ > 10 after light removal, it did not meet the qualified standard for phototransistor memory (>10^3^). Moreover, the low photoresponse can be attributed to interfacial charge trapping, as all the neat BCPs exhibited comparable capacitance (Table S3in the Supporting Information). Therefore, the BCP will be blended with the QD to form a nanocomposite, serving as a floating gate dielectric in the phototransistor memory, to evaluate their photoresponse and charge-trapping capability further.

### Morphological Characterization of the BCP/QD Nanocomposite Films

After the hybrid BCP/QD composite films were spin-coated onto the device substrate, AFM was conducted to characterize the surface morphologies of the hybrid BCP/QDs, as shown in Figure 3a–c for the height and Figure S41 (Supporting Information) for the phase images. The *R*_q_ values of **ABQD**, **AB2QD**, and **AB3QD** were calculated at 5 different places of 17.87 ± 6.60, 9.72 ± 4.57, and 5.30 ± 2.04 nm, respectively. The higher branching MT contents in BCP/QDs corresponded to the more dispersed aggregation, implying better photoresponse and efficient charge transfer in device operations. Additionally, the *R*_q_ of all BCPs studied was flattened after the addition of QDs, suggesting that the role of QDs is in mitigating the incompatibility between hydrophilic and hydrophobic blocks. TEM was further applied to observe the in-depth morphology of BCP/QDs, as shown in Figure 3d–f. Fewer QD self-aggregations were observed from **AB3QD**, indicating that the underlying accommodation within the polymer layer is consistent with the AFM topography. Additionally, dynamic light scattering (DLS) revealed the tendency for QD aggregation, as shown in Figure S42. The results demonstrate that **AB** exhibits the broadest distribution, characterized by large aggregates, indicating the most severe aggregation. **AB2** shows an intermediate degree of aggregation with a slightly more confined distribution, suggesting a moderate dispersity. In contrast, **AB3** presents the narrowest profile with a largely suppressed large-size fraction, confirming the best dispersion among the BCPs studied with QDs. This aggregation trend of BCP blended with QDs is consistent with the AFM phase images and the corresponding device performances, strengthening the reproducibility and reliability of the results.

**Figure 3:**
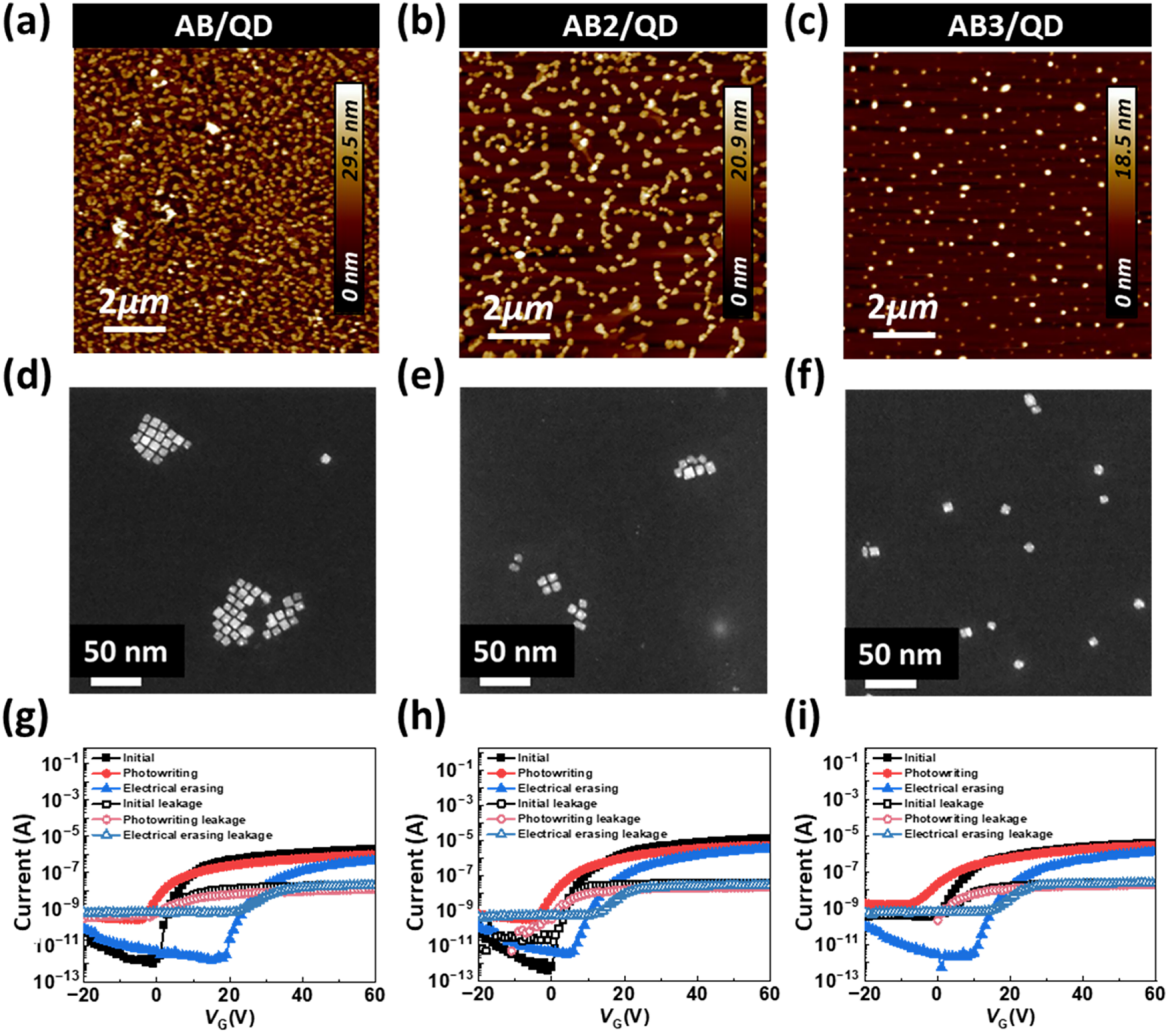
(a–c) AFM topographies, (d–f) TEM images, and (g–i) transfer characteristics of the phototransistor memory based on (a, d, g) **ABQD**, (b, e, h) **AB2QD**, and (c, f, i) **AB3QD** nanocomposite films as a floating gate dielectric (BCP/QD = 8:2). Device with an **NDI** channel was measured at *V*_DS_ = 60 V, with photowriting under 365 nm light for 60 s and electrical erasing at *V*_GS_ = 60 V for 1 s. In (g–i), the solid symbols indicated the drain current (*I*_DS_), while the hollow symbols presented the corresponding gate current (*I*_G_) as the leakage currents.

Grazing incidence wide-angle (GIWAXS) and GISAXS analyses were performed to explore the film structures further. Figure S43 depicts the 2D GIWAXS patterns of the pristine and thermally annealed BCP/QD films, and the 1D GIWAXS line-cutting profiles are demonstrated in Figure S44 (Supporting Information). As can be seen, the QD signals were picked at *q* = 11.0 and 14.6 nm^–1^, corresponding to the typical CsPbBr_3_ diffraction signals of (110) and (200), respectively.^16^ The pristine and annealed BCP/QDs thin film states provided comparable QD signals after thermal annealing, implying that this annealing process did not interfere with the QD structure. Then, GISAXS analysis was conducted for the self-assembled structures of the BCP/QDs. The 2D GISAXS patterns are recorded in Figure S45 (Supporting Information), and the extracted 1D line-cutting plots are depicted in Figure S46 (Supporting Information). According to Bragg’s equation, the *d* values of **ABQD**, **AB2QD**, and **AB3QD** were 11.0, 10.7, and 10.3 nm, respectively, and after the thermal annealing treatment, the *d* values of **ABQD**, **AB2QD**, and **AB3QD** were 10.7, 10.7, and 10.3 nm, respectively. Notably, the branching architectures of both **AB2QD** and **AB3QD** were independent of the thermal annealing compared to the linear **ABQD** composite film. The results indicated that the BCP nanostructures become rigid and stable, allowing them to accommodate QDs.

### Phototransistor Memory Characterization of the BCP/QD Floating Gates

After the morphology was characterized, the phototransistor memory based on BCP/QDs with **NDI** was fabricated and characterized. To optimize the performance of the best electrical devices, the transfer and transient curves of the BCP/QDs at different ratios in the phototransistor memory are shown in Figures 3g–i, S47, and S48 (Supporting Information), with BCP/QD ratios of 7:3, 8:2, or 9:1. Note that solid symbols indicate the drain current (*I*_DS_) and hollow symbols present the corresponding gate current (*I*_G_) as the leakage currents. In Figure 3g–i, for the initial operation, the gate voltages were swept from −20 to 60 V at the fixed drain voltage of 60 V, with the black “initial” line. Then, after illuminating the phototransistor memory devices with 365 nm blue light for 30 s, the read-out transfer curve shifted to a more negative electrical *V*_GS_ region, attributed to charge trapping in the floating gate materials. The red read-out line was defined as the ON state after the photowriting program. The negative electrical impulse of *V*_GS_ = −60 V drove the transfer curves to detach the charges. The blue read-out line was the “OFF” state upon the electrical erasing program. Obviously, at the extremely high bias *V*_G_ = 60 V, the leakage current (*I*_G_) remains around 10^–8^ A, which is 2 orders of magnitude less than the *I*_DS_. In combination with the morphological results, this confirmed that although the more branching MT of BCP exhibited the increased nanoporous structures, the devices were operated according to the standard OFET operation. The resulting μ, memory window (Δ*V*_th_), and *I*_on/off_ values of phototransistor memory are extracted to Tables S6–S8 (Supporting Information) with BCP/QD ratios of 9:1 to 7:3. When the different ratios of BCP and QDs were compared, the best phototransistor memory for BCP/QDs was observed at a ratio of 8:2. In comparing the different MT arms in BCP/QDs influencing the performance of the phototransistor memory, **AB3QD** outperformed the highest Δ*V*_th_ of 15.5 V and *I*_ON/OFF_ of 3.09 × 10^5^ compared to the *I*_ON/OFF_ of 5.06 × 10^4^ and 1.33 × 10^5^ for **ABQD** and **AB2QD**, respectively. Notably, the higher mobility of **AB3QD** can be elucidated by the relatively lower roughness of the hybrid composite film. To strengthen the roughness–performance correlations, the **ABQD** device with a higher *R*_q_ value of 28.73 ± 4.49 nm was prepared, and its memory data are shown in Figure S49 (Supporting Information), where the resulting mobility and *I*_ON/OFF_ are 3.20 × 10^–4^ cm^2^ V^–1^ s^–1^ and 1.57 × 10^3^, respectively. Thus, while smoother morphology may partially facilitate carrier transport, the comparison clearly demonstrates that the larger *R*_q_ values worsen the device performance. As can be seen, the increasing branching of MT in BCP/QD led to superior memory performance in optoelectronic devices, which is attributed to the more suitable dispersion and accommodation of QDs.

To tune the compatibility between the channel and the floating gate layer, a series of rylenediimide-based N-type semiconductors were deposited on the **AB3QD** phototransistor memory. The AFM height and phase images of **PMDI**, **NDI**, and **PDI** are shown in Figures 4a–c and S50 (Supporting Information), corresponding to the *R*_q_ values of 10.41 ± 3.59, 7.32 ± 2.11, and 11.65 ± 4.77 nm, respectively. **PMDI** and **PDI** formed a sphere-like nanostructure with a relatively rougher surface. In contrast, **NDI** exhibited the smoothest nanofibrillar morphology, which contributed to a superior electrical and memory performance. After optimization of the floating gate dielectric, the transfer curves based on different N-type channels are recorded in Figure 4d–f. The detailed device characteristics and parameters are summarized in Figure S51 and Table S9–11 (Supporting Information) for **PDI**-based devices and in Figure S52 and Tables S12–S14 (Supporting Information) for **PMDI**-based devices with BCP/QD ratios of 9:1 to 7:3. Figure S53 presents the device’s transient characteristics of light illumination. When comparing the different channel materials, the moderate performance of **PDI**-based memory (Δ*V*_th_ of 6.8 V and *I*_ON/OFF_ of 4.48 × 10^4^) originated from the underlying interference from the floating gate dielectric, which will be further discussed in the later section. In contrast, the **PMDI**-based memory exhibited the lowest performance (Δ*V*_th_ of 26.2 V and negligible *I*_ON/OFF_) due to its relatively higher roughness and poor semiconductivity, which originated from its poor conjugation. To further clarify the mechanism of wavelength-dependent behavior, Figure S54 (Supporting Information) presents a comparison of **PDI**- and **NDI**-based devices under 365 and 450 nm illumination. With 450 nm illumination, **NDI** absorption was minimized, while QDs remained the primary absorber in the **NDI**-based memory device, resulting in a low *I*_ON/OFF_ value of 1 × 10^2^ after light illumination. However, the **PDI**-based memory device exhibited a higher *I*_ON/OFF_ value of 4 × 10^2^ than that of **NDI**, attributing to the **PDI** still absorbing most of the light with the addition of the **QDs**. In contrast, under 365 nm light illumination, **NDI** exhibits strong absorption, according to the UV profiles, and therefore shows a higher *I*_ON/OFF_ value over 3 × 10^5^ than that from **PDI** (7 × 10^3^), suggesting that the channel dominates the photoexcitation at this wavelength. This comparison highlights that the advantage of **NDI** at 365 nm arises primarily from its strong UV absorption, whereas the contribution from the channel was significantly minimized with a longer light wavelength of 450 nm. The superior memory performance based on **PDI** reflects its more suitable energy level alignment and interfacial charge trapping processes. Therefore, the wavelength-dependent behavior can be rationalized by (i) the absorption of QDs at 450 nm and (ii) the absorption of different channel layers. In addition, as supported by the TRPL and PL quenching analyses discussed later, the interfacial charge transfer efficiency (CTE) also contributes to this behavior.

**Figure 4:**
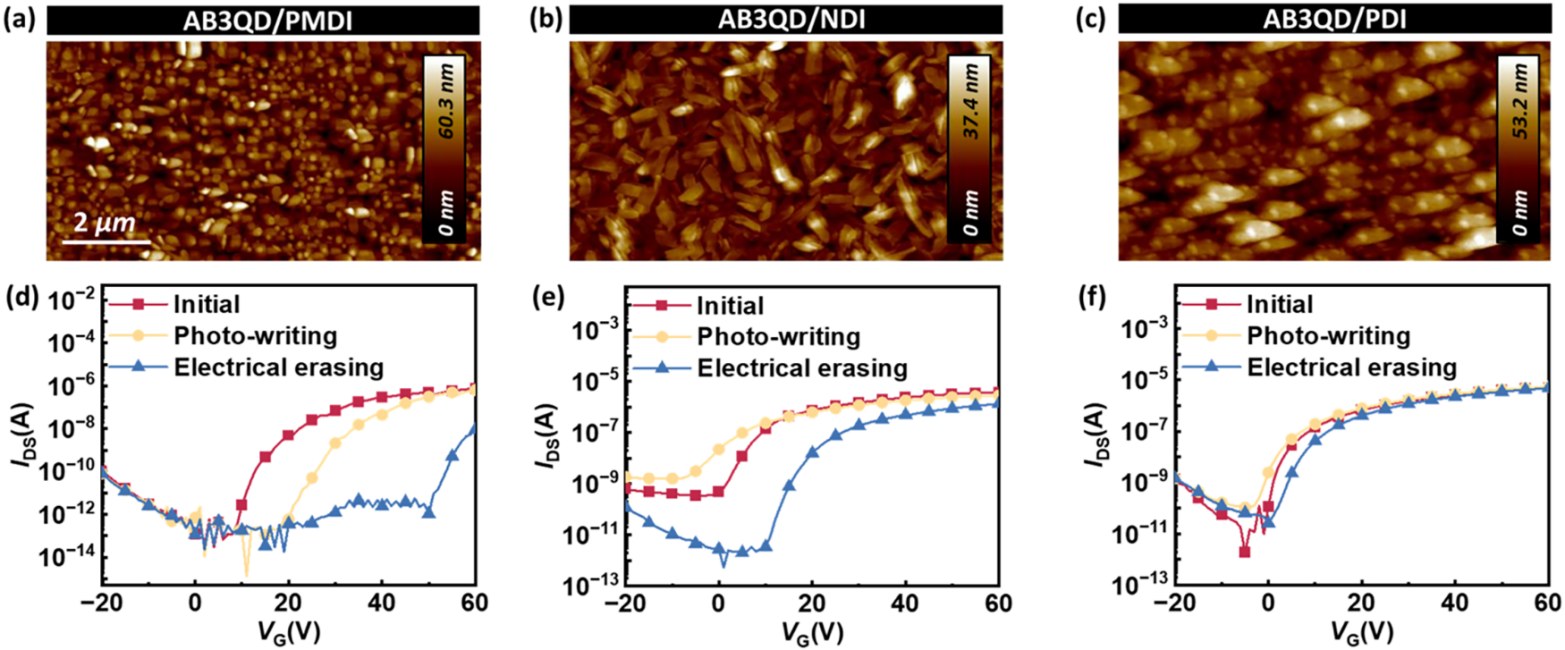
(a–c) AFM topographies and (d–f) transfer characteristics of the phototransistor memory based on (a,d) **PMDI**, (b, e) **NDI**, and (c, f) **PDI** channels. Note that the floating gate dielectric was **AB3QD** (BCP/QD = 8:2) and the device measurement was conducted at *V*_DS_ = 60 V. The photowriting was conducted by illuminating a 365 nm light for 60 s, and the electrical erasing was applied with *V*_G_ = −60 V for 1 s.

### Photodynamic Characterization of the BCP/QD Nanocomposite Films

The photodynamic properties of the BCP/QD nanocomposite films were investigated to reveal the memory performance further. Therefore, the time-resolved photoluminescence (TRPL) and steady-state PL emission measurements were conducted to obtain the exciton lifetimes, CTE, PL quenching ratios, and photoluminescence quantum yield (PLQY) of the BCP/QD films and bilayered films stacked with the N-type semiconductors. Figures 5a and S55a–57a (Supporting Information) record the 1D TRPL profiles, and Figures 5b and S55b–57b (Supporting Information) depict the 2D extracted curves with their fitting lines for the BCP/QD films, bilayered films stacked with **NDI**, **PMDI**, and **PDI**, respectively. The 1D TRPL fitting lines were calculated with a biexponential decay function with eq 1, and the resulting average lifetime (τ_avg_) was calculated by eq 2

1 $$I\left(t\right)=A_{1}\exp\left(\frac{-t}{\tau_{1}}\right)+A_{2}\exp\left(\frac{-t}{\tau_{2}}\right)$$

2 $$\tau_{\mathrm{avg}}=\frac{{\sum_{i=1}^{n}A_{i}\left(\tau_{i}\right)}^{2}}{\sum_{i=1}^{n}A_{i}\tau_{i}}$$

where τ_1_ and τ_2_ represent the short- and long-lived exciton lifetimes, respectively, and *A*_1_ and *A*_2_ are correlated to the fractions. The resulting values are listed in Tables S15–S18 (Supporting Information) for the monolayered hybrid BCP/QDs composite film and bilayered films with **NDI**, **PMDI**, and **PDI**, respectively. Note that the instrument response function (IRF) of the streak camera system in TRPL is 82 ps with a 375 nm calibrated laser.^50,51^ Since all extracted lifetimes are in the nanosecond scale, the system time resolution is sufficient to distinguish the observed time differences, where the results are well above the IRF width. Moreover, all TRPL decays were fitted using a biexponential reconvolution model, reduced chi-square (χ^2^) values on the order of 10^–4^, and coefficients of determination (*R* ^2^) of 0.99–0.999, indicating good agreement between the experimental data and the fitting function with negligible errors. These results confirm that the fitting errors and the IRF do not significantly influence the resulting lifetimes, and therefore, the derived CTE and *k*_CT_ values remain reliable. Among the BCP/QDs, the **AB3QD** layer possesses the highest τ_avg_, implying successful passivation and accommodation of the QDs in the polymer matrix. The more branching MT in BCP can mitigate the QD aggregation, sufficiently improving the photoresponse and charge trapping in phototransistor memory. The underlying mechanism can be elucidated by correlating surface energy with the wetting coefficient (ω) calculation and the domain spacing in GISAXS results.^52^ With the Owens–Wendt method, the reported surface energies of QD, PDMS, and oligosaccharide are 32, 21, and ∼55 mJ/m^2^, respectively, resulting in the calculated interfacial tensions of σ_QD–PDMS_ = 5.9, σ_QD–MT_ = 17.7, and σ_PDMS–MT_ = 42.6 mJ/m^2^, respectively.^32,49,53^ Accordingly, the wetting coefficient (ω) can be calculated using the equation $\omega =\frac{\sigma_{p‐B}-\sigma_{p‐A}}{\sigma_{A‐B}}$ , where σ_p‑A_ and σ_p‑B_ are the interfacial tensions between the particle (QD ligand shell) and phases A (PDMS) and B (MT), respectively, and σ_A–B_ is the interfacial tension between PDMS and MT domains. Therefore, the calculated ω value of 0.28 suggests that QDs preferentially reside at the PDMS/MT interfaces on the PDMS side. Therefore, introducing more branched MT enlarges the interfacial area and subdivides PDMS domains, which suppresses QD aggregation. The tendency is consistent with the GISAXS results, which show that *d*-spacings expanded beyond 10 nm, leading to improved photoresponse and charge-trapping properties. To further quantify the charge transfer process, except for **PDI**-based bilayer composite films, the charge transfer efficiency (CTE) and charge transfer rate (*k*_CT_) were calculated using the following equations

3 $$\mathrm{CTE}\;\left(\%\right)=\frac{\tau_{\mathrm{bilayer}}-\tau_{\mathrm{BCP}/\mathrm{QDs}}}{\tau_{\mathrm{BCP}/\mathrm{QDs}}}\times 100\%$$

4 $$\frac{1}{\tau_{\mathrm{bilayer}}}=\frac{1}{\tau_{\mathrm{BCP}/\mathrm{QDs}}}-\frac{1}{\tau_{\mathrm{CT}}}$$

where 1/τ_CT_ = *k*_CT_ (ns^–1^). The **PDI**-based was calculated using the following equation, with the assumption of the charge transfer from the **PDI**, contributing to the emission light of QDs absorbed by **PDI**.

5 $$\mathrm{CTE}\;\left(\%\right)=\frac{\tau_{\mathrm{bilayer}}-\tau_{\mathrm{PDI}}}{\tau_{\mathrm{PDI}}}\times 100\%$$

6 $$\frac{1}{\tau_{\mathrm{bilayer}}}=\frac{1}{\tau_{\mathrm{PDI}}}-\frac{1}{\tau_{\mathrm{CT}}}$$

When compared to the different N-type semiconductors, the **NDI**- and **AB3QD**-based bilayers exhibited the highest CTE (94.2%) and *k*_CT_ (6.87 ns^–1^) values, implying that the aggregated QDs dispersed in the BCP matrix facilitated charge trapping. The tendency from charge transfer efficiency and rate proved that the aggregated QDs in a more branching MT architecture demonstrate a better charge transfer process, indicating superior memory performance in optoelectronic devices.

**Figure 5:**
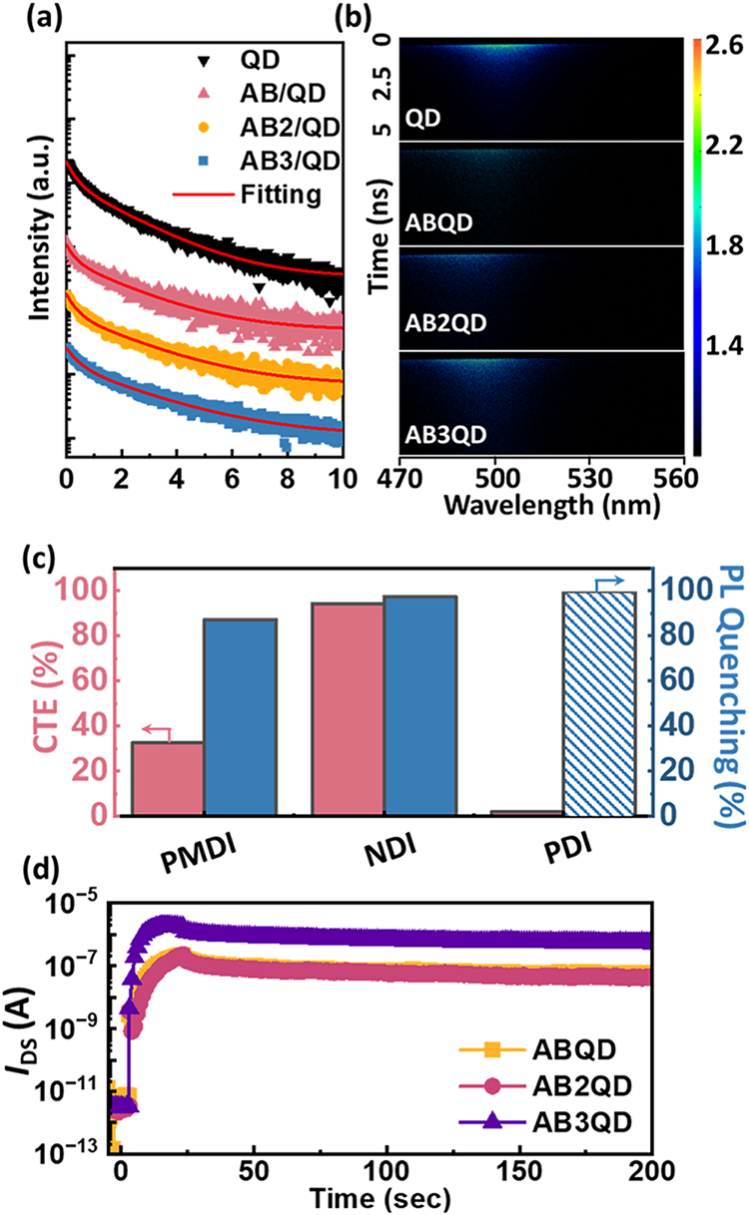
(a) TRPL 1D decay profiles and (b) 2D contour patterns of pure QD and BCP/QD (8/2) nanocomposite films with an excitation wavelength of 375 nm. Note that the fitting calculation used a biexponential reconvolution model, with reduced chi-square (χ^2^) values of 1.2–1.5 × 10^–4^ and coefficients of determination (*R* ^2^) of 0.99–0.999. (c) PL parameters of the **AB3QD** films stacked with the N-type channel layer. Note that the parameters of **PDI** were calculated based on an excitation wavelength of 630 nm due to its substantial light absorption. (d) Transient photocurrent curves of the phototransistor memory based on the BCP/QD nanocomposite films as a floating gate and the **NDI** channel. Note that the device was fabricated with an **NDI** channel, and the measurement was conducted at *V*_DS_ = 60 V, and 365 nm light illumination was applied for 60 s to test the device’s photoresponse.

As seen in Figure 1b, the PL quenching ratios of the **AB**, **AB2**, and **AB3** bilayers with **NDI** are 90.4, 90.6, and 97.5%, respectively. When comparing the different BCP architectures, a similar tendency was observed, where the more branching MT in BCPs corresponds to higher charge transfer efficiency and rate, as shown in Figure S30a,b for the PDI and PMDI-based bilayered films. To compare the different N-type semiconductors based on **AB3QD**, the PL quenching ratios of **AB3QD/PDI** and **AB3QD/PMDI** are 99.0 and 87.1%, respectively. However, **AB3QD**/**NDI** possesses comparable PL quenching and a higher CTE than **PDI**, as shown in Figure 5c. This improvement explains the device’s high performance, utilizing **AB3QD** as a floating gate dielectric and **NDI** as the channel.

### Temporal Response of Phototransistor Memory with the BCP/QD Floating Gates

As shown in Figure 5d, the transient photoresponse of **AB3QD** outperforms those of **ABQD** and **AB2QD**. Therefore, to further explore the temporal photoresponse based on **AB3QD** as a floating gate memory layer with an **NDI**-based transporting layer, Figure 6 demonstrates that the different operational parameters were optimized to tune the memory behavior. Figure 6a shows that the phototransistor memory was exposed to various light-illuminating times, resulting in an improvement in *I*_ON/OFF_ > 10^5^ after extending the light-illuminating time from 0.1 to 30 s, which highlights the contribution of the floating gate photoresponsivity to the memory devices. Then, the *V*_DS_ was tuned from 0.1 to 60 V to minimize the reading power, as recorded in Figure 6b. As the *V*_DS_ increased, the memory behavior improved to *I*_ON/OFF_ > 10^5^, indicating a distinguishable memory ratio and good nonvolatile behavior. In Figure 6c, the transient curves with different light wavelengths of 365, 405, and 450 nm correspond to the *I*_ON/OFF_ values of 3 × 10^5^, 9 × 10^4^, and 1 × 10^2^, respectively, implying the best photoresponse under 365 nm light illumination. To verify the memory switchability, write–read–erase–read (WRER) measurements were conducted over 10 cycles, resulting in bistable and distinguishable ON and OFF states with *I*_ON/OFF_ values exceeding 1 × 10^5^. To characterize the memory stability, Figure 6e shows a long-term retention measurement. The high ON state current value was maintained at 10^–6^ A, and the low OFF state was located at <10^–12^ A, corresponding to an *I*_ON/OFF_ ratio of around 10^6^ over 10,000 s, implying excellent memory stability and photoresponse.

**Figure 6:**
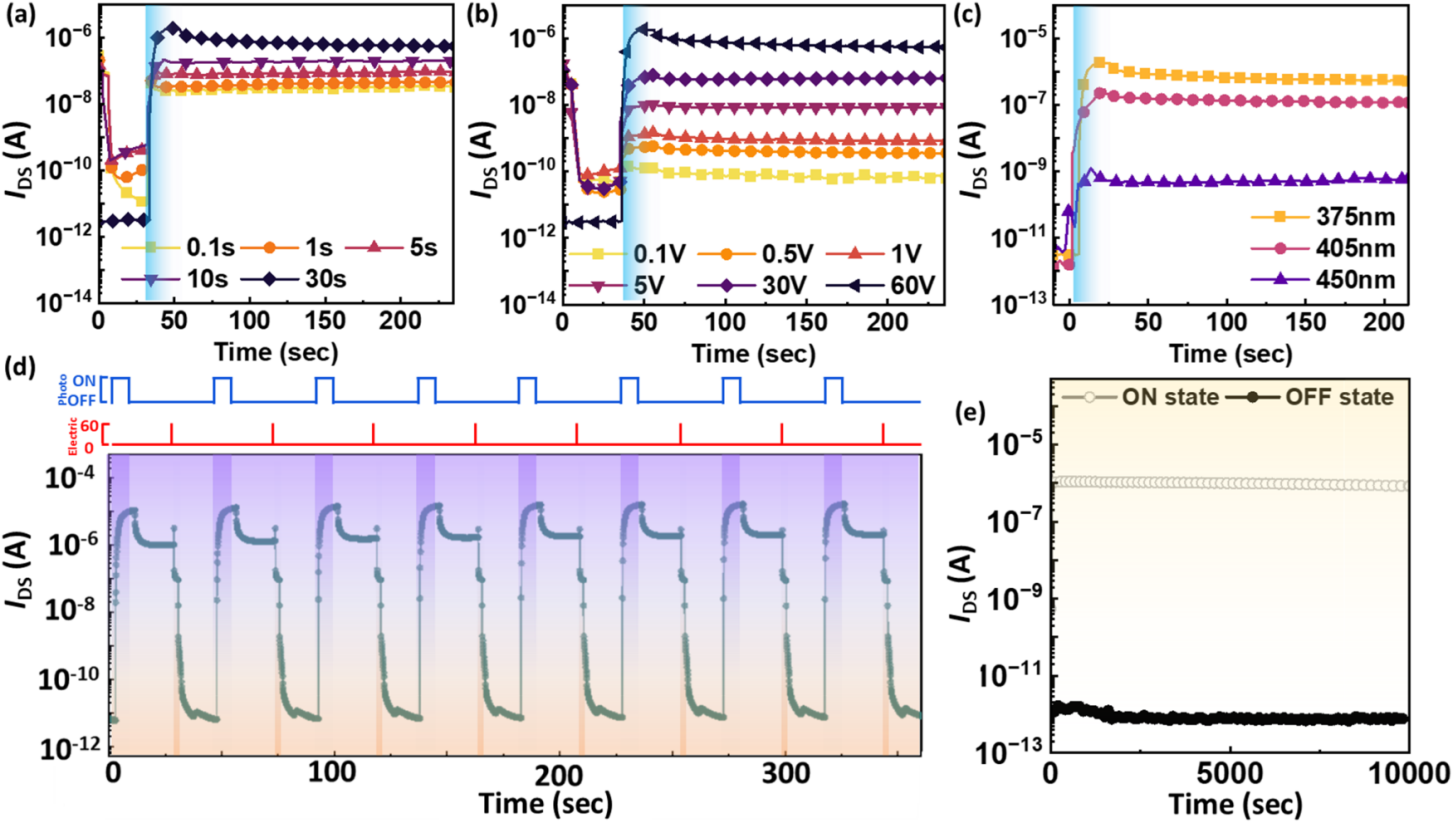
Phototransistor memory characteristics based on the floating gate of **AB3QD** (BCP/QD = 8:2) and the channel of **NDI**: Device parameter optimizations were performed with (a) different times of light programming, (b) *V*_DS_, and (c) wavelengths of light illumination. Note that the blue-shaded areas are denoted as the light illumination. (d) WRER cycles of the phototransistor memory device. Note that the red-shaded area means the bias of *V*_GS_ = −60 V for 1 s (OFF state), and the blue-shaded area indicates 365 nm blue light illumination for 10 s (ON state). (e) Long-term retention of the phototransistor memory device at *V*_DS_ = 60 V over 10,000 s.

Figure 7 illustrates the mechanism of the QD dispersion process and its behavior during the memory operation of the phototransistor in this study. Compared to the BCP architectures, Figure 7a explains that the linear AB architecture allocates the QDs in the layered matrix; however, the poorly dispersed QDs cannot provide an excellent performance for the phototransistor memory. By introducing branching MT arms in BCP, better QD accommodations were observed in the phase-segregated matrix, leading to improved performance for memory devices. Specifically, the most substantial QD embedding was obtained from the **AB3QD** nanocomposite film. In addition, Figure 7b–d elucidates the charge transfer between the transporting and floating gate memory layers after QDs absorbed light. As shown in Figure 7b, due to the comparable LUMO level of **NDI**, the photogenerated electrons could be easily transferred to the semiconducting layer during the photowriting process. In contrast, the photogenerated holes were blocked in the memory layer due to the lower-lying HOMO levels of **NDI**. Therefore, the high read-out current and superior current stability were observed in the transient curves, implying that electrons were directed to the **NDI** and holes were trapped in the floating gate memory layer. Then, the positive gate voltage was applied to remove the excess charges through electrical erasing programming. **NDI** disclosed that the light illumination successfully induced the charges and trapped them based on the energy level alignments, indicating that **NDI** can be regarded as a suitable channel layer for the phototransistor memory in this study. In the case of **PDI**, as presented in Figure 7c, the low-lying LUMO level of **PDI** in comparison to that of the QD creates an unfavorable energy level alignment for electron transfer, causing the photogenerated excitons to undergo direct recombination shortly after generation in the QDs. Subsequently, **PDI** was excited indirectly by absorbing the emission from the QD, generating excitons to enhance the electron transport in the channel layer. Since **PDI** possesses a lower-lying HOMO level, the photogenerated holes are driven back toward the memory layer. At the same time, the electrons are retained within the channel layer, resulting in stable read-out data after light illumination. In Figure 7d, as with **PMDI**, electrons were unable to transfer effectively to the semiconducting layer under lighting due to the unfavorable, high-lying LUMO levels of **PMDI**, resulting in no observable photoresponse in the device characteristics.

**Figure 7:**
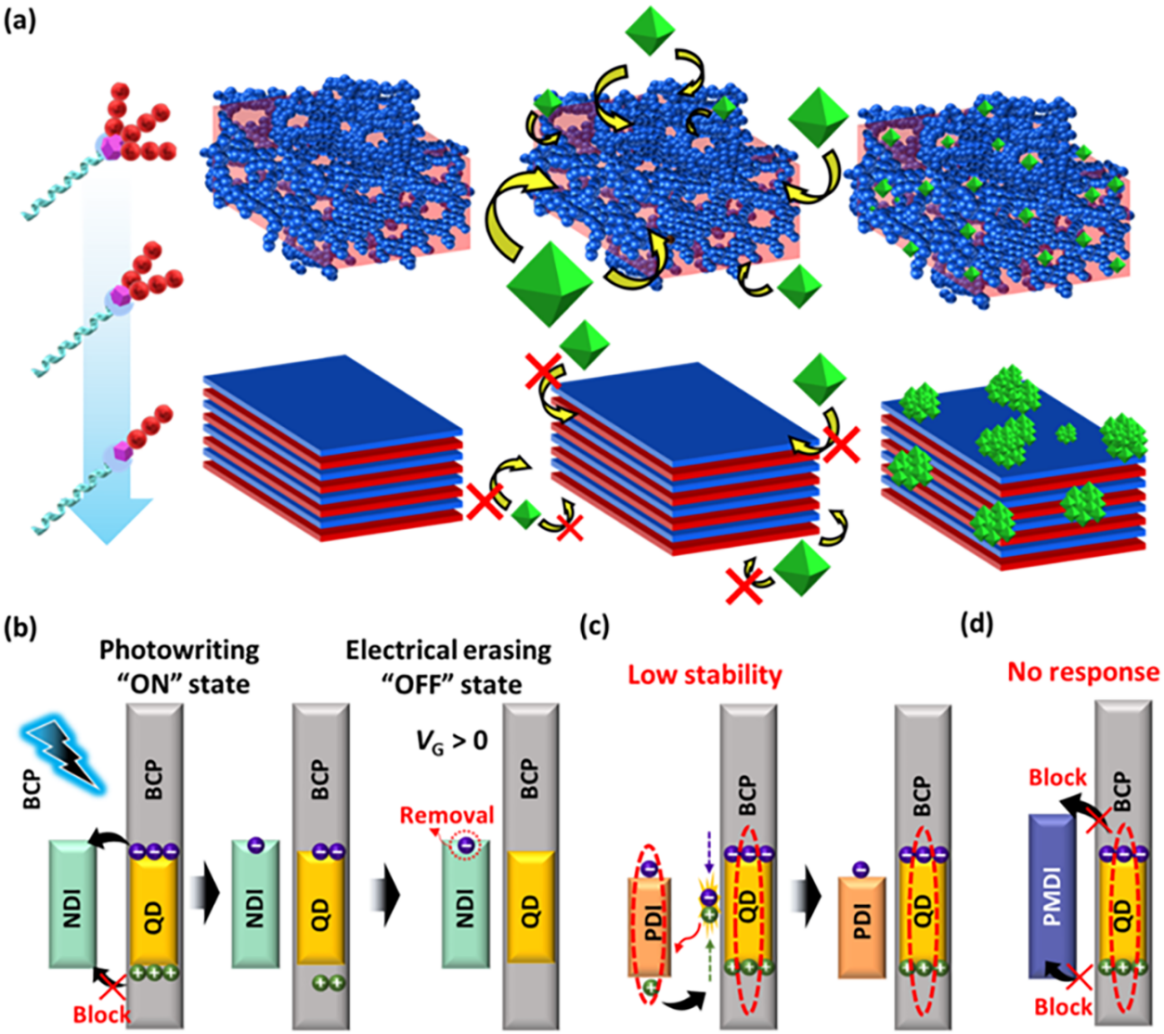
(a) Illustration of the relationship between the BCP architectures and QD accommodation: the branched architecture induces a porous structure to improve the dispersion of QDs in the floating gate layer. Working mechanism and energy level alignments of the BCP/QD floating gate and the N-type channel including (b) **NDI**, (c) **PDI**, and (d) **PMDI**: the optimized HOMO/LUMO levels of the N-type channel can improve its electron transfer and hole blocking beneath the floating gate during photowriting.

## Conclusions

In summary, this is the first investigation into the impact of BCP architectures with different numbers of branching arms on the floating gate application in phototransistor memory. Additionally, the compatibility between the N-type channel and floating gate layers was confirmed to enhance the performance of the phototransistor memory. Among the BCPs, **AB3** enhanced QD accommodations in the polymer matrix, while **NDI** exhibited the best energy level alignments with QDs. With **NDI** as the channel, **AB3QD** showed the highest *I*_ON/OFF_ ratio of 3.09 × 10^5^ compared to **ABQD** (5.06 × 10^4)^ and **AB2QD** (1.33 × 10^5^). With **AB3QD** as the floating gate dielectric, **PDI** exhibited a lower *I*_ON/OFF_ ratio of 4.48 × 10^4^, and PMDI was unable to produce a photoresponse. Due to the comparable LUMO and low-lying HOMO energy levels of **NDI**, the **NDI**-based memory devices exhibit good charge transfer, followed by stable charge trapping. Additionally, the reference devices, comprising pure BCP dielectrics and an **NDI** channel, lack a photoresponse and memory capability. Therefore, the excellent results from this combination were achieved by utilizing a well-accommodated QD with the smoothest roughness, suitable energy level alignments between the channel and floating gate, and a high charge transfer efficiency. Hence, it rendered the highest performance, indicating the well-defined memory performance among the BCP/QD floating gates for phototransistor memory devices. Moreover, it presented long-term retention (*I*_ON/OFF_ > 10^6^ over 10,000 s) and cyclic WRER endurance stability (*I*_ON/OFF_ > 10^5^) over 10 cycles, indicating its outstanding data discernibility, conspicuous digital storage, and exceptional switching performance under light illumination and orthogonal electrical programming.

## Supplementary Material



## Data Availability

Data will be made available on request.

## Notes

^1^H and ^13^C NMR spectra and synthetic procedures of the monomers and polymers; TGA and DSC characterization of the polymers; GIWAXS and GISAXS characteristics of the BCP/QD nanocomposites films; phototransistor memory device; and TRPL fitting parameters of the BCP/QD nanocomposites and floating gates (PDF)
