# Supplementary material for: Oligosaccharide Block Copolymers with Branched Architectures and Channel Energy Level Optimizations for High-Performance Floating Gate Phototransistor Memory
Source: ACS Appl Mater Interfaces. 2025 Sep 15;17(38):53837–51. doi: 10.1021/acsami.5c13139 (PMC12464903; doi:10.1021/acsami.5c13139)
Supplement: Supplementary file 1 [file am5c13139_si_001.pdf]

Supporting Information

Oligosaccharide Block Copolymers with Branched Architectures and Channel Energy Level Optimizations for High-Performance Floating Gate Phototransistor Memory

Ping-Jui Yu,[a,b] Wei-Cheng Chen,[a] Ya-Shuan Wu,[a] Bi-Hsuan Lin,[c] Yan-Cheng Lin,[d,e] Redouane Borsali,[b*] and Wen-Chang Chen[a,e*]*

[a] Department of Chemical Engineering, National Taiwan University, Taipei 10617, Taiwan

[b] University of Grenoble Alpes, CERMAV-CNRS 38000 Grenoble, France

[c] National Synchrotron Radiation Research Center, Hsinchu 300092, Taiwan

[d] Department of Chemical Engineering, National Cheng Kung University, Tainan 70101, Taiwan

[e] Advanced Research Center for Green Materials Science and Technology, National Taiwan University, Taipei 10617, Taiwan

*Corresponding author. E-mail: ycl@gs.ncku.edu.tw (Y.-C. L.); borsali@cermav.cnrs.fr (R. B.); chenwc@ntu.edu.tw (W.-C. C.)

Table S1. Molecular weight, volume fraction, and thermal properties of the polysaccharide-based BCPs studied.

Polymer	f_A	M_n^a	$Đ^a$	$T_d^{5\% b}$ (°C)	T_g^c (°C)
**AB**	0.77	1940	1.12	274.8	104.3
**AB2**	0.63	2540	1.06	279.7	137.4
**AB3**	0.53	3110	1.04	276.2	140.3

[a] Calculated from the DMF SEC at 40 °C; [b] Determined the degradation temperature of 5% weight loss from the TGA heating from 100 to 600 °C at a heating rate of 10 °C min^{-1} under an air atmosphere; [c] Extracted from the DSC heating from –80 to 240 °C at a heating rate of 10 °C min^{-1} under a nitrogen flow.

Table S2. The surface energies of the BCPs derived from the contact angle measurements based on the Owens-Wendt model.

	**Contact angle (°)**		**Surface energy (mJ/m²)**		
	**Diiodomethane**	**Ethylene glycol**	σ_s^p	σ_s^d	σ_s
**AB**	69.60	32.16	17.6	23.1	40.7
**AB2**	72.80	27.14	21.8	21.3	43.1
**AB3**	59.60	24.92	14.7	28.8	43.5

Table S3. Dielectric properties of pure BCPs based on a metal-insulator-metal (MIM) structure.

	**AB**	**AB2**	**AB3**
D_k^a	3.0	3.1	3.4
Thickness (nm)[b]	73.4	82.5	70.2
C^c (nF cm^{-2})	17.6	15.1	19.2

[a] Dielectric constant is extracted at 1 kHz over nine different areas, where the bottom-layer metal is silicon, the middle layer is BCPs studied, and the top-layer metal is gold; b thickness is determined with an optical thickness meter over five times. [c] The capacitance is obtained with the dielectric constant and thickness.

Table S4. Dielectric properties of BCP/QD at different ratios based on an MIM structure.

	**AB**	**AB2**	**AB3**	**AB**	**AB2**	**AB3**	**AB**	**AB2**	**AB3**
		**9/1**			**8/2**			**7/3**	
D_k	3.2	3.3	3.4	3.4	3.5	3.7	3.6	3.7	3.8
Thickness (nm)	43.2	52.6	43.2	61.6	66.7	63.0	88.2	53.0	44.2
C (nF cm^{-2})	22.7	21.4	23.1	20.2	19.8	20.7	17.6	22.2	23.8

Table S5. Memory performance of phototransistor memory derived from the transfer curves for pristine BCPs with **NDI** as the active layer under a fixed V_{DS} = 60 V.

	**AB**	**AB2**	**AB3**
μ (cm^2 V^{-1} s^{-1})[a]	7.11×10^{-2}	2.15×10^{-2}	3.10×10^{-2}
$V_{th, write}$ (V)	22.6	5.6	7.5
$V_{th, erase}$ (V)	30.1	17.4	24.6
Δ V_{th} (V)	7.5	11.8	17.1
I_{on} (A)	1.00 × 10^{-13}	3.31 × 10^{-11}	2.92 × 10^{-9}
I_{off} (A)	5.70 × 10^{-13}	1.42× 10^{-12}	3.85 × 10^{-11}
$I_{on/off}$	--	2.33 × 10^{1}	1.31 × 10^{2}

[a] Carrier mobility corrected with each capacitance.

Table S6. Phototransistor memory device parameters derived from the transfer curves for BCPs/QDs at a ratio of 9/1 with **NDI** as the active layer under a fixed V_{DS} = 60 V.

	**ABQD**	**AB2QD**	**AB3QD**
μ (cm^2 V^{-1} s^{-1})[a]	2.25×10^{-3}	8.77×10^{-3}	1.51×10^{-2}
$V_{th, write}$ (V)	–3.6	–4.2	–4.5
$V_{th, erase}$ (V)	12.0	9.2	10.6
Δ V_{th} (V)	15.6	13.4	15.1
I_{on} (A)	5.38×10^{-8}	4.56×10^{-8}	5.33×10^{-7}
I_{off} (A)	5.16×10^{-12}	2.75×10^{-12}	3.04×10^{-12}
$I_{on/off}$	1.04×10^{4}	1.66×10^{4}	1.75×10^{5}

[a] Carrier mobility corrected with each capacitance.

Table S7. Phototransistor memory device parameters derived from the transfer curves for BCPs/QDs at a ratio of 8/2 with **NDI** as the active layer under a fixed V_{DS} = 60 V.

	**ABQD**	**AB2QD**	**AB3QD**
μ (cm^2 V^{-1} s^{-1})[a]	6.17×10^{-3}	8.74×10^{-3}	1.36×10^{-2}

$V_{th, write}$ (V)	-1.6	-2.4	-4.2
$V_{th, erase}$ (V)	13.0	10.7	11.3
ΔV_{th} (V)	14.6	13.1	15.5
I_{on} (A)	6.02×10^{-8}	5.71×10^{-8}	4.33×10^{-7}
I_{off} (A)	1.19×10^{-13}	4.30×10^{-13}	1.40×10^{-12}
$I_{on/off}$	5.06×10^{4}	1.33×10^{5}	3.09×10^{5}

[a] Carrier mobility corrected with each capacitance.

Table S8. Phototransistor memory device parameters derived from the transfer curves for BCPs/QDs at a ratio of 7/3 with **NDI** as the active layer under a fixed V_{DS} = 60 V.

	**ABQD**	**AB2QD**	**AB3QD**
μ (cm^2 V^{-1} s^{-1})[a]	2.51×10^{-4}	1.32×10^{-3}	1.35×10^{-3}
$V_{th, write}$ (V)	-4.1	-5.0	-8.5
$V_{th, erase}$ (V)	15.1	9.0	7.4
ΔV_{th} (V)	19.2	14.0	15.9
I_{on} (A)	5.00×10^{-9}	4.06×10^{-8}	2.42×10^{-7}
I_{off} (A)	1.01×10^{-12}	8.15×10^{-12}	3.80×10^{-12}
$I_{on/off}$	4.95×10^{3}	4.98×10^{3}	6.37×10^{4}

[a] Carrier mobility corrected with each capacitance.

Table S9. Phototransistor memory device parameters derived from the transfer curves for BCPs/QDs at a ratio of 9/1 with **PDI** as the active layer under a fixed V_{DS} = 60 V.

	**ABQD**	**AB2QD**	**AB3QD**
μ (cm^2 V^{-1} s^{-1})[a]	1.22×10^{-3}	4.66×10^{-3}	1.91×10^{-2}
$V_{th, write}$ (V)	1.4	0.0	1.2
$V_{th, erase}$ (V)	8.9	4.5	8.0
ΔV_{th} (V)	7.5	4.5	6.8
I_{on} (A)	5.58×10^{-9}	5.48×10^{-8}	1.03×10^{-7}
I_{off} (A)	3.91×10^{-11}	5.45×10^{-12}	2.30×10^{-12}
$I_{on/off}$	1.43×10^{2}	1.01×10^{4}	4.48×10^{4}

[a] Carrier mobility corrected with each capacitance.

Table S10. Phototransistor memory device parameters derived from the transfer curves for BCPs/QDs at a ratio of 8/2 with **PDI** as the active layer under a fixed V_{DS} = 60 V.

	**ABQD**	**AB2QD**	**AB3QD**
μ (cm^2 V^{-1} s^{-1})^a	7.91×10^{-4}	4.95×10^{-3}	9.82×10^{-3}
$V_{th,\ write}$ (V)	-0.3	-0.3	-0.9
$V_{th,\ erase}$ (V)	9.9	8.0	4.9
$\Delta\ V_{th}$ (V)	10.2	8.3	5.8
I_{on} (A)	1.72×10^{-9}	2.86×10^{-8}	1.43×10^{-8}
I_{off} (A)	2.32×10^{-11}	2.06×10^{-11}	2.13×10^{-12}
$I_{on/off}$	7.41×10^{1}	1.39×10^{3}	6.71×10^{3}

^{a} Carrier mobility corrected with each capacitance.

Table S11. Phototransistor memory device parameters derived from the transfer curves for BCPs/QDs at a ratio of 7/3 with **PDI** as the active layer under a fixed V_{DS} = 60 V.

	**ABQD**	**AB2QD**	**AB3QD**
μ (cm^2 V^{-1} s^{-1})^a	6.33×10^{-3}	5.79×10^{-3}	6.77×10^{-3}
$V_{th,\ write}$ (V)	0.7	-1.1	-0.7
$V_{th,\ erase}$ (V)	11.8	7.0	6.1
$\Delta\ V_{th}$ (V)	11.1	8.1	6.8
I_{on} (A)	9.56×10^{-10}	6.19×10^{-10}	2.00×10^{-8}
I_{off} (A)	2.30×10^{-11}	2.48×10^{-12}	1.84×10^{-12}
$I_{on/off}$	4.16×10^{1}	2.50×10^{2}	1.09×10^{4}

^{a} Carrier mobility corrected with each capacitance.

Table S12. Phototransistor memory device parameters derived from the transfer curves for BCPs/QDs at a ratio of 9/1 with **PMDI** as the active layer under a fixed V_{DS} = 60 V.

	**ABQD**	**AB2QD**	**AB3QD**
μ (cm^2 V^{-1} s^{-1})^a	1.20×10^{-3}	2.33×10^{-3}	2.52×10^{-3}
$V_{th,\ write}$ (V)	37.4	29.4	26.5
$V_{th,\ erase}$ (V)	48.4	49.4	52.7
$\Delta\ V_{th}$ (V)	11.0	20.0	26.2
I_{on} (A)	7.39×10^{-13}	1.33×10^{-11}	3.47×10^{-12}
I_{off} (A)	2.56×10^{-12}	6.12×10^{-12}	1.03×10^{-11}
$I_{on/off}$	--	2.2	--

^{a} Carrier mobility corrected with each capacitance.

Table S13. Phototransistor memory device parameters derived from the transfer curves for BCPs/QDs at a ratio of 8/2 with **PMDI** as the active layer under a fixed V_{DS} = 60 V.

	**ABQD**	**AB2QD**	**AB3QD**
μ ($cm^2 V^{-1} s^{-1}$)[a]	2.14×10^{-3}	1.79×10^{-3}	5.08×10^{-3}
$V_{th, write}$ (V)	34.3	32.1	32.0
$V_{th, erase}$ (V)	52.4	47.7	52.2
ΔV_{th} (V)	18.1	15.6	20.2
I_{on} (A)	2.94×10^{-12}	2.52×10^{-12}	2.40×10^{-12}
I_{off} (A)	1.68×10^{-12}	1.68×10^{-12}	1.03×10^{-11}
$I_{on/off}$	--	1.5	--

[a] Carrier mobility corrected with each capacitance.

Table S14. Phototransistor memory device parameters derived from the transfer curves for BCPs/QDs at a ratio of 7/3 with **PMDI** as the active layer under a fixed V_{DS} = 60 V.

	**ABQD**	**AB2QD**	**AB3QD**
μ ($cm^2 V^{-1} s^{-1}$)[a]	6.33×10^{-4}	3.10×10^{-4}	8.56×10^{-4}
$V_{th, write}$ (V)	45.2	25.4	29.0
$V_{th, erase}$ (V)	46.9	30.4	54.2
ΔV_{th} (V)	1.7	5.0	25.2
I_{on} (A)	4.59×10^{-12}	5.76×10^{-12}	4.59×10^{-12}
I_{off} (A)	2.61×10^{-12}	2.44×10^{-12}	1.03×10^{-11}
$I_{on/off}$	--	2.4	--

[a] Carrier mobility corrected with each capacitance.

Table S15. The optical parameters of the BCP/QDs composite films derived from the TRPL measurement with an excitation wavelength of 510 nm.

	A_1	τ_1 (ns)	A_2	τ_2 (ns)	τ_{avg} (ns)	*PLQY* (%)
**Pure QD**	0.337	0.430	0.561	2.503	2.309	18.7
**ABQD**	0.399	0.483	0.504	2.558	2.288	1.67
**AB2QD**	0.346	0.542	0.513	2.573	2.321	5.72
**AB3QD**	0.386	0.481	0.523	2.600	2.345	7.47

Table S16. The optical parameters of the BCP/QDs based on **NDI** films derived from the TRPL measurement with an excitation wavelength of 510 nm.

Material	A_1	τ_1 (ns)	A_2	τ_2 (ns)	τ_{avg} (ns)	*CTE*[a] *(%)*	k_{CT}[a] (ns^{-1})	*PL quenching*[b] *(%)*	*PLQY* (%)
**Pure NDI**	0.700	0.250	0.450	0.700	0.539	--	--	--	--
**ABQD/NDI**	0.799	0.210	0.800	0.310	0.270	88.2	3.27	90.4	6.78
**AB2QD/NDI**	0.813	0.157	0.894	0.267	0.229	90.1	3.94	90.6	7.34
**AB3QD/NDI**	0.831	0.105	0.900	0.157	0.137	94.2	6.87	97.5	9.79

[a]*CTE* (%) was calculated as following equation:

$$CTE\ (\%) = \frac{\tau_{bilayer} - \tau_{BCP/QDs}}{\tau_{BCP/QDs}} \times 100\%$$

$$\frac{1}{\tau_{bilayer}} = \frac{1}{\tau_{BCP/QDs}} - \frac{1}{\tau_{CT}}$$

[b]*PL quenching (%)* was calculated based on PL measurements with a 365-nm light excitation.

Table S17. The optical parameters of the BCP/QDs based on **PMDI** films derived from the TRPL measurement with an excitation wavelength of 510 nm

Material	A_1	τ_1 (ns)	A_2	τ_2 (ns)	τ_{avg} (ns)	*CTE*[a] *(%)*	k_{CT}[a] (ns^{-1})	*PL quenching*[b] *(%)*	*PLQY* (%)
**ABQD/PMDI**	0.569	0.310	0.400	1.881	1.582	30.9	0.19	57.1	2.01
**AB2QD/PMDI**	0.587	0.284	0.377	1.895	1.589	31.5	0.20	56.1	4.31
**AB3QD/PMDI**	0.500	0.479	0.454	1.887	1.579	32.7	0.21	87.1	5.51

[a]*CTE* (%) was calculated as following equation:

$$CTE\ (\%) = \frac{\tau_{bilayer} - \tau_{BCP/QDs}}{\tau_{BCP/QDs}} \times 100\%$$

$$\frac{1}{\tau_{bilayer}} = \frac{1}{\tau_{BCP/QDs}} - \frac{1}{\tau_{CT}}$$

where $1/\tau_{CT} = k_{CT}$ (ns^{-1}). [b]*PL quenching (%)* was calculated based on PL measurements with a 365-nm light excitation.

Table S18. The optical parameters of the BCP/QDs based on **PDI** films derived from the TRPL measurement with an excitation wavelength of 630 nm.

Material	A_1	τ_1 (ns)	A_2	τ_2 (ns)	τ_{avg} (ns)	*CTE*[a] *(%)*	k_{CT}[a] (ns^{-1})	*PL quenching*[b] *(%)*	*PLQY*
**Pure PDI**	0.527	0.208	0.493	0.607	0.500	--	--	--	--
**ABQD/PDI**	0.641	0.234	0.411	0.661	0.509	1.8	0.03	56.6	--
**AB2QD/PDI**	0.618	0.247	0.432	0.674	0.527	5.4	0.07	84.4	--
**AB3QD/PDI**	0.521	0.251	0.504	0.742	0.615	23.0	0.27	99.0	--

[a]*CTE* (%) was calculated as following equation:

$$CTE\,(\%) = \frac{\tau_{bilayer} - \tau_{PDI}}{\tau_{PDI}} \times 100\%$$

$$\frac{1}{\tau_{bilayer}} = \frac{1}{\tau_{PDI}} - \frac{1}{\tau_{CT}}$$

where $1/\tau_{CT} = k_{CT}$ (ns^{-1}). Note that the PL emission of QD is fully quenched by the PDI layer because of the strong light absorption of PDI to the excitation light of 510 nm. The emission of **PDI** excited by a 630-nm light was applied to characterized the charge transfer capability between the **PDI** channel and the floating gate layer. [b]*PL quenching (%)* was calculated based on the PL measurements with a 365-nm light excitation.

Synthesis of 4-azidobutan-1-ol (OHN_3).

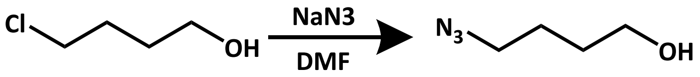

The solution of 4-chlorobutan-1-ol (20 g, 184.2 mmole) and sodium azide (24 g, 221.0 mmole) in the 200 ml DMF was stirred under argon at 80 °C overnight. After removal of the DMF, the crude was purified by the flash column chromatography (pure EA as an eluent) to provide the colorless oil **OHN_3** (19.1 g, yield = 90%). Anal. calcd. For [$C_4H_9N_3O$] (%): C: 41.05, H: 7.88, N: 36.50. Found (%): C: 40.82, H: 7.91, N: 36.42. ^1H NMR (400 MHz, $CDCl_3$, **Figure S1**) δ (ppm): 3.59 (t, 2H), 3.26 (t, 2H), 2.14 (s, 1H), 1.59 (m, 4H).

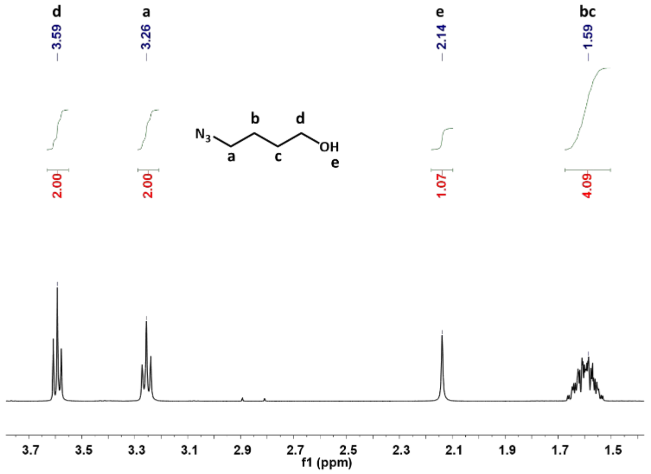

Figure S1. ^1H NMR spectrum of **OH-N3** in $CDCl_3$.

Synthesis of 4-azidobutyl 4-methylbenzenesulfonate ($OTsN_3$).

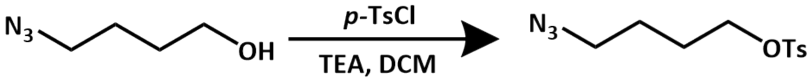

A solution of **OHN_3** (14.7 g, 127.7 mmole) and triethylamine (15.5 g, 153.2 mmole) in 250 ml DCM at 0 °C was added *p*-toluenesulfonyl chloride (29.2 g, 153.2 mmole) in one portion. The mixture was warmed to room temperature and stirred at for 18 hr under argon. The reaction was extracted with brine and water, and the organic phase was dried over magnesium sulfate *in vacuo*. The crude was purified by column chromatography (Hexane: ethyl acetate = 9:1, R_f = 0.3, to 1:1, R_f = 0.5) to yield as a colorless oil **$OTsN_3$** (32.4 g, yield = 93.1 %). Anal. calcd. For [$C_{11}H_{15}N_3O_3$] (%): C: 49.06, H: 5.61, N: 15.60. Found (%): C: 48.97, H: 5.88, N: 15.31. ^1H NMR (400 MHz, $CDCl_3$, **Figure S2**) δ (ppm): 7.71 (d, 2H), 7.29 (d, 2H), 3.99 (t, 2H), 3.19 (t, 2H), 2.38 (s, 3H), 1.66 – 1.55 (m, 4H).

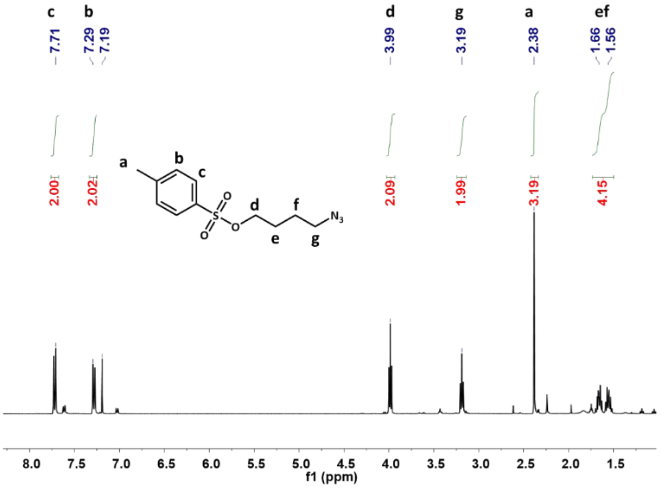

Figure S2. ^1H NMR spectrum of **OTs-N3** in $CDCl_3$.

General methods for nucleophilic substitution reaction.

To a solution of methyl benzoate derivatives (1.0 equiv) in DMF (0.1M) was degassed by argon bubble. Then, K_2CO_3 (3.3 equiv per hydroxy group) and **OTsN$_3$** (1.1 equiv per hydroxy group) were added to the mixture under reflux at 80 °C overnight. After the completion of the reaction by TLC, the mixture was cooled to room temperature and removed. After removal of the DMF by rotary evaporator, the residue was diluted with chloroform. The entire mixture was then extracted with water and brine twice, dried over magnesium sulfate, filtered, and concentrated under reduced pressure. The resulting crude was purified by column chromatography to render the corresponding oil compounds (**2N3-COOMe** and **3N3-COOMe**), which were characterized as follows:

Synthesis of Methyl 3,5-bis(4-azidobutoxy) benzoate (2N3-COOMe).

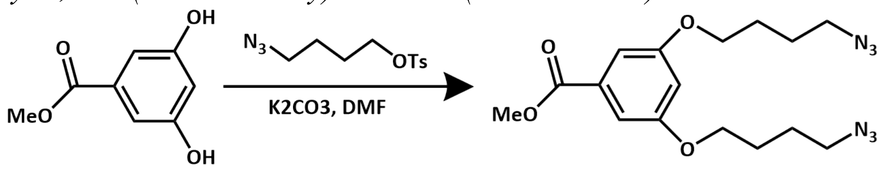

Methyl 3,5-dihydroxybenzoate (4.1 g, 24.5 mmole); **OTsN$_3$** (15.0 g, 55.7 mmole); K_2CO_3 (17.5 g, 126.6 mmole); DMF (170 ml, 0.1M); TLC (hexane: ethyl acetate = 20:1) with R_f = 0.2; a colorless oil (4.82 g, yield = 54.3 %). Anal. calcd. For [$C_{16}H_{22}N_6O_4$] (%): C: 53.03, H: 6.12, N: 23.19. Found (%): C: 52.98, H: 6.30, N: 23.04. ^{1}H NMR (400 MHz, $CDCl_3$, **Figure S3**) δ (ppm): 7.09 (d, 2H), 6.55 (d, 1H), 3.94 (t, 4H), 3.83 (s, 3H), 3.29 (t, 4H), 1.81 – 1.73 (m, 8H). ^{13}C NMR (400NMR, $CDCl_3$, **Figure S4**) δ (ppm): 166.8 (1C), 159.9 (2C), 132.0 (1C), 107.7 (2C), 106.6 (1C), 67.5 (2C), 52.3 (2C), 51.1 (3C), 26.4 (2C), 25.7 (2C).

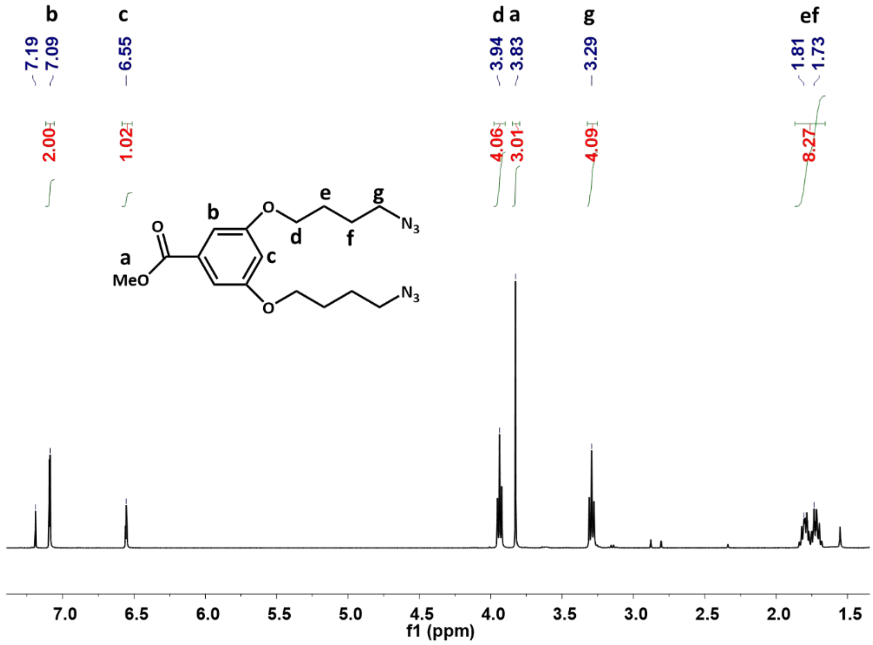

Figure S3. ^{1}H NMR spectrum of **2N3-COOMe** in CDCl3.

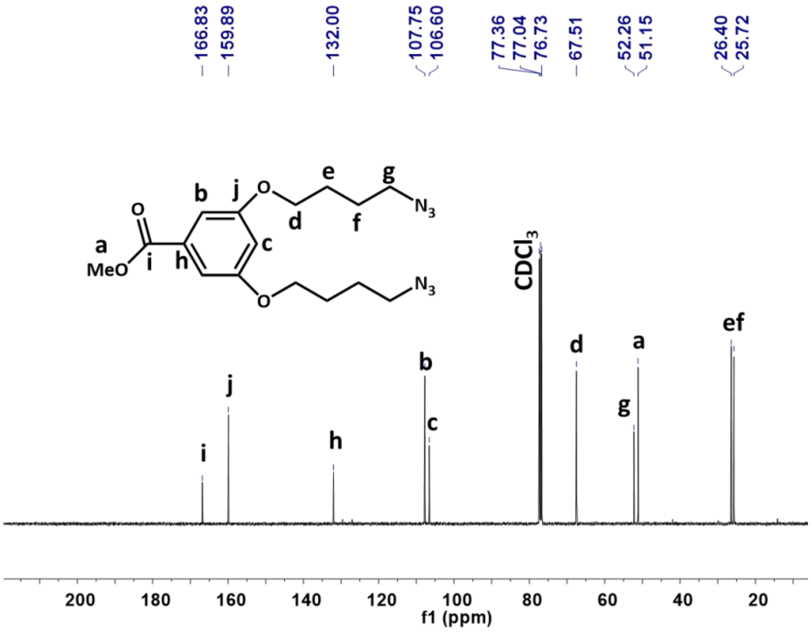

Figure S4. ^{13}C NMR spectrum of **2N3-COOMe** in $CDCl_3$.

*Synthesis of Methyl 3,4,5-tris(4-azidobutoxy) benzoate (3N3-COOMe)***.**

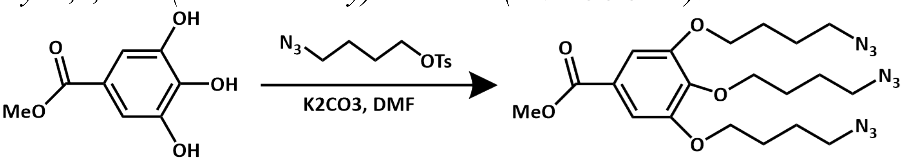

Methyl gallate (3.1 g, 184.2 mmole); **OTsN3** (15.0 g, 55.7 mmole); K_2CO_3 (22.4 g, 161.7 mmole); DMF (250 ml, 0.1M); TLC (diethyl ether: ethyl acetate = 6: 4) with R_f = 0.6; a yellow oil (7.34 g, yield = 91.4 %). Anal. calcd. For [$C_{20}H_{29}N_9O_5$] (%): C: 50.52, H: 6.15, N: 26.51. Found (%): C: 50.11, H: 6.20, N: 26.18. ^{1}H NMR (400 MHz, $CDCl_3$, **Figure S5**) δ (ppm): 7.19 (s, 2H), 3.98 (t, 6H), 3.81 (s, 3H), 3.30 (t, 6H), 1.84 – 1.76 (m, 12H). ^{13}C NMR (400NMR, $CDCl_3$, **Figure S6**) δ (ppm): 166.7 (1C), 152.5 (2C), 141.8 (1C), 125.1 (1C), 108.0 (2C), 72.6-68.4 (3C), 52.2-51.1 (6C), 27.4-25.7 (6C).

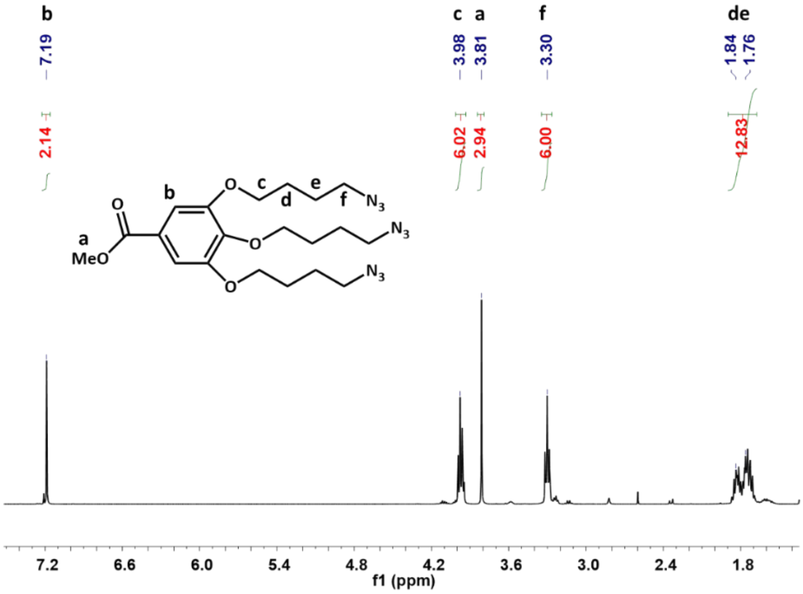

Figure S5. ^{1}H NMR spectrum of **3N3-COOMe** in $CDCl_3$.

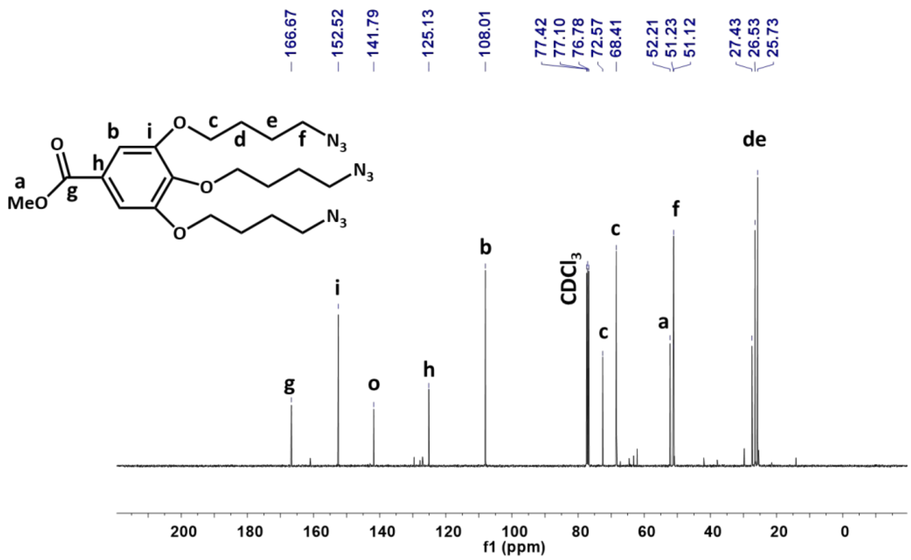

Figure S6. ^{13}C NMR spectrum of **3N3-COOMe** in $CDCl_3$.

General methods for hydrolysis reaction.

For hydrolysis of the methyl benzoate derivateives, firstly the methyl benzoate derivatives were saponificated under basic environments, and the mixtures were protonated subsequently to provide the benzoic acid derivatives, **2OH-COOH** or **3OH-COOH**. Initially, to a solution of methyl benzoate derivatives, **2OH-COOMe** in a 0.1 M mixed solvent of THF and MeOH (v/v = 2:1) or **3OH-COOMe**, (1.0 equiv) in MeOH was added KOH solution (2.0 equiv in 4.5 M water). The reaction was refluxed at 110 °C overnight. After the reaction was completed, as confirmed by TLC, it was cooled and concentrated to remove the solvent. The pH value of the crude was adjusted to 2 by adding 1 N HCl, and the mixture was extracted with ethyl acetate three times. The extract was then dried over magnesium sulfate, filtered, and concentrated to yield the corresponding products as **2OH-COOH** or **3OH-COOH**.

Synthesis of 3,5-bis(4-azidobutoxy) benzoic acid (2N3-COOH).

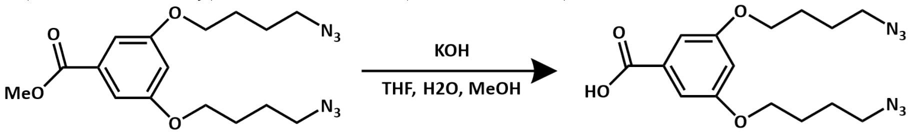

2N$_3$-COOMe (4.4 g, 12.1 mmole); KOH (2.7 g, 48.1 mmole) in 10.6 ml H_2O; MeOH (40 ml); THF (80 ml); TLC (hexane: ethyl acetate = 1:1) with R_f = 0.65; yellow oil (4.0 g, yield = 93.1 %). Anal. calcd. For [$C_{15}H_{20}N_6O_4$] (%): C: 51.72, H: 5.79, N: 24.12. Found (%): C: 51.93, H: 6.93, N: 23.44. ^{1}H NMR (400 MHz, $CDCl_3$, **Figure S7**) δ (ppm): 7.16 (d, 2H), 6.61 (t, 3H), 3.96 (t, 4H), 3.31 (t, 4H), 1.84 – 1.76 (m, 8H). ^{13}C NMR (400NMR, $CDCl_3$, **Figure S8**) δ (ppm): 171.6 (1C), 156.0 (2C), 131.2 (1C), 108.3 (2C), 107.4 (1C), 67.6 (2C), 51.2 (2C), 26.4 (2C), 25.7 (2C).

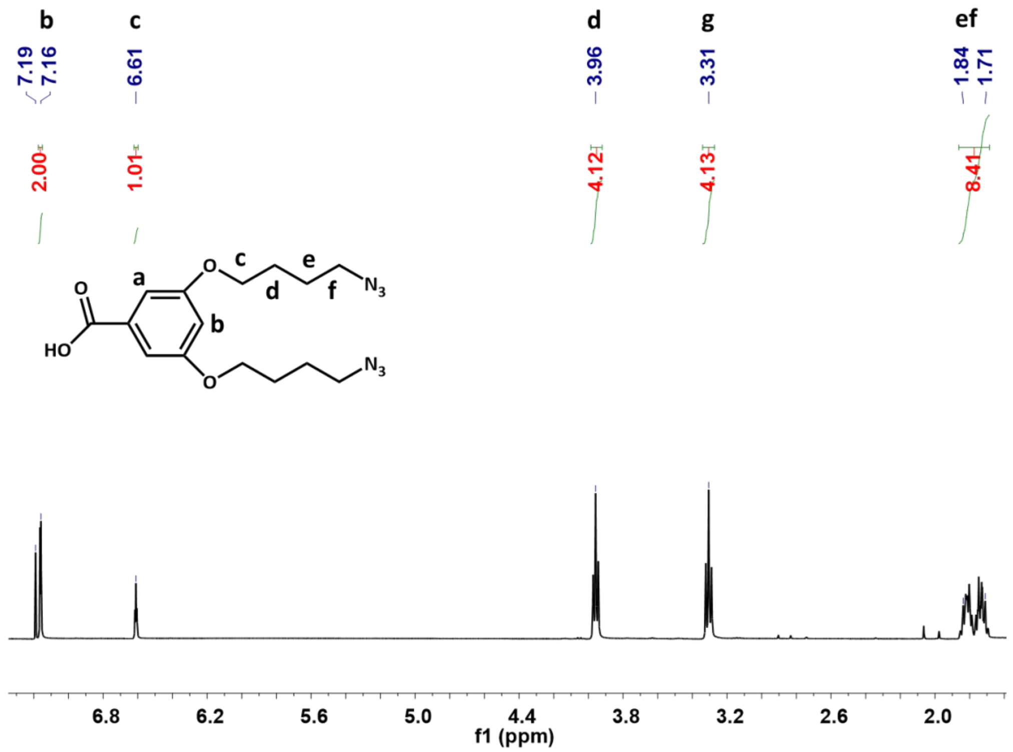

Figure S7. ^{1}H NMR spectrum of **2N3-COOH** in $CDCl_3$.

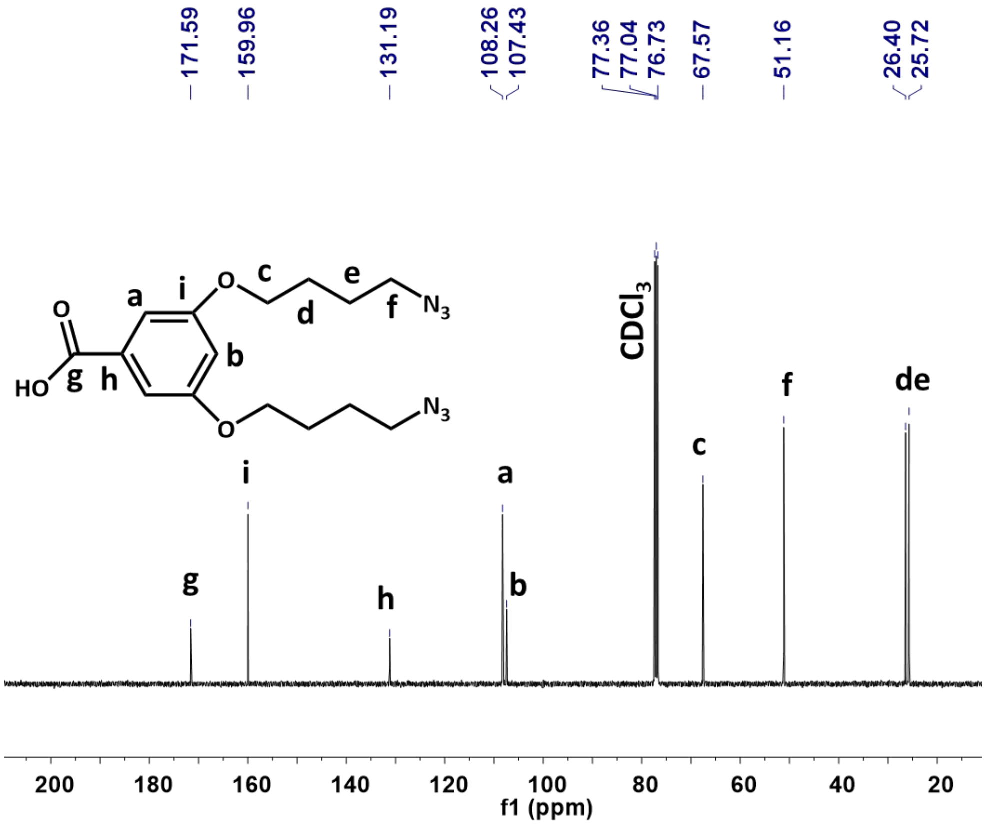

Figure S8. ^{13}C NMR spectrum of **2N3-COOH** in $CDCl_3$.

Synthesis of 3,4,5-tris(4-azidobutoxy) benzoic acid (3N3-COOH).

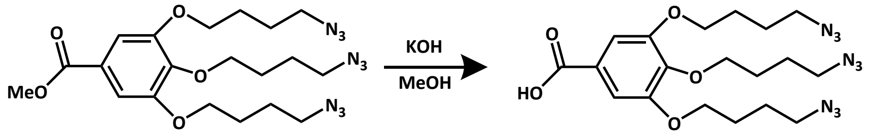

$3N_3$-COOMe (4.0 g, 8.41 mmole); KOH (2.12 g, 37.85 mmole) in 8.5 ml H_2O; MeOH (28 ml); THF (56 ml); TLC (ether: ethyl acetate = 1:1) R_f = 0.50; yellow oil (3.02 g, yield = 77.3 %). Anal. calcd. For [$C_{19}H_{27}N_9O_5$] (%): C: 49.45, H: 5.90, N: 27.32. Found (%): C: 49.28, H: 5.87, N: 27.44. ^{1}H NMR (400 MHz, $CDCl_3$, **Figure S9**) δ (ppm): 7.25 (s, 2H), 4.00 (t, 6H), 3.32 (t, 6H), 1.84 – 1.76 (m, 12H). ^{13}C NMR (400NMR, $CDCl_3$, **Figure S10**) δ (ppm): 171.1 (1C), 152.6 (2C), 142.6 (1C), 124.2 (1C), 108.6 (2C), 72.7-68.5 (3C), 51.1 (3C), 29.8-25.4 (6C).

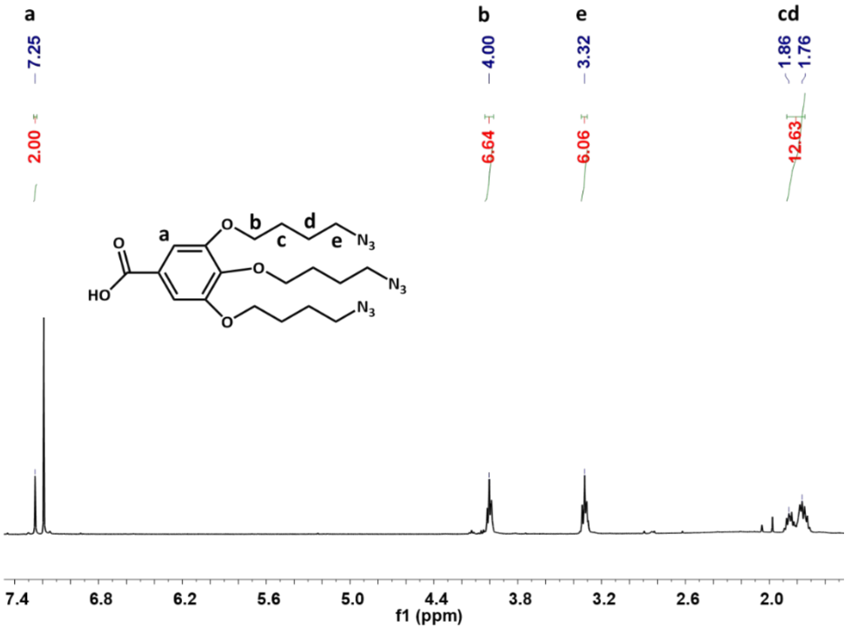

Figure S9. ^{1}H NMR spectrum of **3N3-COOH** in $CDCl_3$.

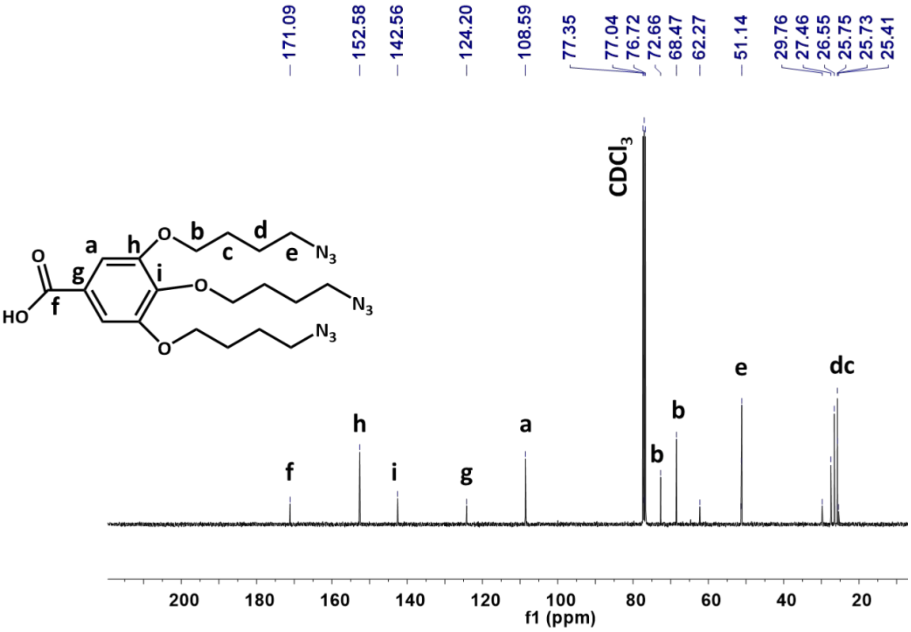

Figure S10. ^{13}C NMR spectrum of **3N3-COOH** in $CDCl_3$.

Synthesis of ω-bromo-terminated poly(dimethylsiloxane) (PDMS1Br).

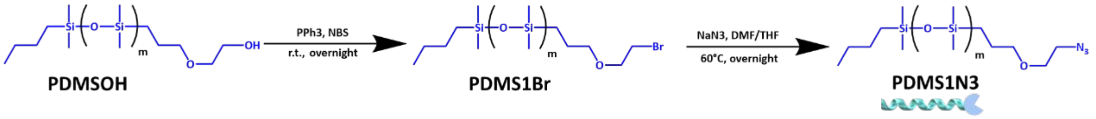

Ω-Hydroxy-terminated poly(dimethylsiloxane), **PDMSOH** and NBS were added in 0.1 M DCM in a round-bottom flask under nitrogen-filled atmosphere. Then, the solution was stirred at 0 °C before the PPh_3 was added spoonwisely at room temperature overnight in the dark. After ensuring the reaction was unchanged with TLC, the solvent was removed under vacuum, followed by column chromatography with hexane/ dichloromethane (3:1) as the eluent to provide the colorless oil products, **PDMS1Br**.

PDMS1Br. **PDMSOH** (10 g, 10 mmole); PPh_3 (3.93 g, 15 mmole); NBS (2.67 g, 15 mmole); DCM (100 ml). Yield = 70%, colorless oil. Anal. calcd. For [$C_{35}H_{97}O_{13}Si_{13}Br$] (%): C: 35.9; H: 8.4. Found (%): C: 35.1; H: 8.2. ^{1}H NMR (400 MHz, $CDCl_3$, **Figure S11b**) δ (ppm) 3.67 (t, 2H), 3.38 (t, 4H), 1.55 (m, 2H), 1.24 (m, 4H), 0.81 (t, 3H), 0.47 (m, 4H), 0.15 – (−0.15) (m, 100H). ^{13}C NMR (100 MHz, $CDCl_3$, **Figure S12**) δ (ppm) 73.0, 69.6, 29.4, 25.3, 24.4, 22.4, 16.9, 13.1, 12.8, 0.0, −0.9.

Synthesis of ω-azido-terminated poly(dimethylsiloxane). (PDMS1N3)

Ω-Bromo terminated poly(dimethylsiloxane), **PDMS1Br** and NaN_3 were dissolved in DMF/ THF (0.1 M, *vol.* 1:1) to a round flask at 60 °C overnight. After being checked by TLC, the mixture was cooled to room temperature and dried under a vacuum to remove the solvent. The product was finally purified with a flash column with pure DCM as the eluent to afford the pure compound as a transparent oil, **PDMS1N3**. The details are as follows: **PDMS1Br** (6.62 g, 6.6 mmole); NaN_3 (0.858 g, 13.2 mmole); DMF (30 ml); THF (30 ml). Yield = 97%, colorless oil. Anal. calcd. For [$C_{35}H_{97}O_{13}Si_{13}N_3$] (%): C: 37.1; H: 8.6; N: 3.7. Found (%): C: 35.2; H: 7.3; N: 3.5. ^{1}H NMR (400 MHz, $CDCl_3$, **Figure S11c**) δ (ppm) 3.53 (t, 2H), 3.37 (t, 2H), 3.29 (t, 2H), 1.55 (m, 2H), 1.24 (m, 4H), 0.81 (t, 3H), 0.47 (m, 4H), 0.15 – (−0.15) (m, 102H). ^{13}C NMR (100 MHz, $CDCl_3$, **Figure S13**) δ (ppm) 73.1, 68.4, 49.8, 25.3, 24.4, 22.4, 16.9, 13.0, 12.8, 0.0, −1.0.

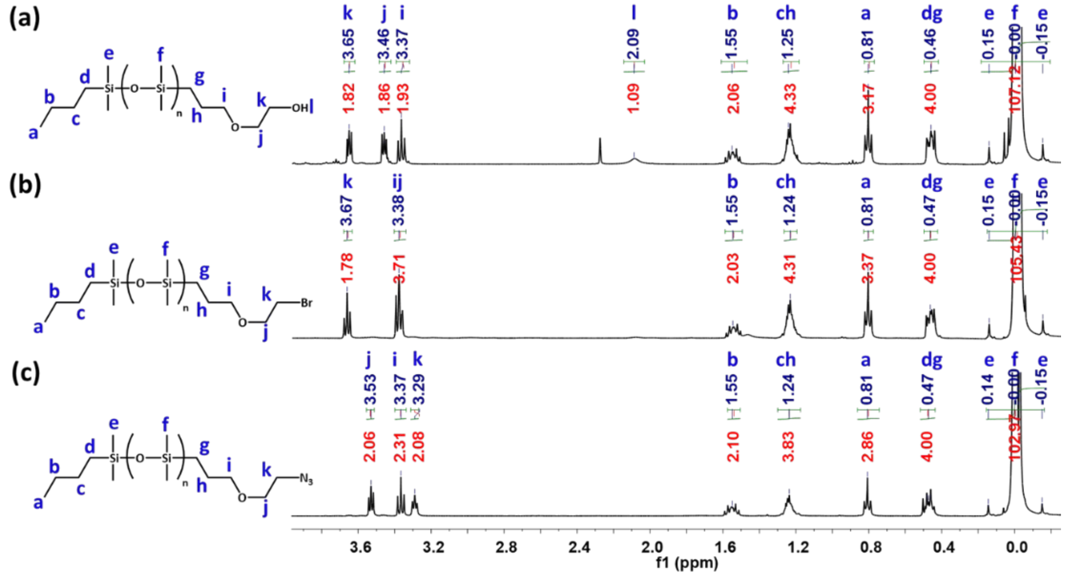

Figure S11. ^{1}H-NMR of (a) **PDMSOH**, (b) **PDMS1Br**, and (c) **PDMS1N3** in $CDCl_3$.

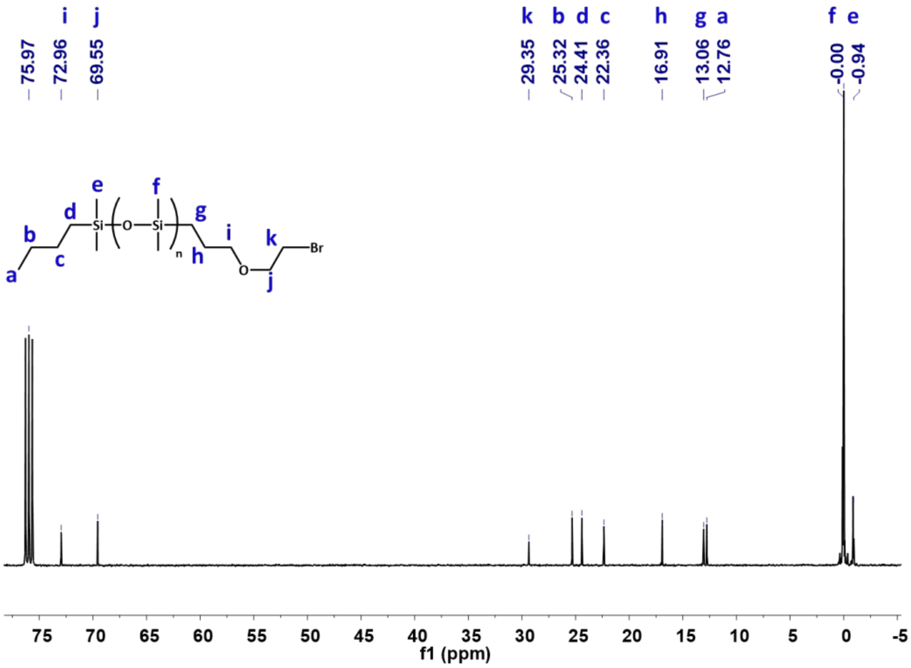

Figure S12. ^{13}C-NMR of **PDMS1Br** in $CDCl_3$.

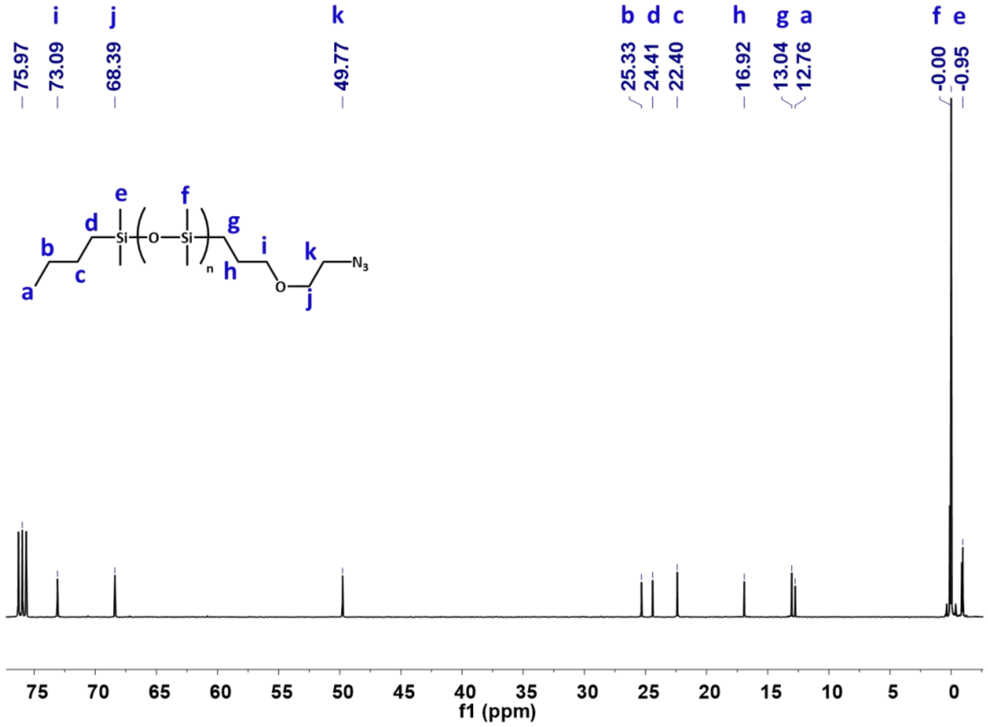

Figure S13. ^{13}C-NMR of **PDMS1N3** in $CDCl_3$.

General methods for esterification reaction. (PDMS2N3 and PDMS3N3)

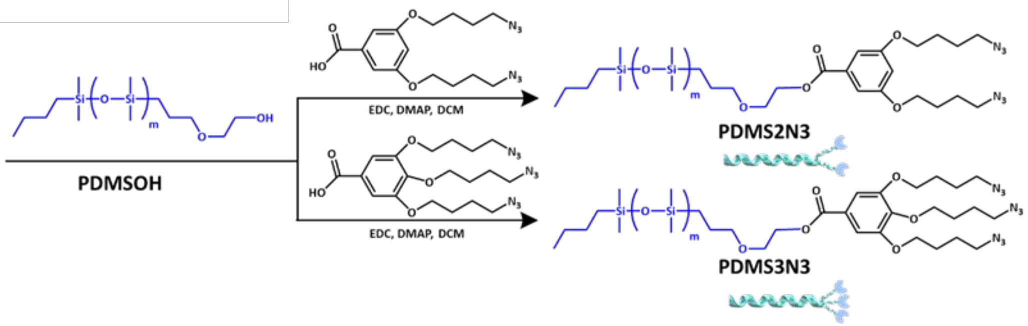

To a solution of ω−hydroxy−terminated poly(dimethylsiloxane), **PDMSOH**, in 0.1 M DCM was added with EDC (1.8 equiv per hydroxy group) and DMAP (1.8 equiv per hydroxy group) at 0 °C, followed by pouring acid derivatives, benzoic acid derivatives, **2OH-COOH** or **3OH-COOH**, under argon at room temperature for 48 hr. After this time, the reaction mixture was diluted with DCM, and the crude product was extracted with 1 N HCl, K2CO3, and H2O. The extract was then dried over magnesium sulfate, filtered, and condensed in vacuo to afford *ω*-azido-terminated PDMS bearing two and three azide groups, PDMS2N3 and PDMS3N3, respectively.

PDMS2N3. **PDMSOH** (1.90 g, 3.5 mmole); **2N3-COOH** (850 mg, 2.85 mmole); EDC (531 mg, 3.42 mmole); DMAP (418 mg, 3.42 mmole); DCM (20 ml, 0.1M). colorless oil (1.64 mg, yield = 67.2 %). Anal. Calcd. For[$C_{50}H_{116}O_6Si_{17}N_{13}$] (%): C: 41.8; H: 8.1; N: 5.8. Found (%): C: 39.8; H: 7.8; N: 5.4. ^{1}H NMR (400 MHz, $CDCl_3$, **Figure S14**) *δ* (ppm): 7.11 (s, 2H), 4.36 (s, 1H), 4.36 (s, 2H), 3.94 (t, 2H), 3.68 (t, 2H), 3.40 (t, 2H), 3.29 (t, 4H), 1.79−1.83 (m, 8H), 1.56 (m, 2H), 1.23(m, 4H), 0.81 (t, 3H), 0.46 (m, 4H), 0.15 – (−0.15) (m, 94H). ^{13}C NMR (400NMR, $CDCl_3$, **Figure S15**) *δ* (ppm) 166.4, 159.9, 132.0, 107.9, 106.5, 74.2, 68.5, 67.5, 64.4, 51.2, 26.4, 25.7, 25.4, 23.4, 13.8, 1.2, 1.1, 0.2, 0.1.

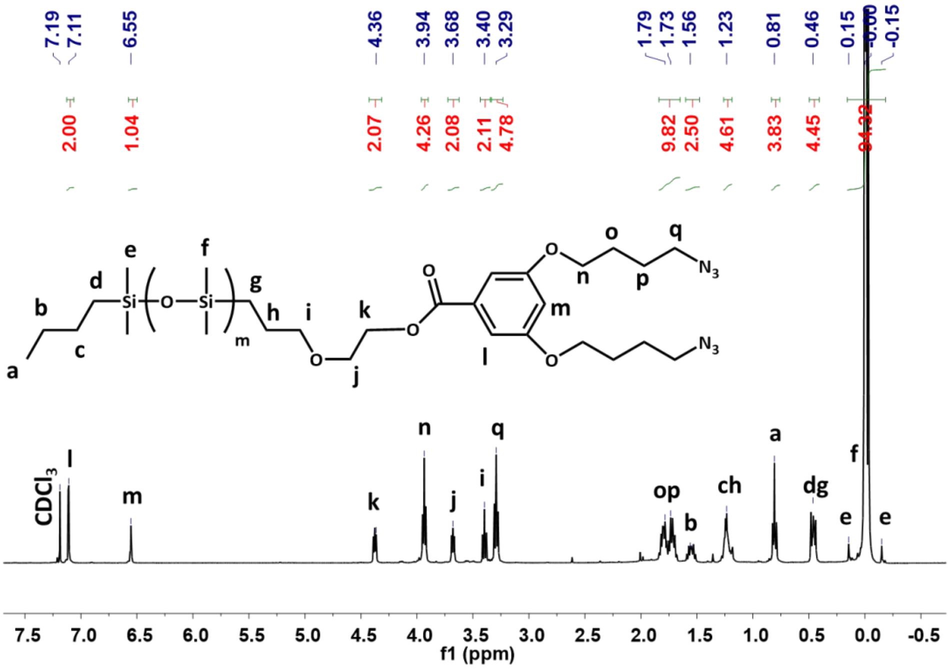

Figure S14. ^{1}H NMR spectrum of **PDMS2N3** in $CDCl_3$.

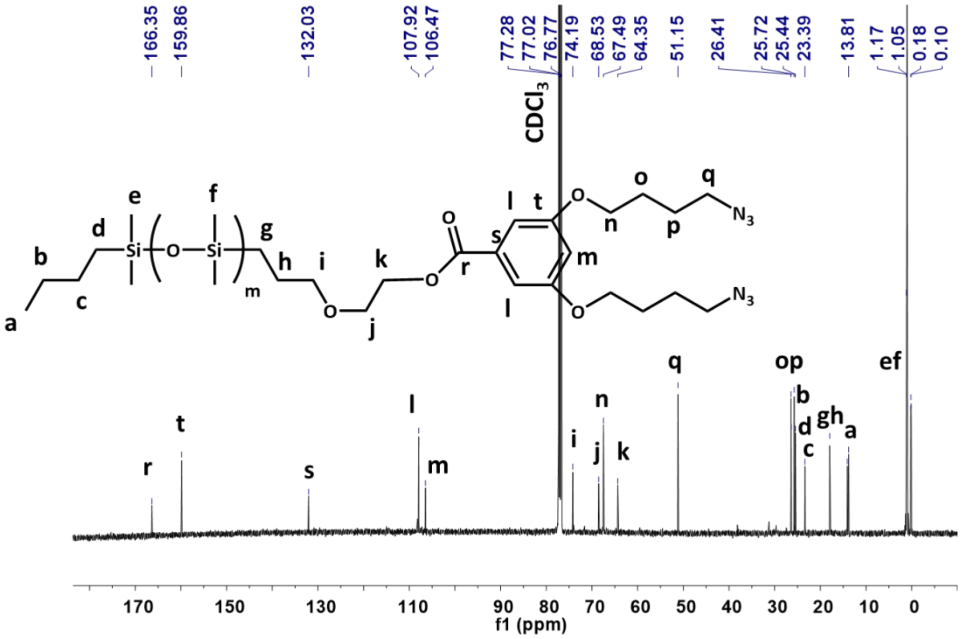

Figure S15. ^{13}C NMR spectrum of **PDMS2N3** in $CDCl_3$.

PDMS3N3. **PDMSOH** (3.50 g, 3.5 mmole); **3N3-COOH** (2.03 g, 5.25 mmole); EDC (978 mg, 6.3 mmole); DMAP (770 mg, 6.3 mmole); DCM (35 ml, 0.1M). colorless oil (3.64 g, yield = 76.0 %). Anal. Calcd. For[$C_{54}H_{123}O_9Si_{18}N_{13}$] (%): C: 41.8; H: 8.0; N: 8.1. Found (%): C: 43.2; H: 7.3; N: 7.9. ^1H NMR (400 MHz, $CDCl_3$, **Figure S16**) δ (ppm): 7.25 (s, 2H), 4.38 (t, 2H), 3.98 (t, 6H), 3.68 (t, 2H), 3.40 (t, 2H), 3.31 (t, 6H), 1.84 – 1.76 (m, 12H), 1.53 (m, 2H), 1.23 (m, 4H), 0.81 (t, 3H), 0.46 (m, 4H), 0.15 – (−0.15) (m, 109H). ^{13}C NMR (400NMR, $CDCl_3$, **Figure S17**) δ (ppm): 166.2, 152.6, 152.5, 141.9, 125.2, 108.2, 72.6, 68.6, 68.5, 64.3, 51.3, 51.1, 29.7, 25.8, 25.6, 23.4, 18.0, 14.1, 13.8, 1.1, 0.8, 0.2, 0.1.

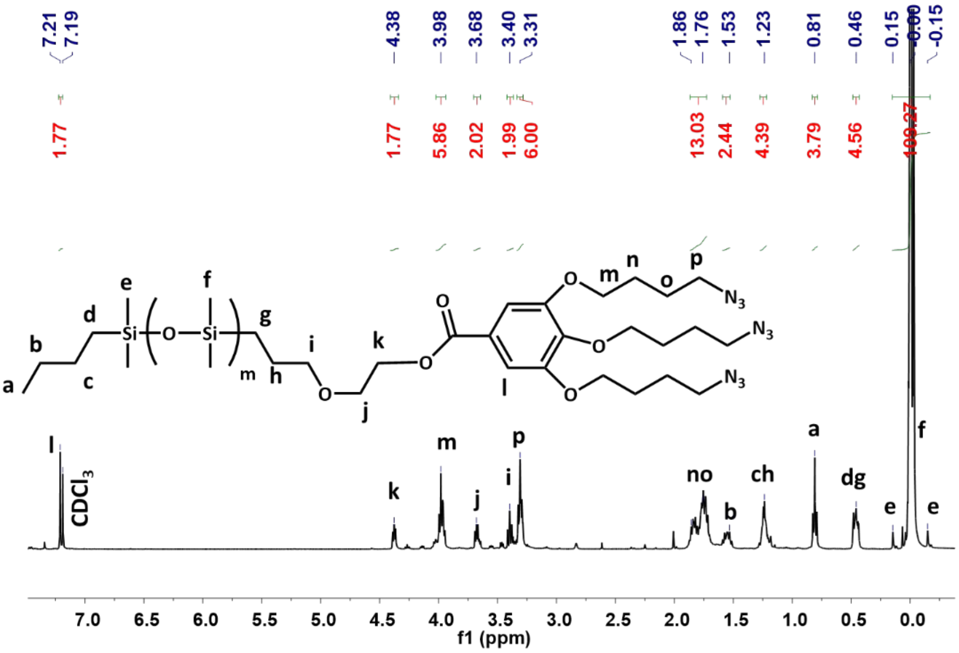

Figure S16. ^1H NMR spectrum of **PDMS3N3** in $CDCl_3$.

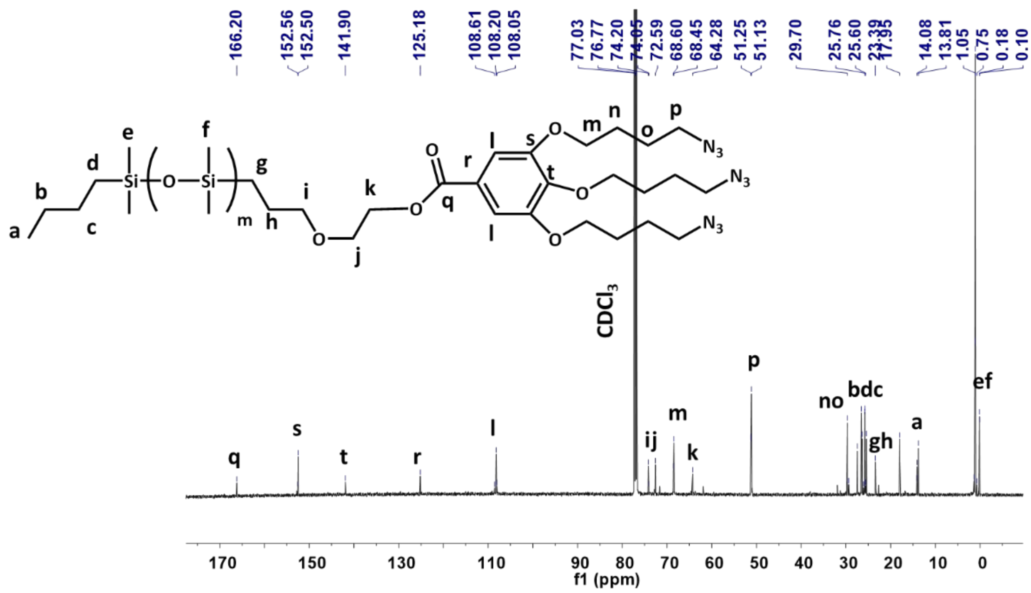

Figure S17. ^{13}C NMR spectrum of **PDMS3N3** in $CDCl_3$.

General polymerization by azide−alkyne cycloaddition click reaction (AB, AB2, and AB3).

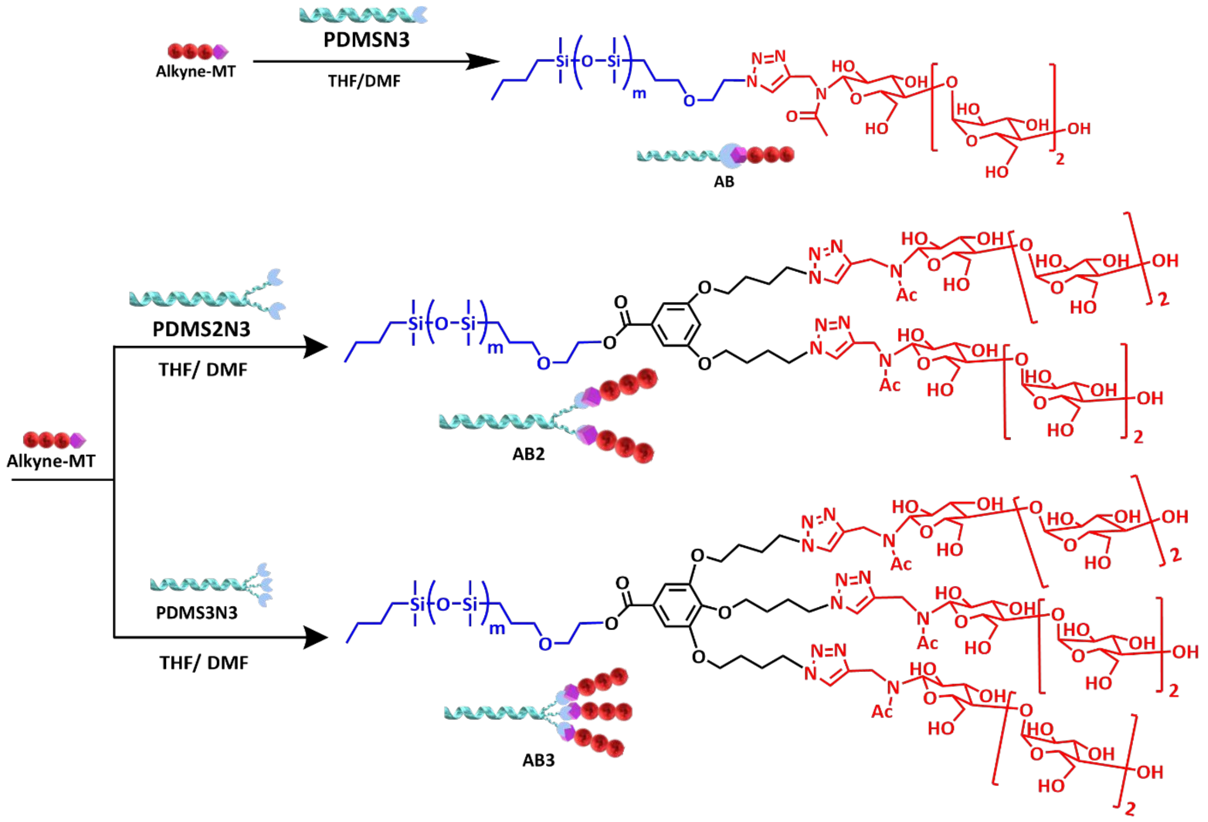

Under an argon atmosphere, azide-terminated poly(dimethylsiloxane) (1 equiv), which was prepared according to previous methods reported by I. Otsuka, et al. *Langmuir*, **2010**, *26*, 2325–2332: **PDMS1N3**, **PDMS2N3**, or **PDMS3N3**, and **alkyne-MT** (1.2 equiv per azide group) were poured into a solution of 0.1 M DMF/THF (v/v = 1:1) in a three-neck flask after three consecutive freeze−thaw cycles to degass the mixtures. Afterward, the reaction was added to copper nanopowder in a portion and stirred at 65 °C for four days. After cooling down to room temperature, the solvent was removed

under vacuum. The black crude was diluted with pure ethanol/DCM, filtered through diatomaceous earth to remove the copper nanopowder, followed by precipitation of the crude with acetone to remove the azide-terminated poly(dimethylsiloxane). The sample was characterized by FT-IR, showing the disappearance of the azide group at 2110 cm^{-1}. The unreacted **alkyne-MT** was removed by dialysis using Spectra/Por 7 dialysis tubing (1 kDa MWCO) against water over 4 days. Finally, the white solids, **AB, AB2,** and **AB3**, were obtained by lyophilization overnight, providing yields beyond 70%.

AB. **PDMS1N$_3$** (1 g, 1 mmole); **alkyne-MT** (720 mg, 1.2 mmole); Cu nanopowder (128 mg, 2 mmole); DMF (5 ml); THF (5 ml). Yield = 72%, white solids. Anal. calcd. for [$C_{42}H_{82}O_{26}Si_{13}N_2$] (%): C:38.5; H: 6.3; N: 2.3. Found (%): C: 41.7; H: 7.5; N: 3.7. ^{1}H NMR (400 MHz, MeOD**, Figure S18c**) δ (ppm): 7.93 and 7.77 (2 × s, 1H, rotamers), 5.55 and 4.89 (2 × d, 1H, rotamers), 5.05 (m, 2H), 4.49 – 3.31 (m, 34H), 2.11 – 1.94 (2 × s, 3H, rotamers), 1.49 (m, 2H), 1.25 (m, 4H), 0.83 (m, 3H), 0.47 (m, 4H), 0.15 – (−0.15) (m, 96H). ^{13}C NMR (100 MHz, MeOD**, Figure S19**) δ (ppm): 174.2, 147.9, 125.5, 102.9, 102.8, 88.9, 80.9, 74.8, 73.4, 69.9, 27.4, 26.7, 24.4, 21.9, 18.9, 15.0, 1.4, 0.4.

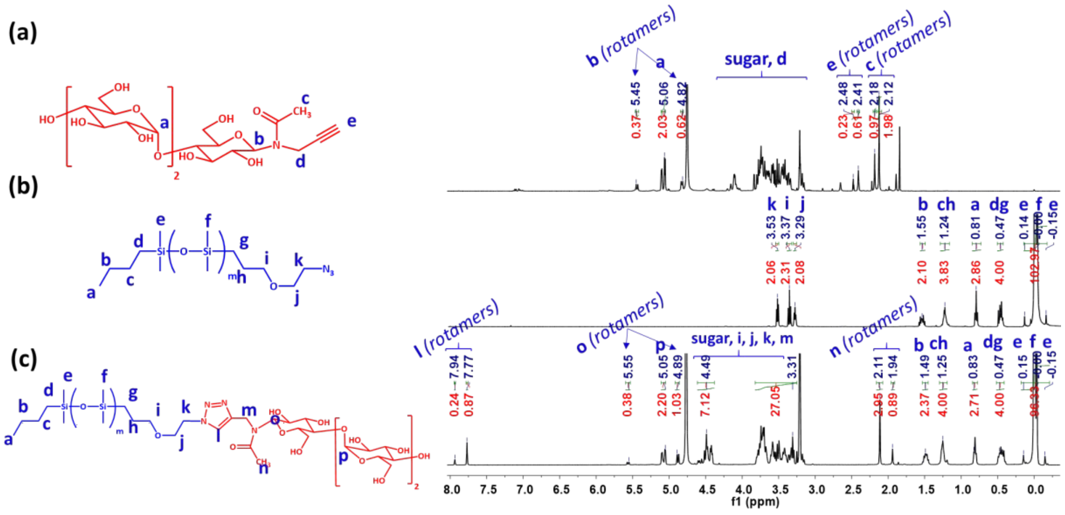

Figure S18. ^{1}H-NMR of (a) **alkyne-MT** in D_2O, (b) **PDMS1N3** in $CDCl_3$, and (c) **AB** in MeOD.

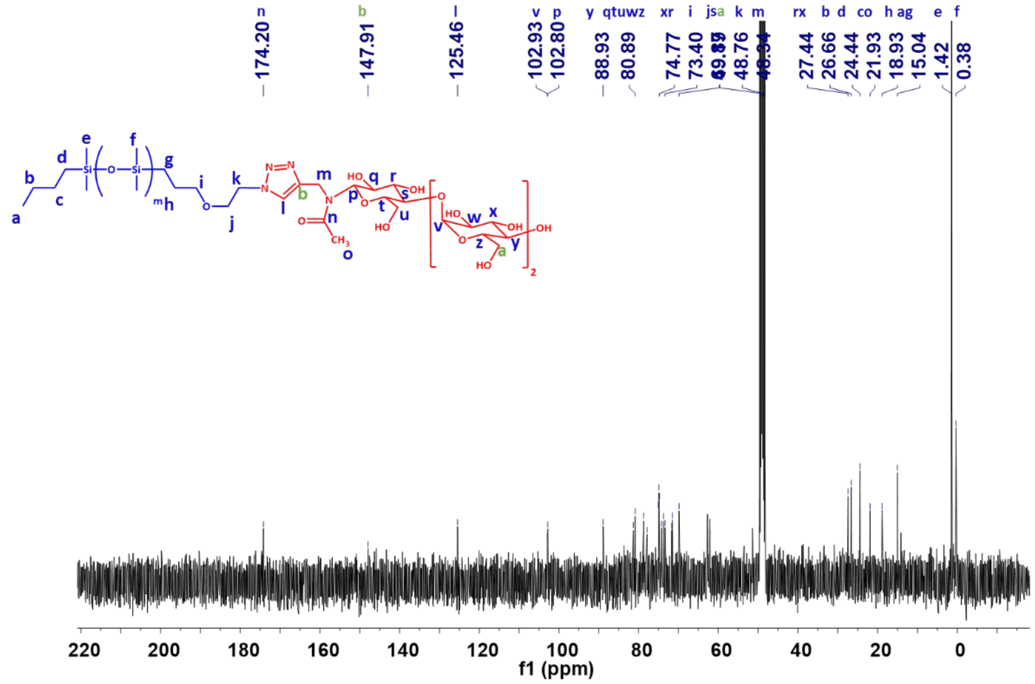

Figure S19. ^{13}C NMR spectrum of **AB** in MeOD.

AB2. **PDMS2N$_3$** (1.5 g, 1.13 mmole); **alkyne-MT** (1.6 g, 2.71 mmole); Cu nanopowder (347 mg, 5.42 mmole); DMF (5.5 ml); THF (5.5 ml). Yield = 78%, white solids. Anal. calcd. For [$C_{80}H_{146}O_{48}Si_{13}N_4$] (%): C: 44.8; H: 6.8; N: 3.3. Found (%): C: 44.0; H: 7.1; N: 4.3. ^{1}H NMR (400 MHz, *d*$_6$-DMSO**, Figure S20**) δ (ppm): 8.07 and 7.93 (2 × s, 2H, rotamers), 7.01 (m, 2H), 6.70 (s, 1H), 5.71 – 3.05 (m, 82H), 5.48 and 4.89 (2 × s, 2H, rotamers), 2.49 and 2.10 (2 × s, 6H, rotamers), 1.92 (s, 4H), 1.48 (m, 4H), 1.21 (m, 4H), 0.76 (m, 3H), 0.43 (m, 4H), 0.15 – (−0.15) (m, 94H). ^{13}C NMR (100 MHz, d$_6$-DMSO, **Figure S21**) δ (ppm): 171.0, 159.8, 145.1, 123.5, 107.5, 100.8, 100.7, 86.9, 77.3, 73.4, 71.8, 67.2, 60.8, 60.3, 49.3, 49.1, 29.8, 24.9, 21.8, 17.3, 13.4, 13.0, 0.6, 0.1, -0.2.

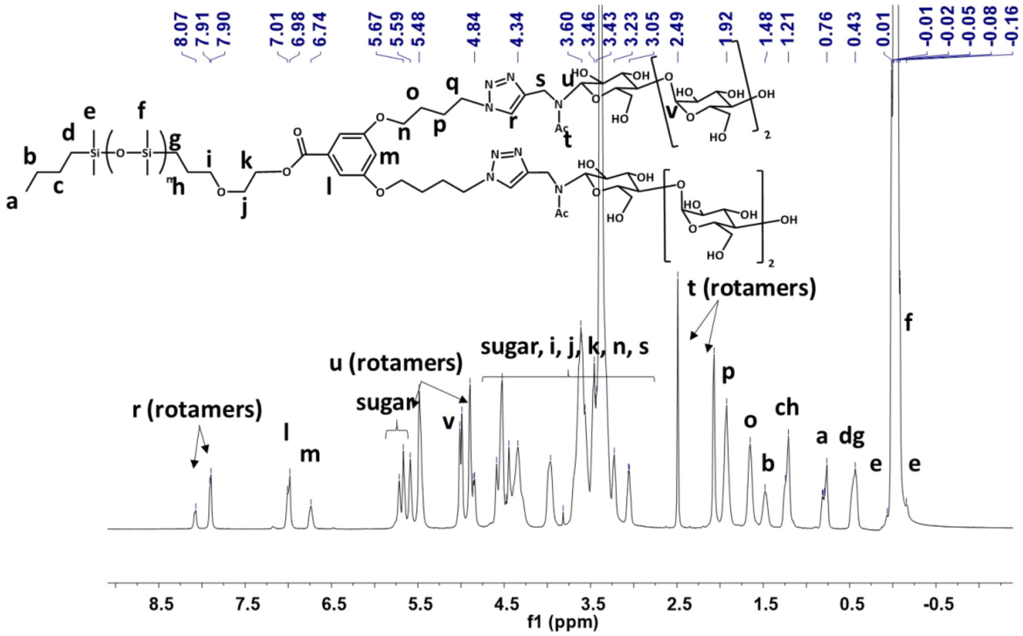

Figure S20. ^{1}H NMR spectrum of **AB2** in *d*$_6$-DMSO.

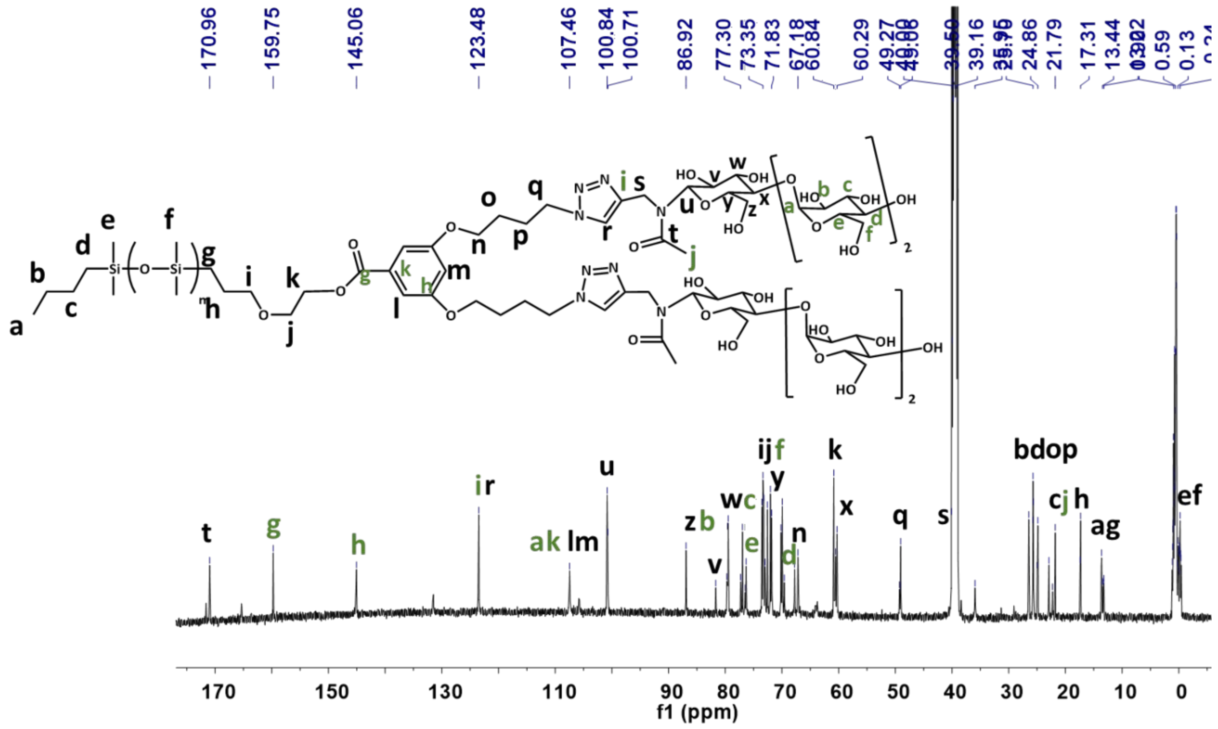

Figure S21. ^{13}C NMR spectrum of **AB2** in *d*$_6$-DMSO.

AB3. **PDMS3N$_3$** (3 g, 2.08 mmole); **alkyne-MT** (4.5 g, 7.48 mmole); Cu nanopowder (957 mg, 14.96 mmole); DMF (10.5 ml); THF (10.5 ml). Yield = 72%, white solids. Anal. calcd. For [$C_{82}H_{154}O_{50}Si_2N_6$] (%): C: 44.2; H: 7.0; N: 3.8. Found (%): C: 43.7; H: 7.0; N: 4.7. ^{1}H NMR (400 MHz, d_6-DMSO, **Figure S22**) δ (ppm): 8.06 and 7.90 (2 × s, 3H, rotamers), 7.18 (s, 2H), 5.67 – 3.03 (m, 114H), 5.44 and 4.89 (2 × s, 3H, rotamers), 2.49 and 1.98 (2 × s, 9H, rotamers), 1.82 (s, 6H), 1.64 (m, 8H), 1.21 (m, 4H), 0.77 (m, 3H), 0.43 (m, 4H), 0.15 – (−0.15) (m, 109H). ^{13}C NMR (100 MHz, d$_6$-DMSO, **Figure S23**) δ (ppm): 171.0, 152.3, 145.1, 141.3, 123.5, 107.5, 100.9, 100.7, 87.0, 76.5, 72.6, 67.9, 49.1, 36.0, 25.8, 24.9, 22.3, 17.4, 13.9, 1.0, 0.6, 0.1, -0.2.

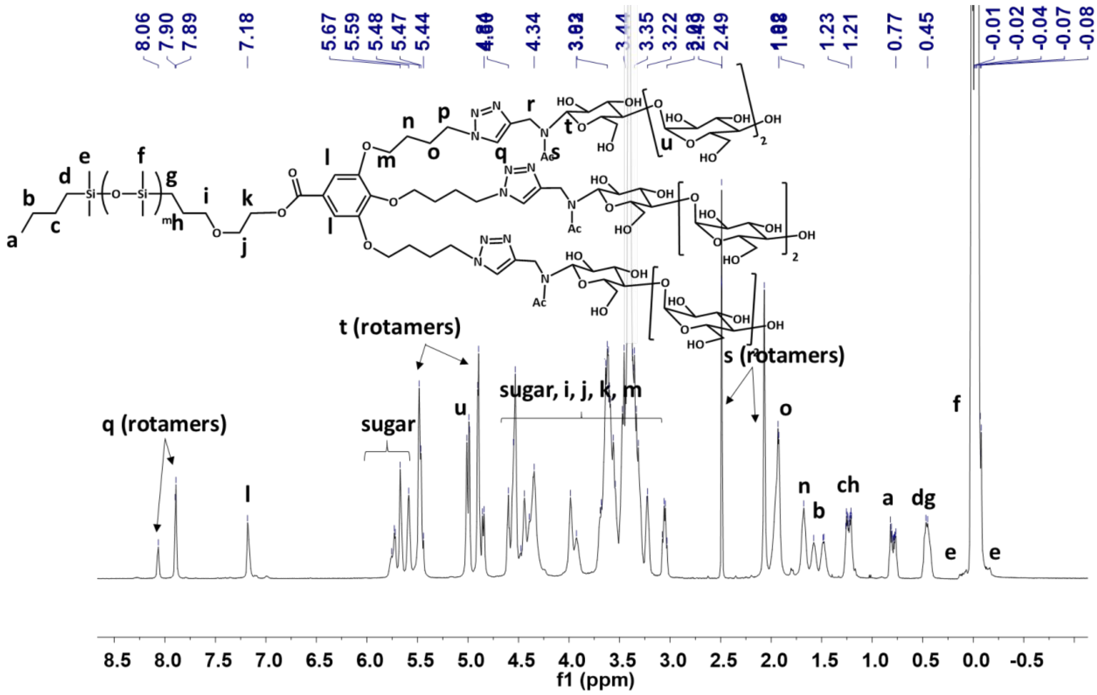

Figure S22. ^{1}H NMR spectrum of **AB3** in d_6-DMSO.

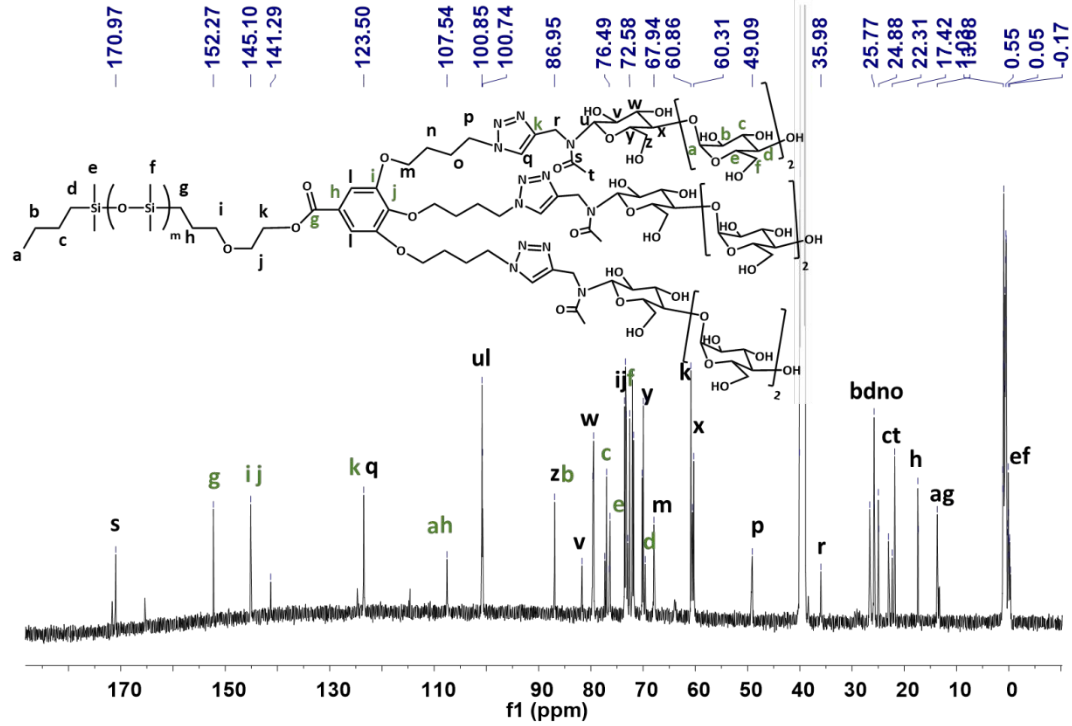

Figure S23. ^{13}C NMR spectrum of **AB3** in d_6-DMSO.

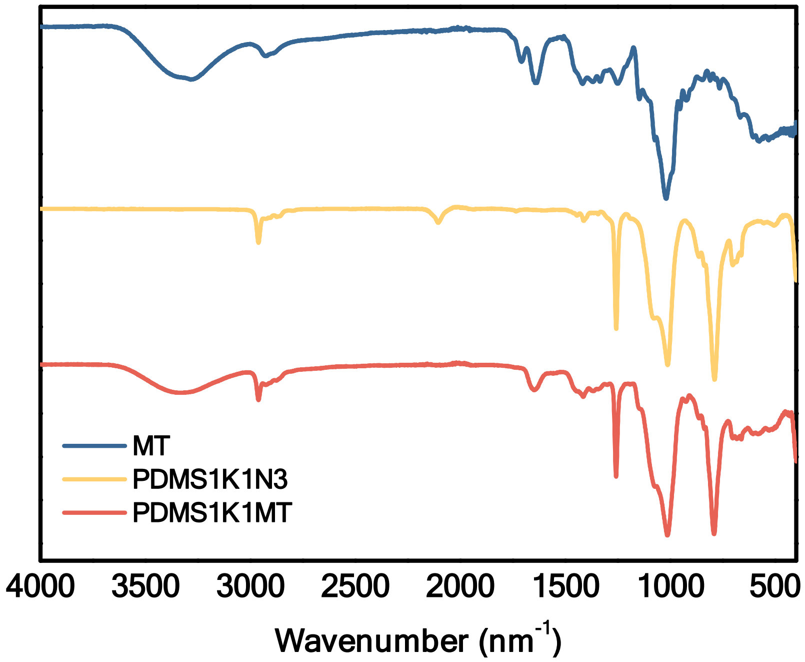

Figure S24. FT-IR spectra of **alkyne-MT**, **PDMS1N3**, and **AB.**

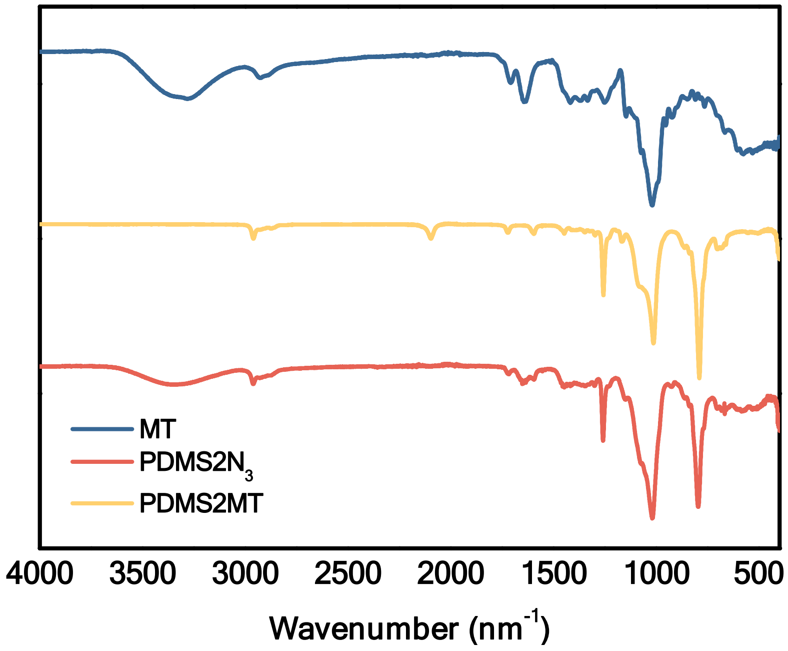

Figure S25. FT-IR spectra of **alkyne-MT**, **PDMS2N3**, and **AB2.**

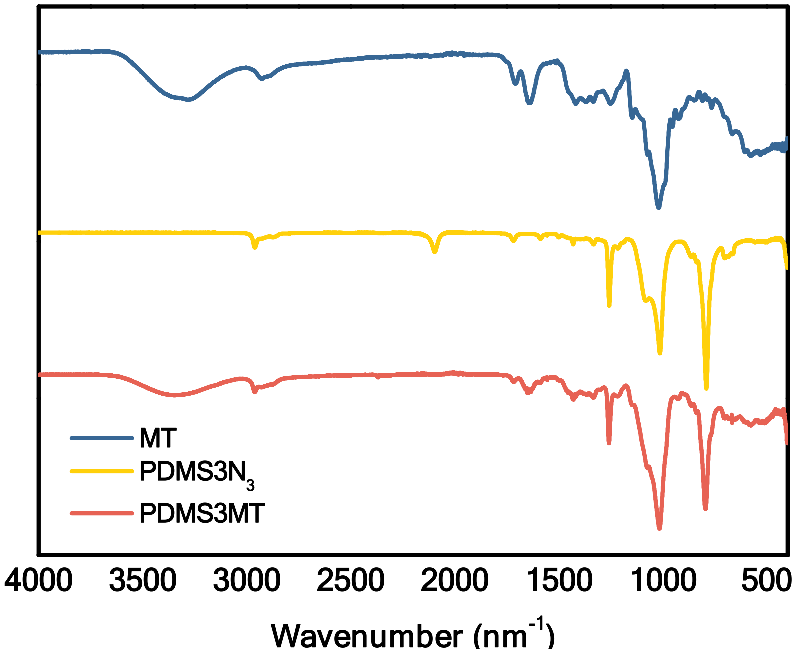

Figure S26. FT-IR spectra of **alkyne-MT**, **PDMS3N3**, and **AB3**.

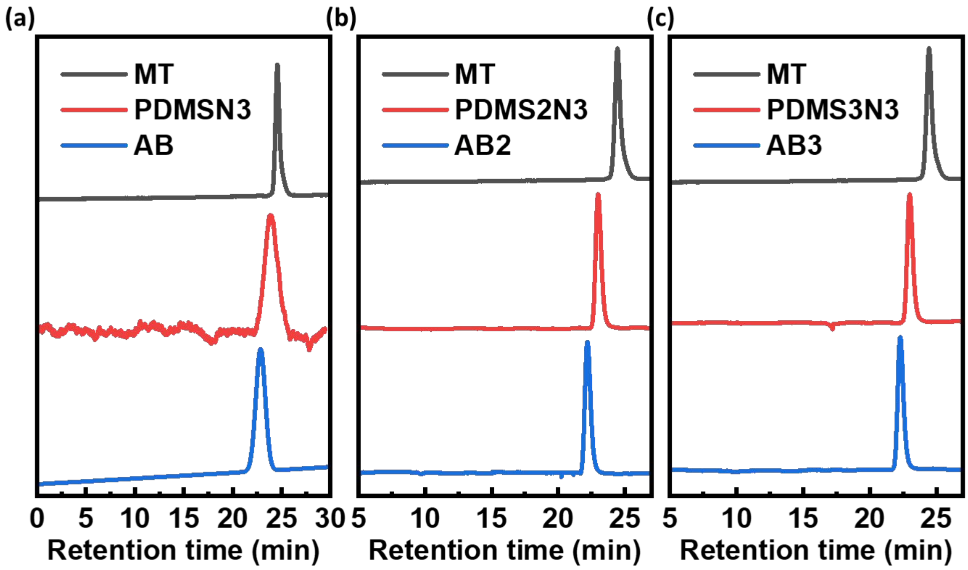

Figure S27. SEC profiles of (a) **AB**, (b) **AB2**, and (c) **AB3** with their precursors. The measurements were conducted with DMF as an eluent.

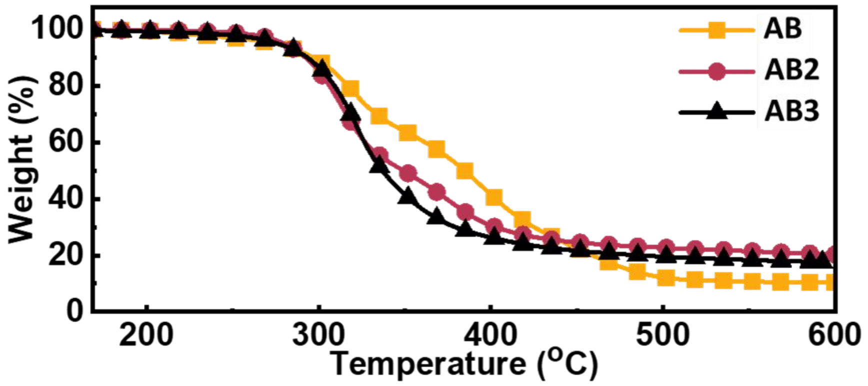

Figure S28. TGA profiles of oligosaccharide-based BCPs studied using a heating rate of 10 °C min^{-1} over a temperature range of 100 to 600 °C under an air gas flow.

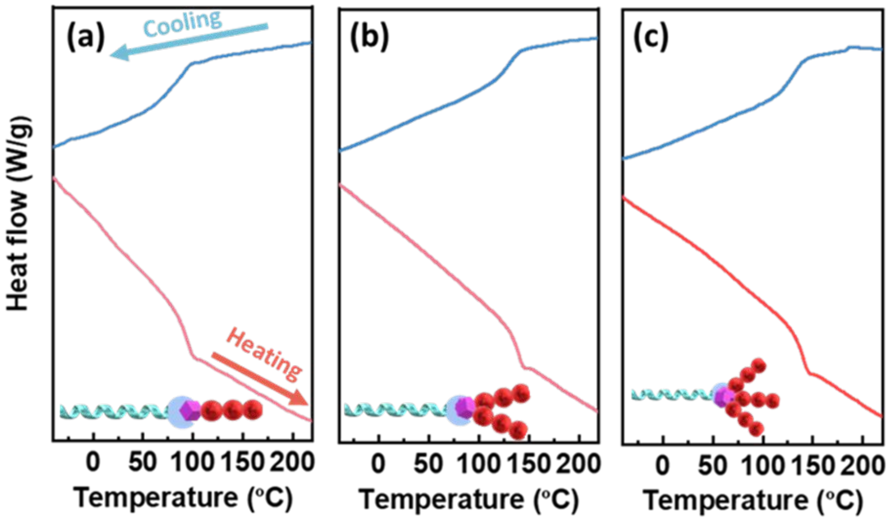

Figure S29. DSC profiles of oligosaccharide-based BCPs studied at heating and cooling rates of 10 °C min^{-1} over a temperature range of –80 to 240 °C under nitrogen flow: (a) **AB**, (b) **AB2**, and (c) **AB3**. Note that the blue line is a cooling curve, and the red is a heating curve.

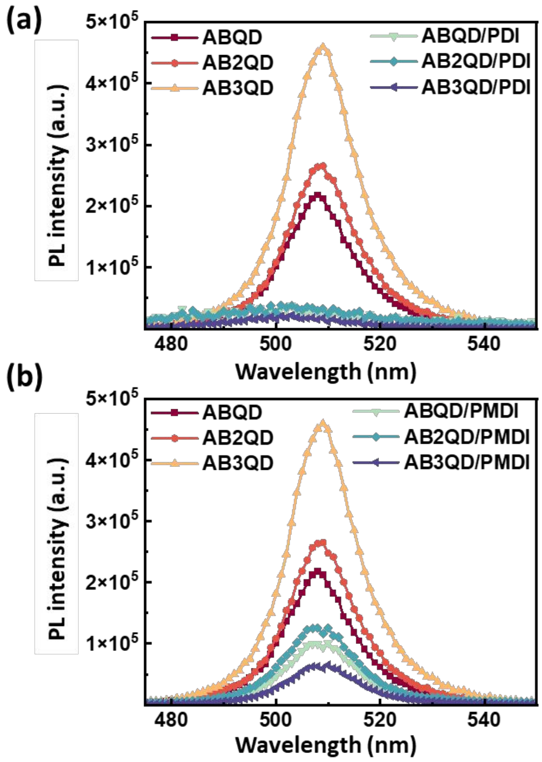

Figure S30. PL emission spectra of the channel materials: (a) **PDI** and (b) **PMDI** and their bilayered films with BCP/QDs.

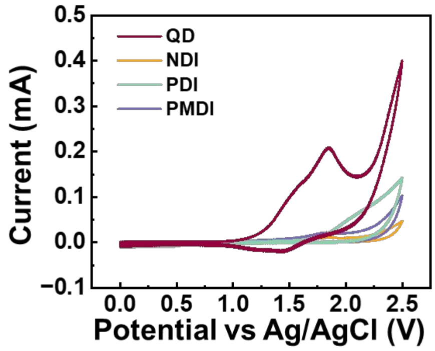

Figure S31. CV profiles of **NDI, PDI, PMDI,** and QD in the oxidation range from 0 to 2.5 V.

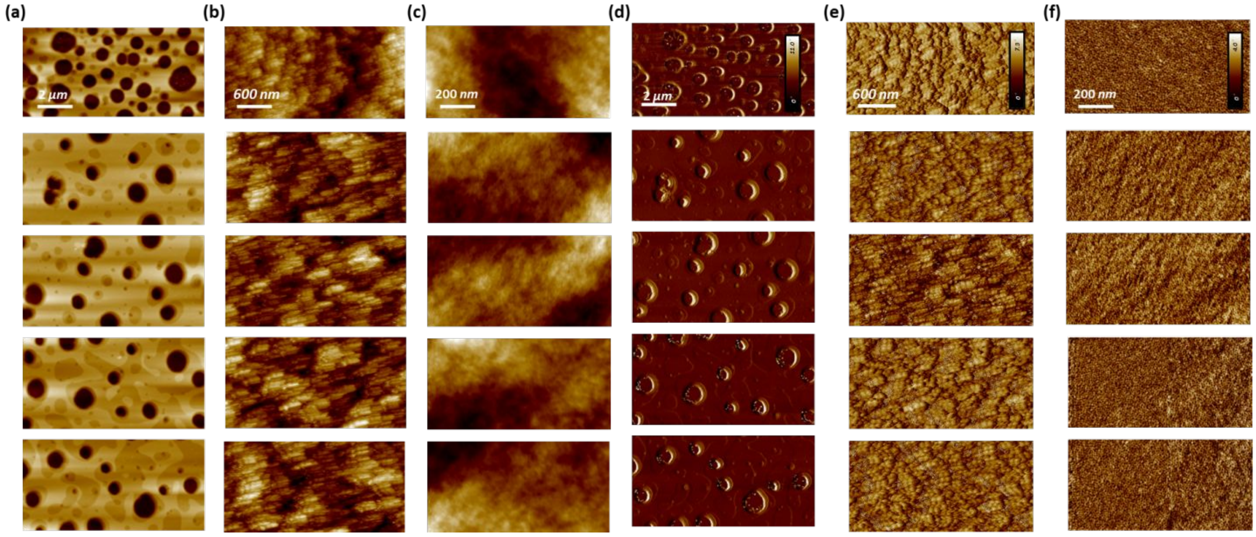

Figure S32. AFM (a-c) height and (d-f) phase image of pure (a,d) **AB**, (b,e) **AB2**, and (c,f) **AB3** thin films with a specific scale bar.

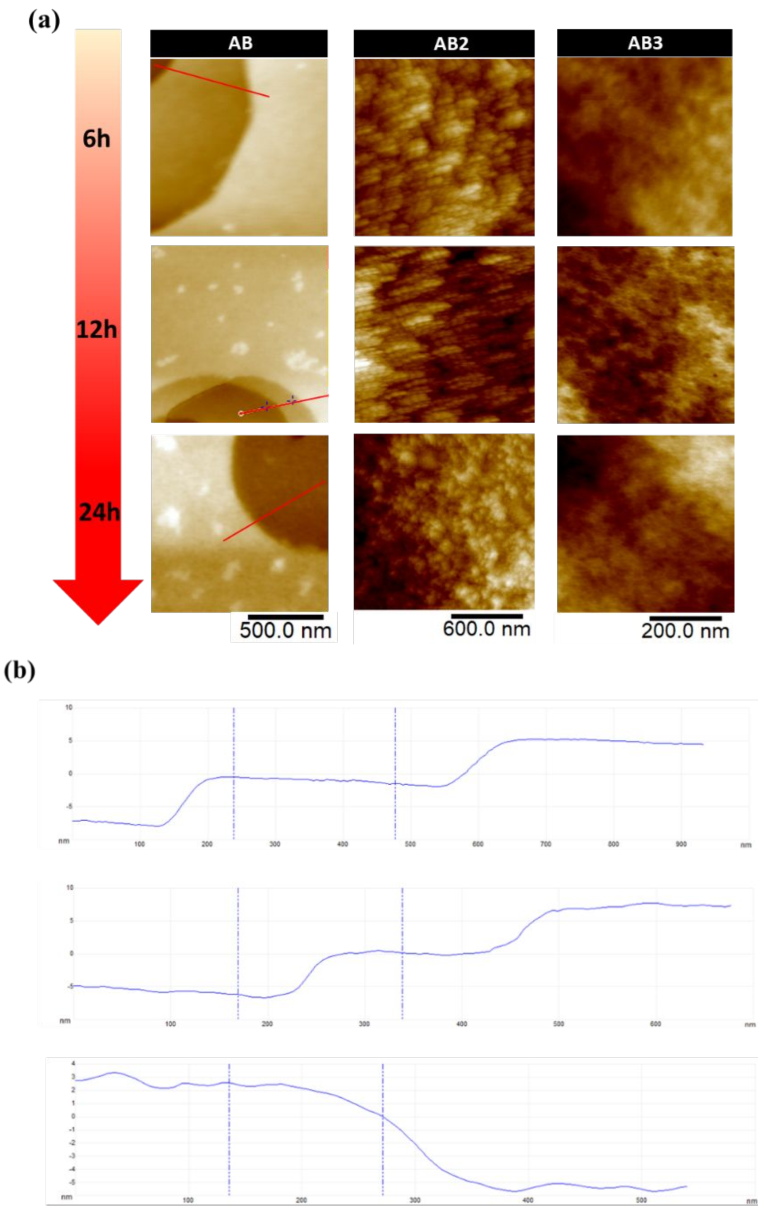

Figure S33. (a) AFM height images of **AB**, **AB2**, and **AB3** after thermal annealing at 80 °C for 6, 12, and 24 hours. (b) The corresponding cross-sectional height profiles of **AB** along the red line.

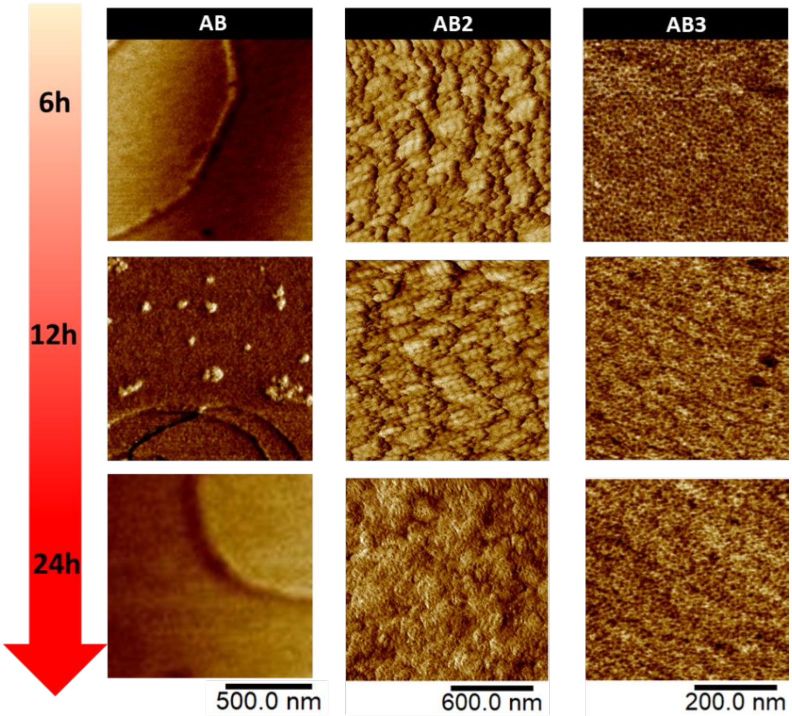

Figure S34. AFM phase images of **AB**, **AB2**, and **AB3** with thermal annealing times of 6, 12, and 24 hours at 80 °C.

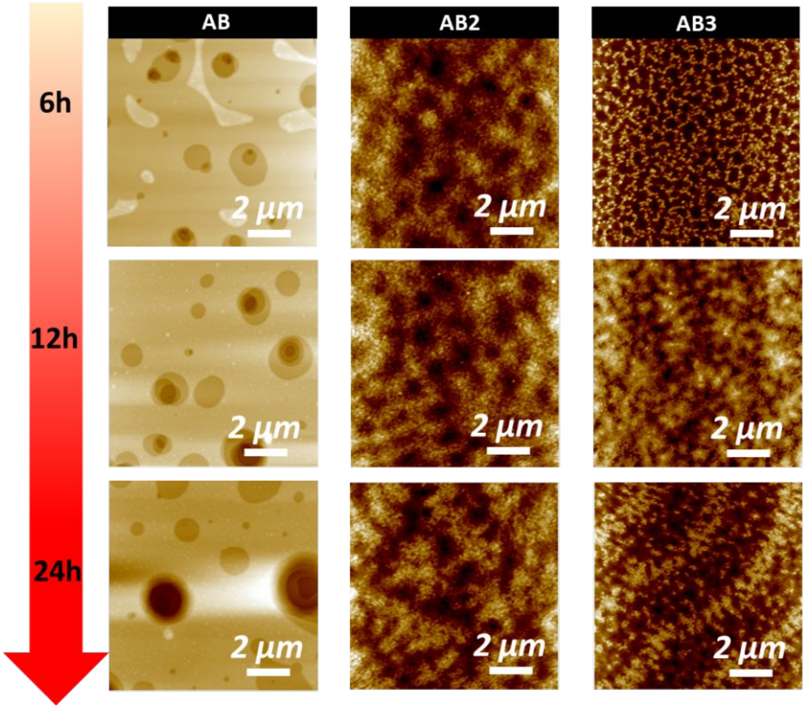

Figure S35. AFM height images of **AB**, **AB2**, and **AB3** after thermal annealing at 80 °C for 6, 12, and 24 hours, acquired over a 10 × 10 μm² area.

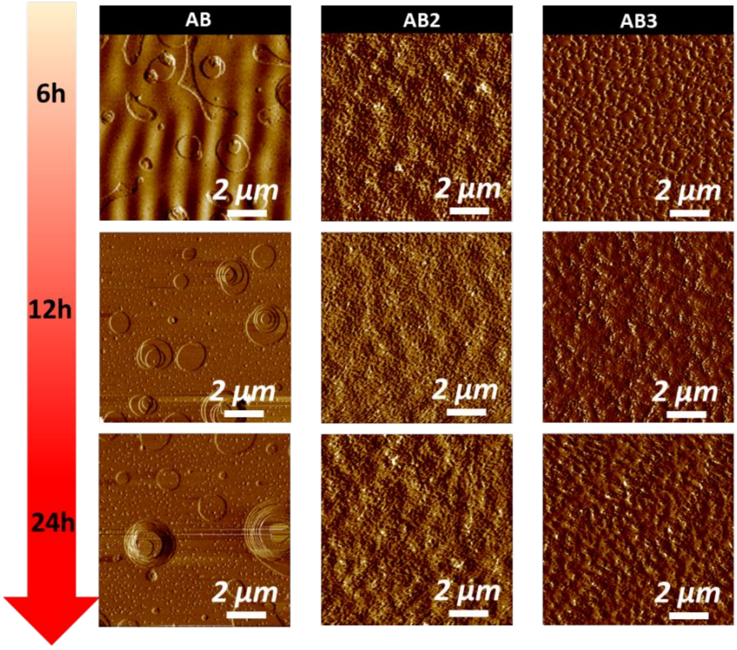

Figure S36. AFM phase images of **AB**, **AB2**, and **AB3** after thermal annealing at 80 °C for 6, 12, and 24 hours.

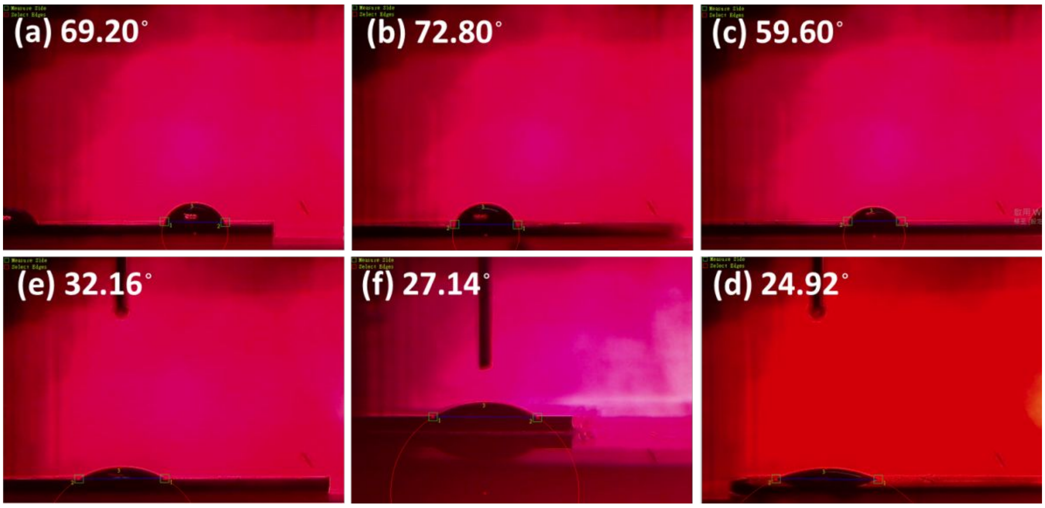

Figure. S37. Surface energy calculation of (a,d) **AB**, (b,e) **AB2**, and (c,f) **AB3** implemented with the test liquids are (a-c) diiodomethane and (d-f) ethylene glycol. The surface energy calculation was based on the Owens–Wendt model as the equation: $\sigma_l \times (1 + \cos\theta) / 2 \times (\sigma_l^d)^{0.5} = (\sigma_s^p)^{0.5} \times (\sigma_l^p / \sigma_l^d)^{0.5} + (\sigma_s^d)^{0.5}$ and equation $\sigma_s = \sigma_s^p + \sigma_s^d$, where θ is the contact angle between the solid phase and the test liquid. σ_s^p and σ_s^s are the polar and dispersion components of surface energy for the solid phase. σ_l^p and σ_l^s are the test liquid's polar and dispersion surface energy components.

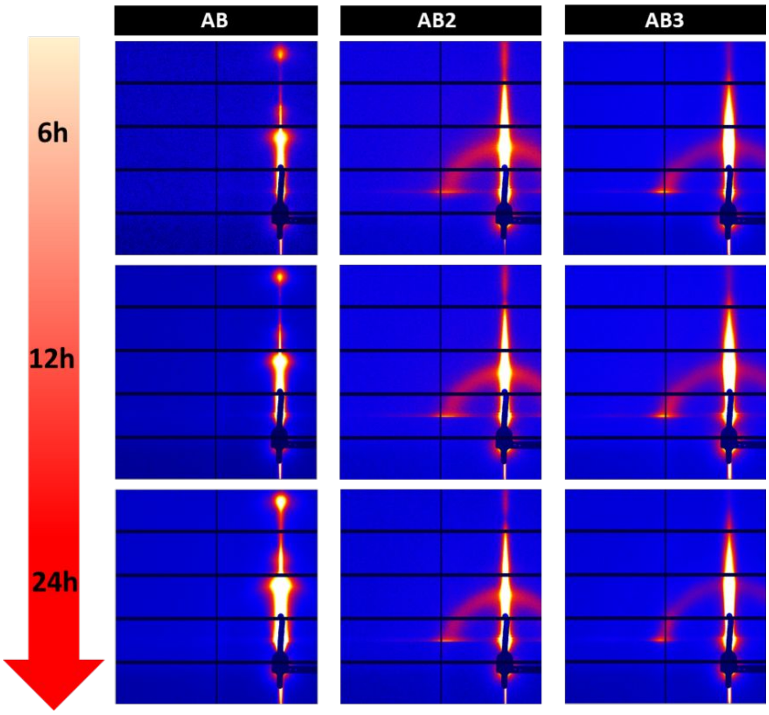

Figure S38. GISAXS 2D profiles of pure **AB**, **AB2**, and **AB3** through thermal annealing thin films with 6, 12, and 24 hours.

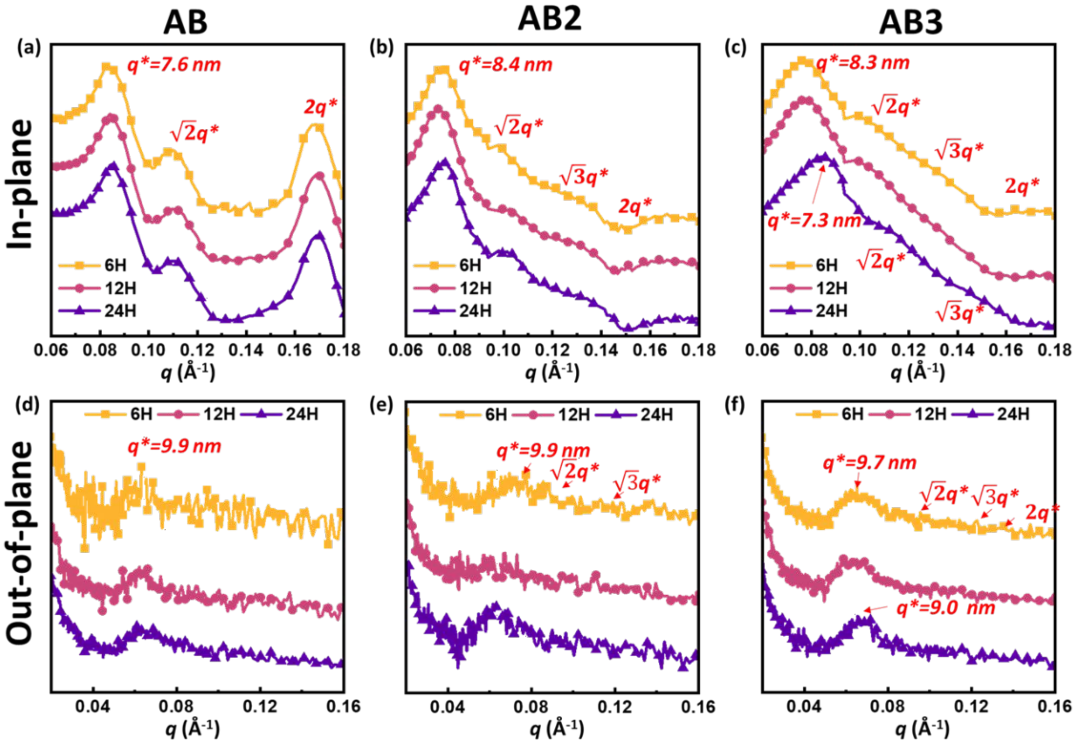

Figure S39. GISAXS 1D profiles of oligosaccharide-based BCP composite films: (a–c) in-plane and (d–f) out-of-plane directions for (a,d) **AB**, (b,e) **AB2**, and (c,f) **AB3.**

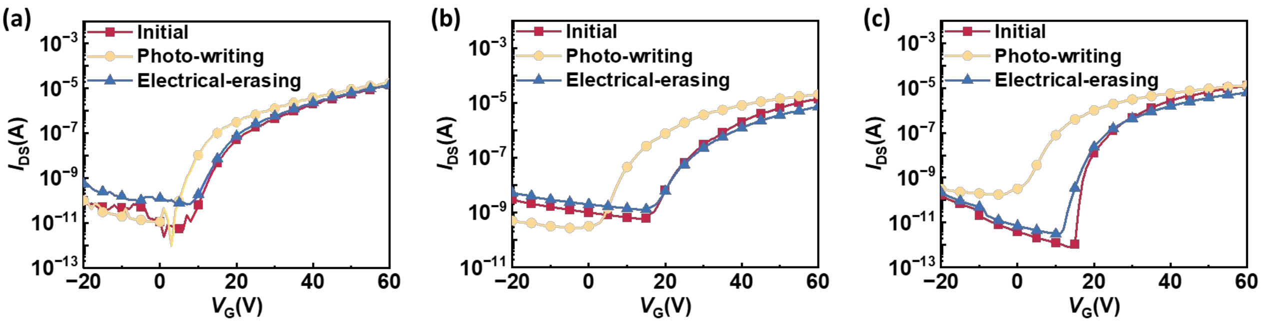

Figure S40. Transfer curves of pure (a) **AB**, (b) **AB2,** and (c) **AB3** with **NDI** in phototransistor memory devices under a fixed V_{DS} = 60 V.

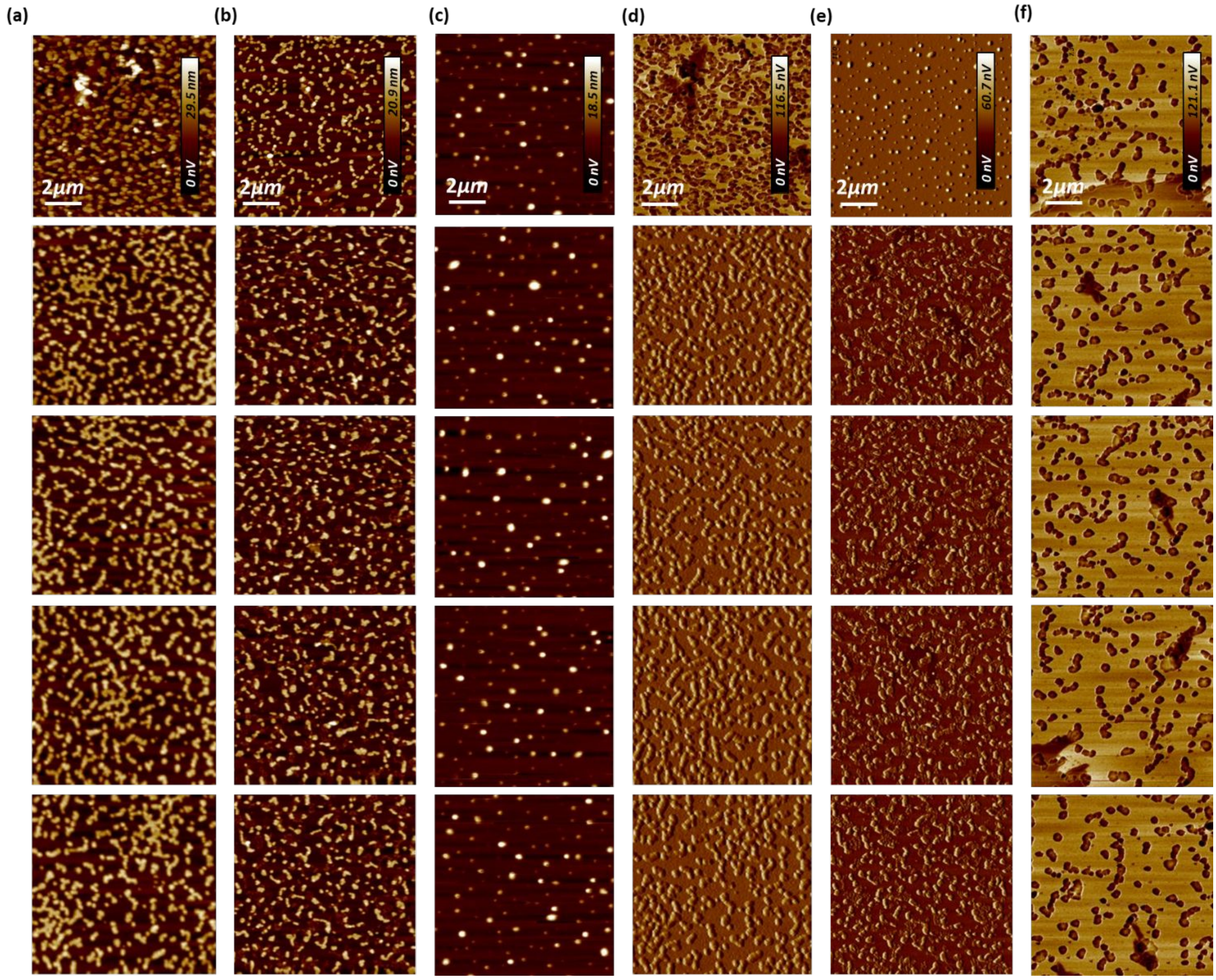

Figure S41. AFM (a-c) height and (d-f) phase image of (a,d) **ABQD**, (b,e) **AB2QD**, and (c,f) **AB3QD** composite films with a specific scale bar of 2 μm.

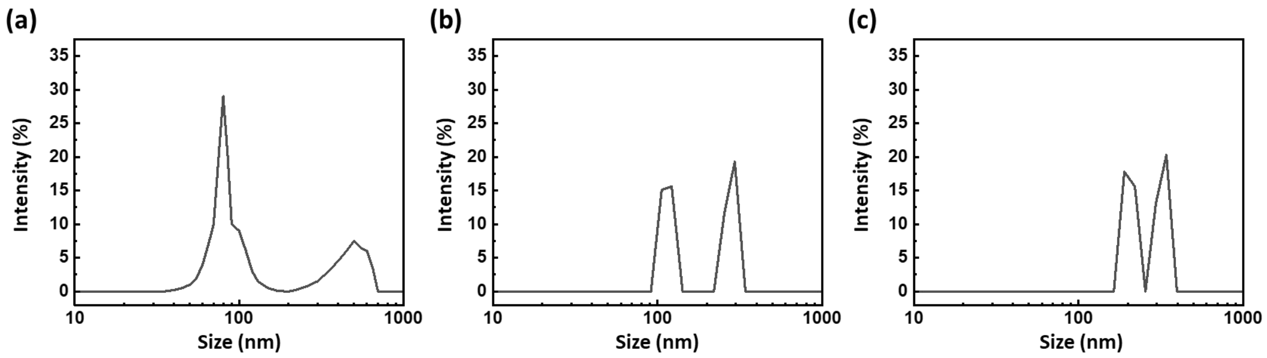

Figure S42. Intensity-based DLS size distribution profiles of the (a) **ABQD**, (b) **AB2QD**, and (c) **AB3QD** in the toluene solution.

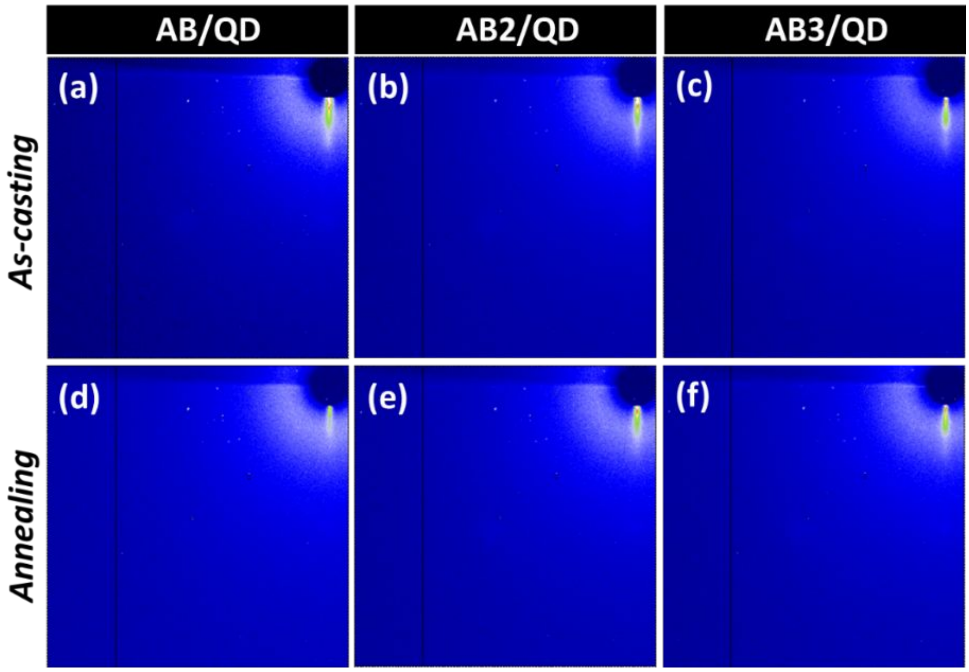

Figure S43. GIWAXS 2D patterns of (a,d) **AB/QD**, (b,e) **AB2/QD**, and (c,f) **AB3/QD** of the (a-c) pristine thin films and (d-f) thermal annealed thin films.

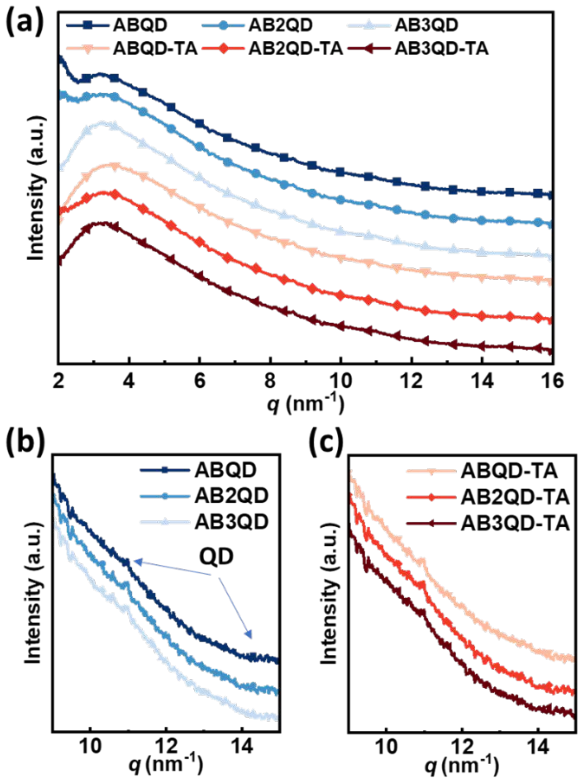

Figure S44. GIWAXS 1D profiles of the BCP composite films: (a) the comparison of the pristine and thermal annealed (TA) thin films with an enlarged view of the q-range over 9 to 15 nm^{-1} for the (b) pristine and (c) TA films.

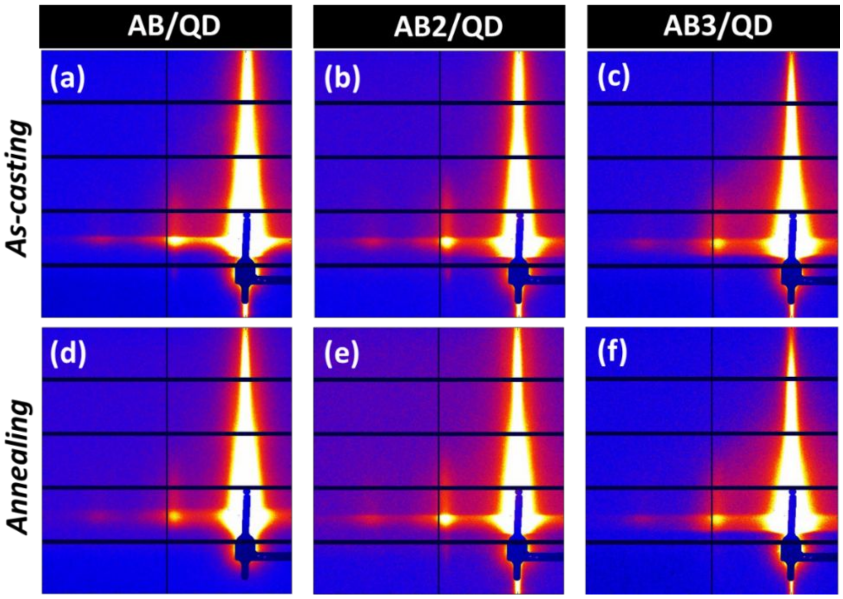

Figure S45. GISAXS 2D patterns of (a,d) **AB/QD**, (b,e) **AB2/QD**, and (c,f) **AB3/QD** of the (a–c) as-casted and (d–f) thermal annealed thin films.

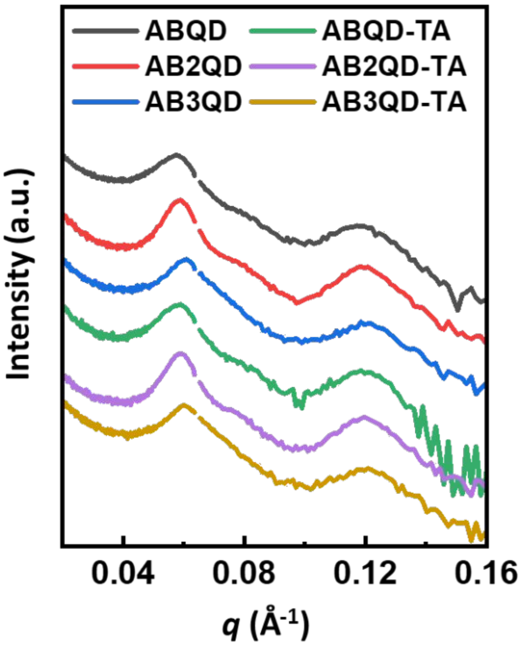

Figure S46. GISAXS 1D profiles of hybrid BCP composite films with their pristine and thermally annealed thin films.

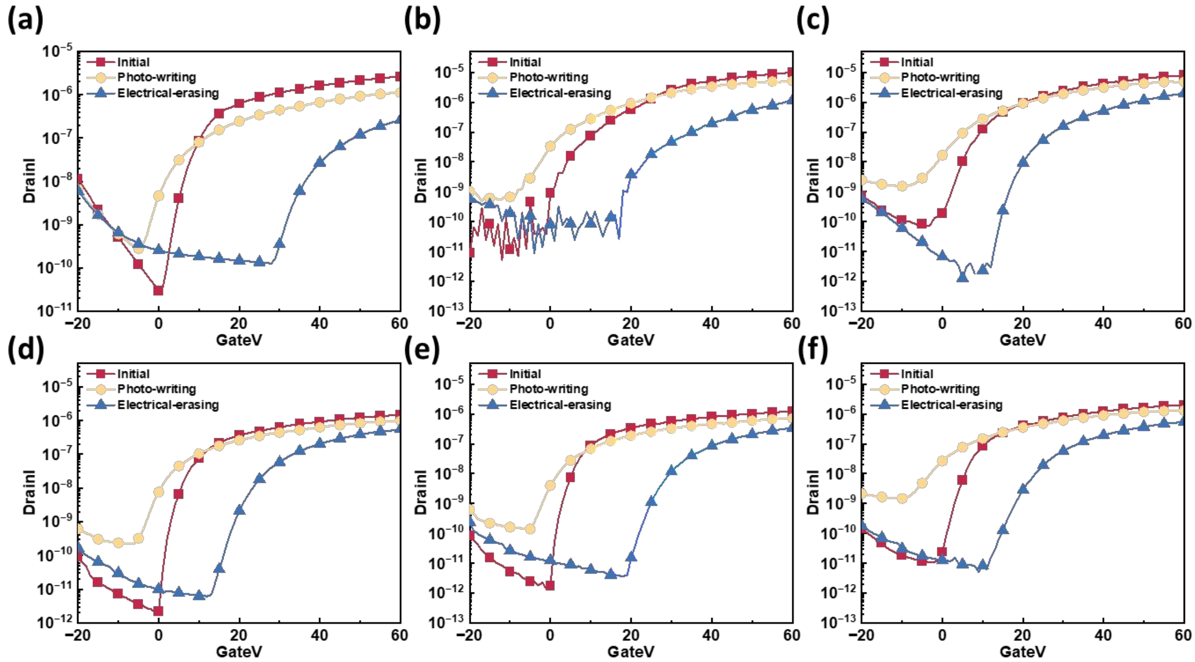

Figure S47. Transfer curves of (a,d) **AB/QD**, (b,e) **AB2/QD**, and (c,f) **AB3/QD** at the ratio of (a–c) 9/1 and (d–f) 7/3 in phototransistor memory devices with **NDI** as the channel under a fixed V_{DS} = 60 V.

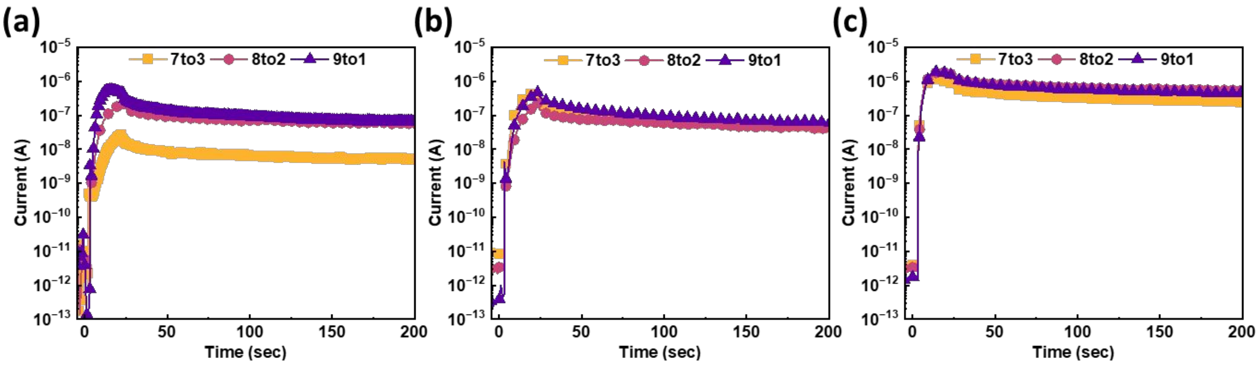

Figure S48. Transient curves of (a) **AB/QD**, (b) **AB2/QD**, and (c) **AB3/QD** at the different ratio with **NDI** in phototransistor memory devices under a fixed V_{DS} = 60 V.

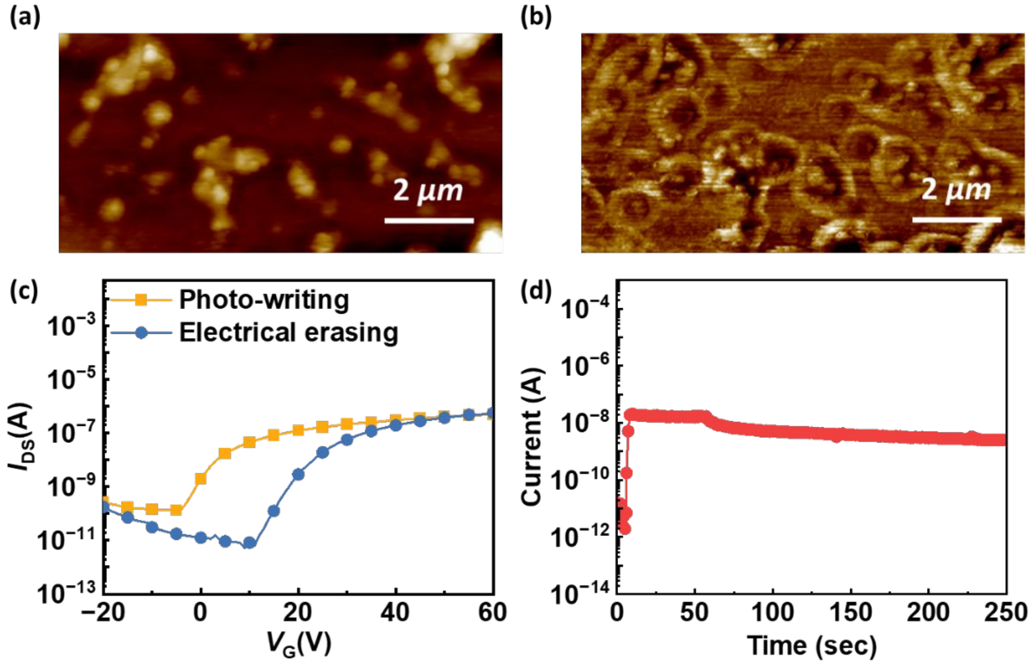

Figure S49. AFM (a) height and (b) phase images of **ABQD** at the ratio of 8/2 with the 70 nm thickness across 5 × 10 μm. (c) Transfer and (d) transient curves based on an **NDI** channel were measured at V_{DS} = 60 V, with photowriting under 365-nm light for 60 s and electrical erasing at V_{GS} = 60 V for 1 s.

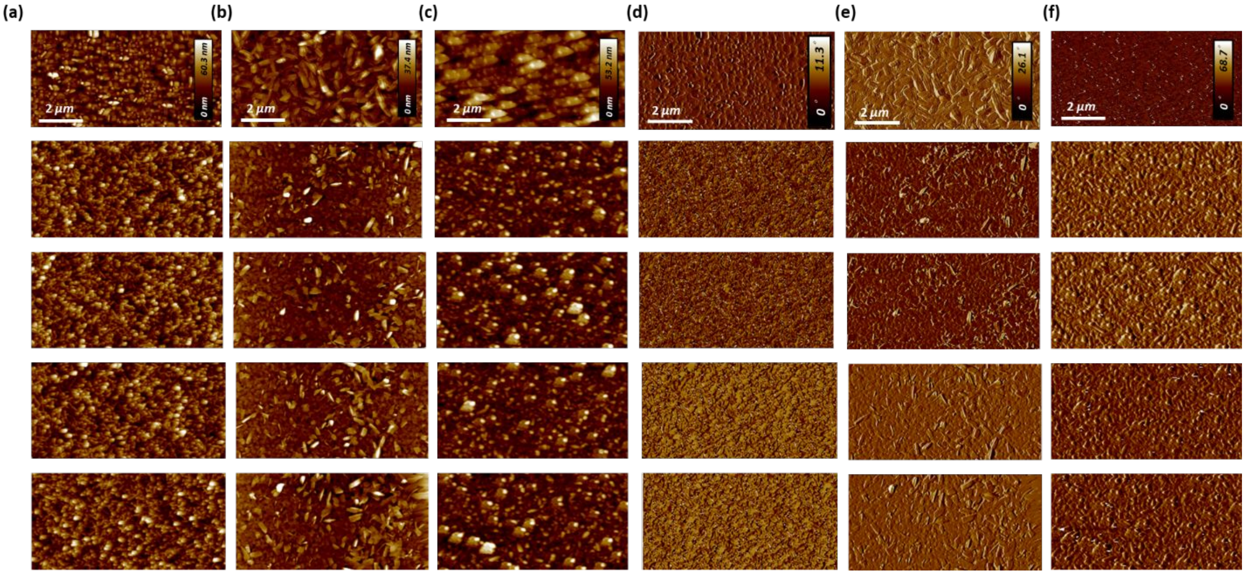

Figure S50. AFM (a-c) height and (d-f) phase image of (a,d) **PMDI**, (b,e) **NDI**, and (c,f) **PDI** on the AB3QD composite films with a specific scale bar of 2 μm.

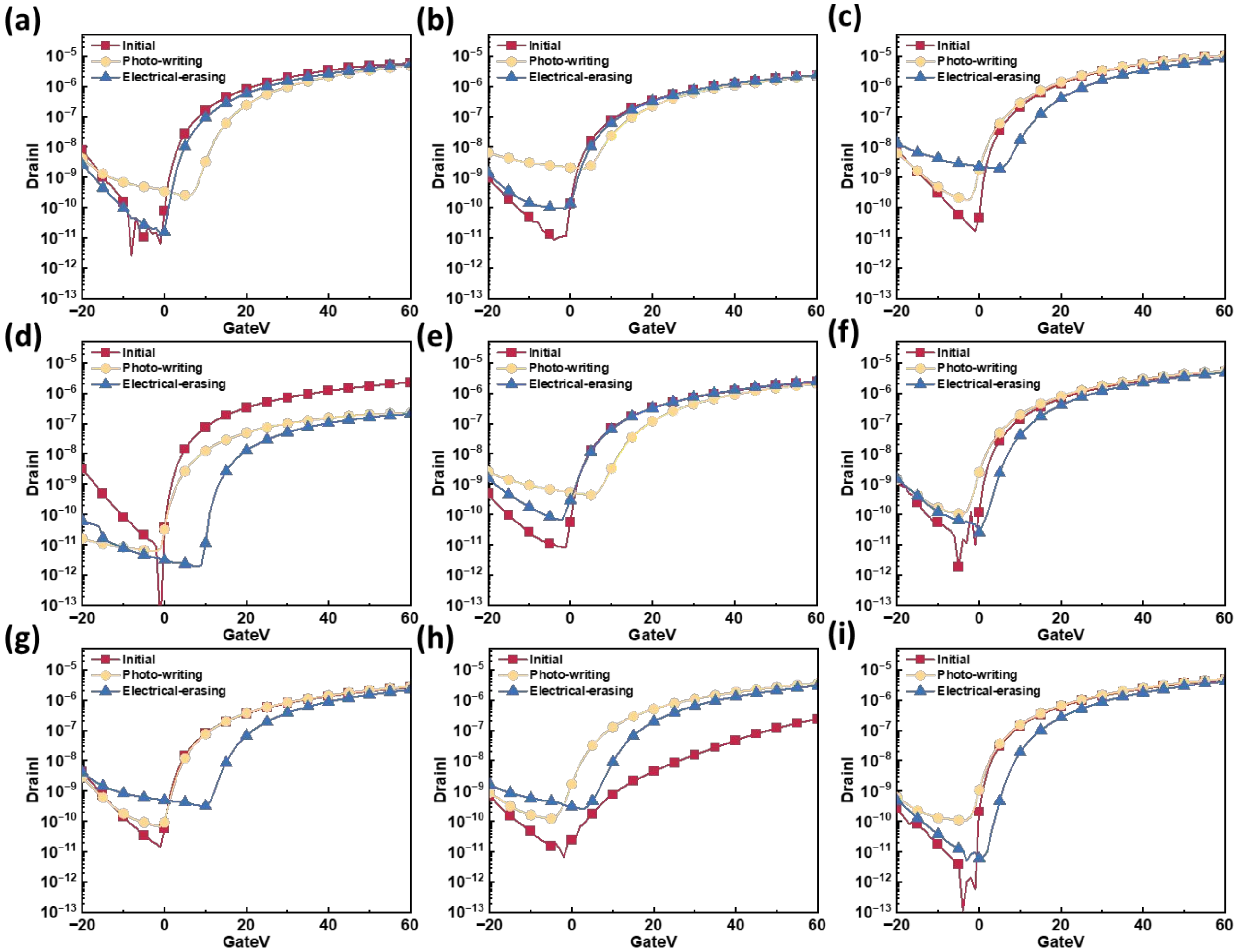

Figure S51. Transient curves of (a,d,g) **AB/QD**, (b,e,h) **AB2/QD**, and (c,f,i**) AB3/QD** at the ratio of (a–c) 9/1, (d–f) 8/2, and (g–i) 7/3 with **PDI** in phototransistor memory devices under a fixed V_{DS} = 60 V.

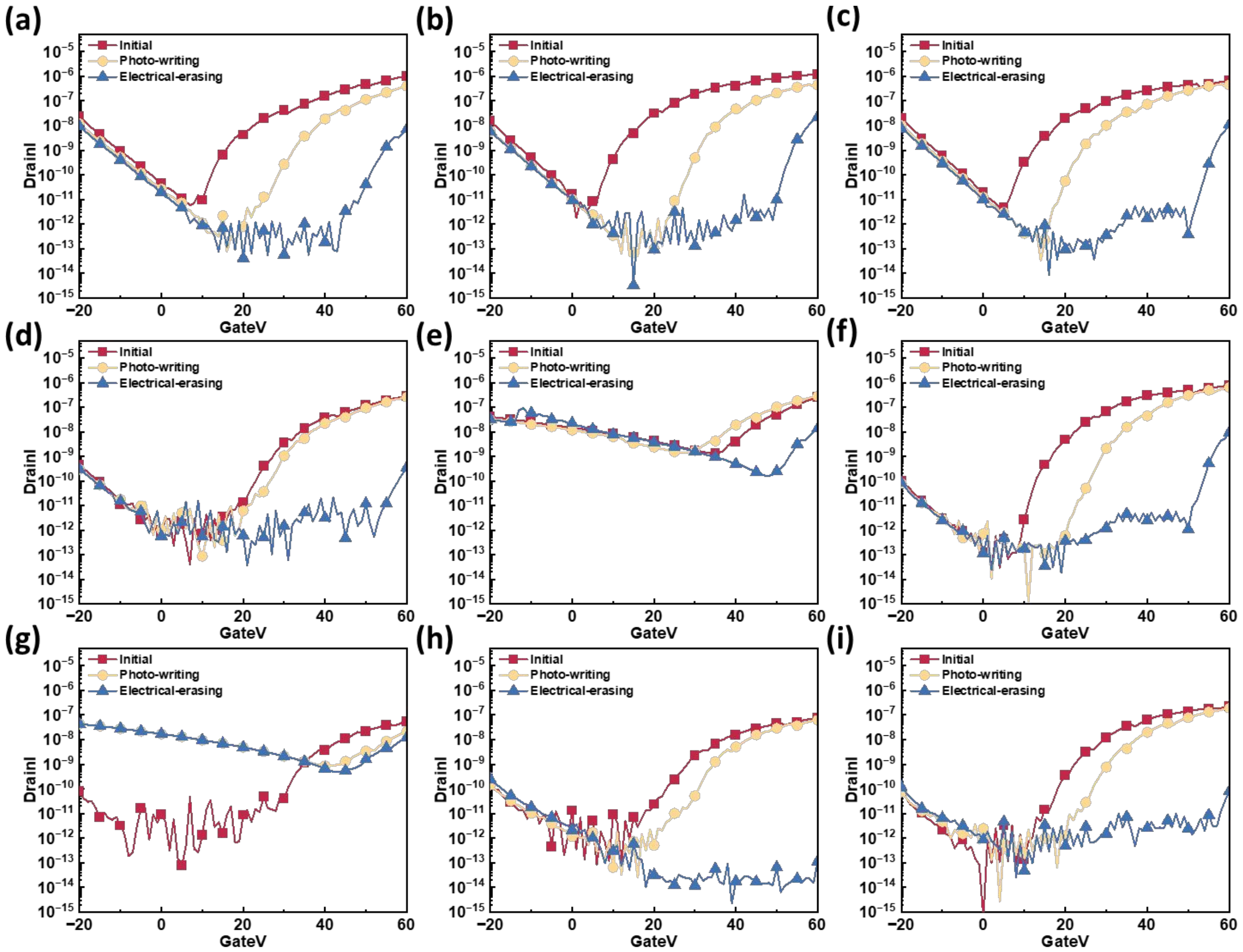

Figure S52. Transient curves of (a,d,g) **AB/QD**, (b,e,h) **AB2/QD**, and (c,f,i) **AB3/QD** at the ratio of (a–c) 9/1, (d–f) 8/2, and (g–i) 7/3 with **PMDI** in phototransistor memory devices at V_{DS} = 60 V.

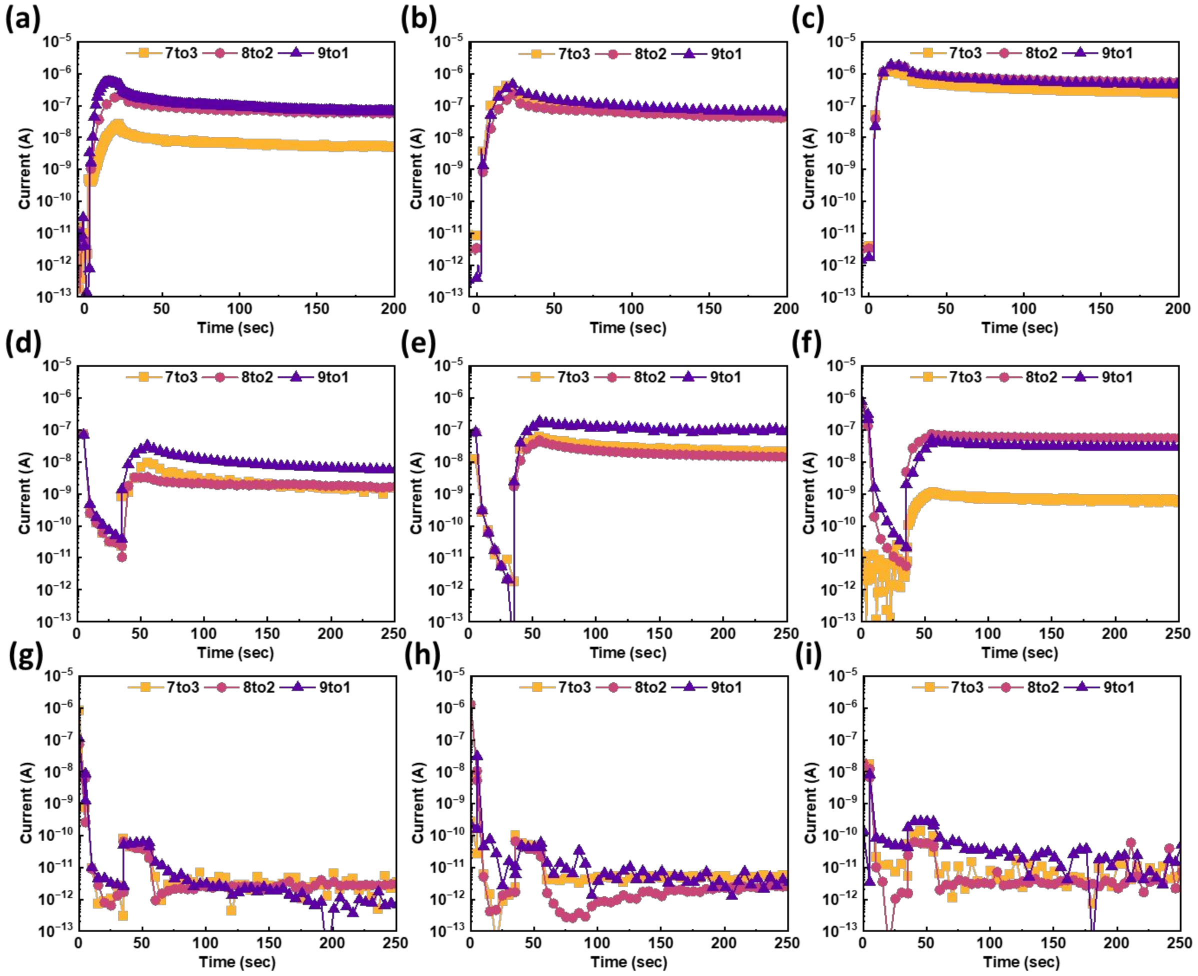

Figure S53. Transient curves of (a,d,g) **AB/QD**, (b,e,h) **AB2/QD**, and (c,f,i) **AB3/QD** at the different ratio with (a–c) **NDI**, (d–f) **PDI**, and (g–i) **PMDI** in phototransistor memory devices at V_{DS} = 60 V.

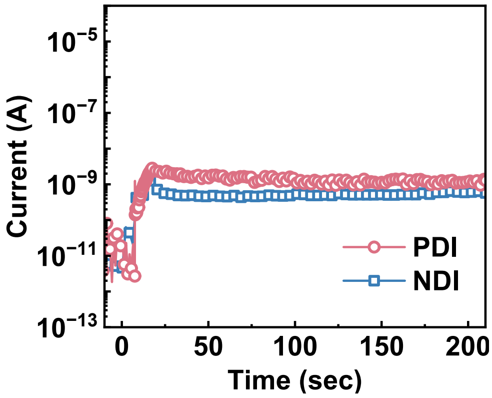

Figure S54. The comparison of transient curves of **AB3QD** at the ratio of 8/2 with **NDI** and **PDI** in phototransistor memory devices under a fixed V_{DS} = 60 V under 450 nm. Note the orange shadow indicates the light-illumination time of 10 s.

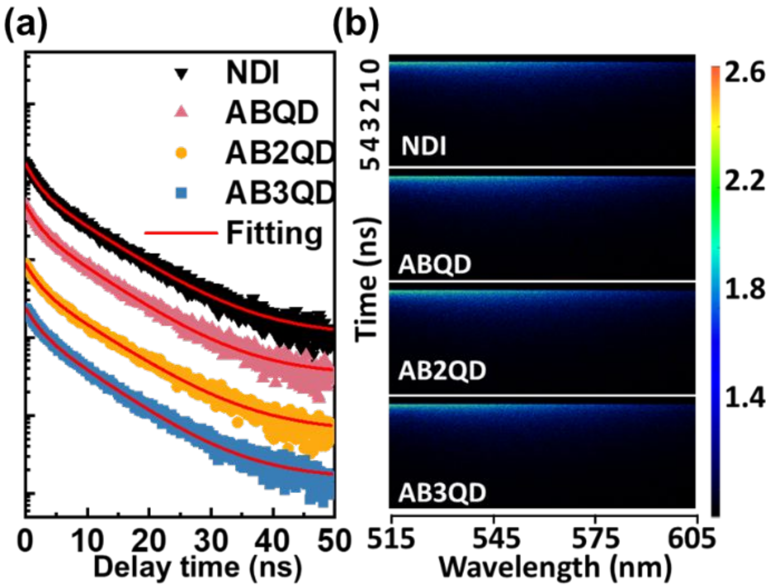

Figure S55. TRPL measurement of hybrid BCP/QD composite films: (a) 1D profiles decay profiles and (b) 2D TR-PL profiles of pristine **NDI**, and **ABQD/NDI**, **AB2QD/NDI**, and **AB3QD/NDI** with the excited wavelength of 510 nm.

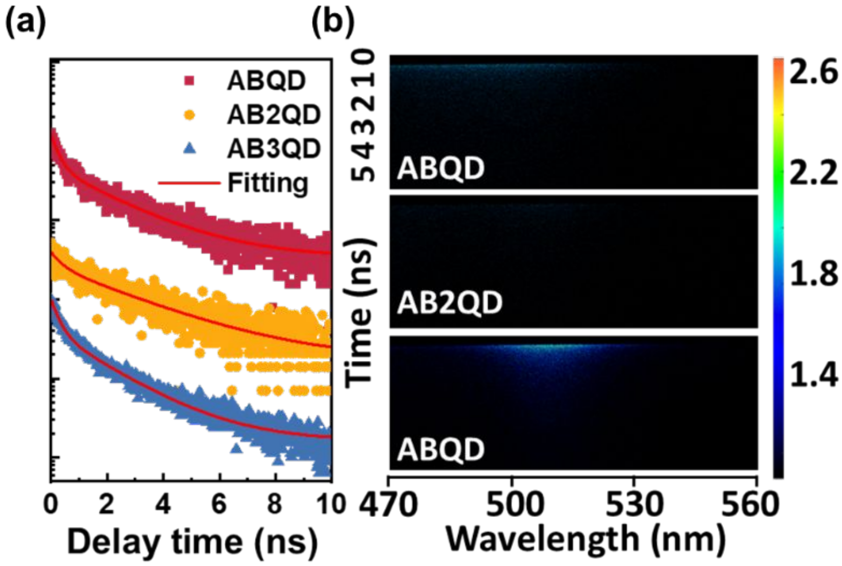

Figure S56. TRPL measurement of hybrid BCP/QD composite films: (a) 1D profiles decay profiles and (b) 2D TR-PL profiles of pristine **PMDI**, and **ABQD/PMDI**, **AB2QD/PMDI**, and **AB3QD/PMDI** with the excited wavelength of 510 nm.

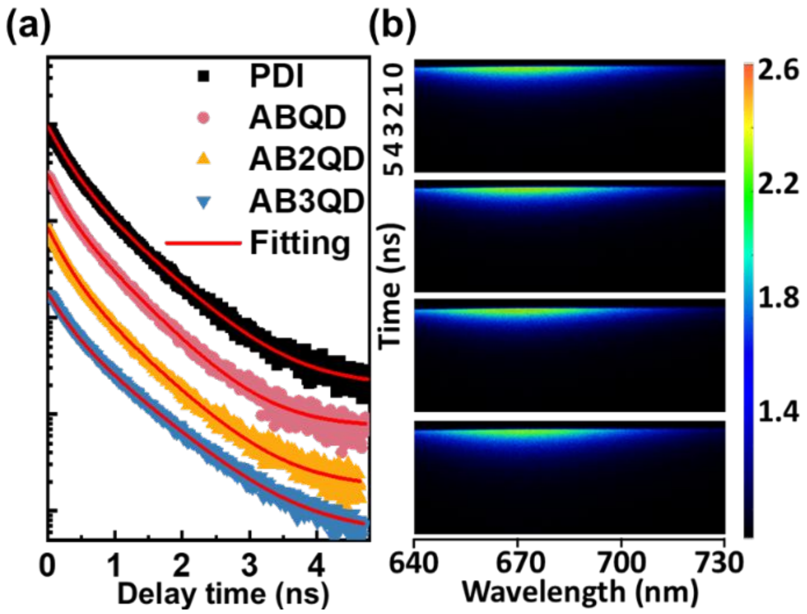

Figure S57. TRPL measurement of hybrid BCP/QD composite films: (a) 1D profiles decay profiles and (b) 2D TR-PL contour plots of pristine **PDI**, and **ABQD/PDI**, **AB2QD/PDI**, and **AB3QD/PDI** with the excited wavelength of 630 nm.
